# Genetic gains in forage sorghum for adaptive traits for non - conventional area through multi-trait-based stability selection methods

**DOI:** 10.3389/fpls.2024.1248663

**Published:** 2024-03-07

**Authors:** Partha Pratim Behera, Avinash Singode, B. Venkatesh Bhat, Venkateswarlu Ronda, Nayanmoni Borah, Harendra Verma, Labhya Rani Gogoi, Jyoti Lekha Borah, Prasanta Kumar Majhi, Niharika Saharia, Ramendra Nath Sarma

**Affiliations:** ^1^ Department of Plant Breeding and Genetics, Assam Agricultural University, Jorhat, Assam, India; ^2^ Department of Plant Breeding, Indian Council of Agricultural Research (ICAR) - Indian Institute of Millets Research, Hyderabad, Telangana, India; ^3^ Department of Bio-chemistry, Indian Council of Agricultural Research (ICAR) - Indian Institute of Millets Research, Hyderabad, Telangana, India; ^4^ Assam Don Bosco University, Guwahati, Assam, India; ^5^ Nagaland Centre, ICAR Research Complex for North Eastern Hill (NEH) Region, Dimapur, Nagaland, India; ^6^ Department of Plant Breeding and Genetics, Regional Research and Technology Transfer Station, Odisha University of Agriculture and Technology, Odisha, India

**Keywords:** forage sorghum, genotype-by-environment interaction (GEI), genetic gain under selection, multi trait based stability indices, variety development

## Abstract

**Introduction:**

In the Asian tropics, unpredictable weather increases the risk of abiotic stresses in sorghum areas, making it harder to meet predicted demand. Genotype-by environment interaction (GEI) and the lack of an effective multi-trait-based selection approach make it challenging to breed climateresilient forage sorghum that adapts to nonconventional areas.

**Methods:**

The present investigation carried out to estimate genetic parameters, inter trait associations, genetic gain under selection (SGs) of 95 diverse forage sorghum genotypes. Fourteen forage yield and other secondary traits were evaluated at five different growing seasons at two locations. Negative and positive genetic gains under selection were estimated across different growing seasons including Kharif, Rabi and Summer in the year 2020 and 2021.

**Results and discussion:**

The GEI effects were significant (P < 0.001) for all the studied traits. The multi trait based stability indices have been said to assist breeders in ensuring sustained progress in primary traits likeforage yield without sacrificing genetic advancement in secondary traits. Fourteen genotypes were selected through each evaluation methods including genotype – ideotype distance index (MGIDI), multi-trait stability index (MTSI), multi-trait stability and mean performance (MTMPS) and multi-trait index based on factor analysis and genotype-ideotype distance (FAIBLUP Index), assuming 15% selection intensity. According to MGIDI, the selected genotypes exhibited desired positive genetic gains for dry forage yield per plant, inter-nodal length, green forage yield per plant, and plant height and negative genetic gains for days to 50% flowering. The strength and weakness plot is a potential graphical tool as portrayed by MGIDI, to identify and develop desirable genotype for particular environment. Two genotypes, G36 (302B) and G89 (348B) were found to be common across all four evaluation methods based on all the studied traits.

**Background:**

Multi-trait stability evaluation approaches are reliable and accessible for selecting multiple traits under varied testing environments with low multicollinearity issues. These tools proved effective in enhancing selection strategies and optimising breeding schemes for the development of climate-resilient forage sorghum genotypes. The aforementioned genotypes were found to be the most reliable, high-yielding, and earlymaturing and could be suggested for variety and hybrid development and ideotype breeding programmes to ensure the food and nutritional security.

## 1 Introduction

Sorghum (*Sorghum bicolor* L. Moench) is a versatile cereal crop cultivated for grains, forage and many industrial products. It ranks fifth in importance after wheat, rice, maize, and barley (Ritter et al., 2007; Motlhaodi et al., 2014). Sorghum is cultivated for food grains in most of the arid and semi-arid regions of Asia and Africa. Whereas, in the western countries it is cultivated for green forage and feed (Almas et al., 2015; Getachew et al., 2016; Aruna and Cheruku, 2019; Mundia et al., 2019). In most of the resource poor countries sorghum stalk is fed to animals during the lean period of the year. Sorghum produces excellent forage, the forage quality viz. digestibility is on-par with maize. Based on the number of cuts two types of forage sorghums are grown, single cut and multi-cut. Sorghum is also found better for silage and pellet making which can be stored for a long time and also it improves digestibility. The sorghum dry stalk after harvesting panicles is stacked and is fed to animals. It is the most efficient use of dual type of sorghum which is grown for grains and dry fodder (Aruna and Cheruku, 2019).

North-Eastern India is a non-conventional area for forage sorghum. Rice is predominantly grown in the region. The other vegetation in the region is less amenable for dairy and meat industry. The milk and animal products demands are linear to the fodder demand in the region. The year-round fodder demand can be met by cultivating a crop like sorghum which is high yielding, drought tolerant, wide adaptability and also photo-insensitivity types available (Wolabu and Tadege, 2016). Looking into the prospects of forage sorghum in North-Eastern (NE) India, breeding program was initiated. The limited availability of genetically diverse resources tailored for forage sorghum development has hindered progress in developing varieties and hybrids. No high-yielding forage sorghum varieties or hybrids have been tested for suitability in NE India, including Assam (Bora et al., 2020). The development of stable, high-yielding genotypes and hybrids are necessary to meet the fodder demand in these regions. To start with breeding material from ICAR- Indian Institute of Millets Research (ICAR-IIMR) was used. The breeding material included male sterile lines and restorer lines. This marks the initiation of forage sorghum breeding in NE India. The success of sorghum hybrid breeding relies on the complementary interaction of the selected parental lines (A, B and R). These lines play a crucial role in the early stages of sorghum hybrid breeding programs, serving as the foundation for developing improved and high-performing cultivars and hybrids. Understanding the stability and adaptability of each parent line allows breeders to optimize combinations that exhibit heterosis, leading to hybrids with superior performance compared to their parents (Kannababu et al., 2017).The sorghum B-lines were specifically bred for fodder purposes, and their adaptability to a wide range of environments will allow breeders to effectively exploit these selected lines in the development of forage sorghum hybrids. The future objectives aim for the implementation of shuttle breeding with stable lines on the Indian subcontinent, where forage sorghum is currently in its infancy. Dominance and overdominance variance influence hybrid performance. It is more important to select stable and high-yielding lines as parents for crosses than to use the whole set of A-lines and test the hybrids that are made in multi-environmental trials (METs).

Forage yield is a complex trait that is influenced by many factors, including the genotype of the plant, the environment in which it is grown, and management practices. Genotype-by-environment interaction (GEI) can make it difficult to identify superior genotypes and can lead to reduced genetic gain in breeding programs. This results in reduced genetic gain and slows down the breeding process (Quintero et al., 2018). Prior to introduction of the crop in non-conventional areas it must be tested for its performance and stability. Also, it is important to prioritise multi-environment trials (METs) in order to examine the stability and pattern of GEI across environments for effective crop improvement programmes (Yan and Kang, 2002; Vaezi et al., 2019). It is crucial to evaluate the performance of selected genotypes based on their adaptability to the local environment. The stability of traits and high heritability contribute to the selection of breeding material for improvement. Stability is one of the important criteria in assessing suitability of genotypes to a region. Several analytical models and techniques, including analysis of variance (ANOVA), regression analysis viz. Finlay and Wilkinson (1963) and Eberhart and Russel (1966); non-parametric methods like, principal component analysis (PCA), additive main effects and multiplicative interaction (AMMI), and genotype and genotype plus environment (GGE) bi-plots, were developed in order to understand the unpredictable effects of genotype, environment, and their interaction (Kendal, 2016; Singamsetti et al., 2023). In light of India’s complex climate, breeding climate-resistant and region-specific high-yielding hybrids has emerged as a top priority for sorghum breeders. The increase in yield may be attributed to the selection of grain yield along with favourable expression of secondary traits. Identifying high-performing genotypes or treatments across multiple traits has been a difficult task. Optimising genotype selection based on grain yield and other relevant agronomic traits could enhance efficiency. Incorporating multiple trait data sets without encountering multi-collinearity in the selection process has posed a challenge for breeders. Therefore multi trait based stability evaluation methods are played crucial role in precise selection of genotypes. The Smith-Hazel index, a commonly used selection index for multiple traits, is not recommended for use in METs due to biased index coefficients and multi-collinearity issues (Smith, 1936; Hazel, 1943; Singamsetti et al., 2023). Breeders have suggested several improved novel multi-trait based stability evaluation methods, including genotype - ideotype distance index (MGIDI), multi-trait stability index (MTSI), multi-trait stability and mean performance (MTMPS) and multi-trait index based on factor analysis and genotype-ideotype distance (FAI-BLUP Index), to address these issues (Rocha et al., 2018; Olivoto et al., 2019b; Zuffo et al., 2020; Olivoto and Nardino, 2021). These methods are devised to aid breeders in maintaining sustainable progress in primary traits, such as forage yield, while preserving genetic advancements in secondary traits. These genotypes enable plant breeders to suggest stable and high-yielding, reliable forage genotypes for specific regions.

In the present study, breeding material consisted of B- lines and R lines were evaluated for their performance and stability. The data was analysed for variability, heritability, inter-trait associations, and the GEI effect. Considering the four multi-trait models, a comparative study is also made to find the most suitable multi-trait stability model and stable genotypes.

## 2 Materials and methods

### 2.1 Plant material

The experimental material for the present investigation comprised 95 genotypes, which included B (Maintainer) lines, R (Restorer) lines and Varieties collected from Indian Institute of Millet Research, Hyderabad (**Supplementary Table 1**). All the B-lines belong to *bicolor* sps and the restorer included Sudan – Sorghum hybrid type.

### 2.2 Testing environment

**Figure 1:**
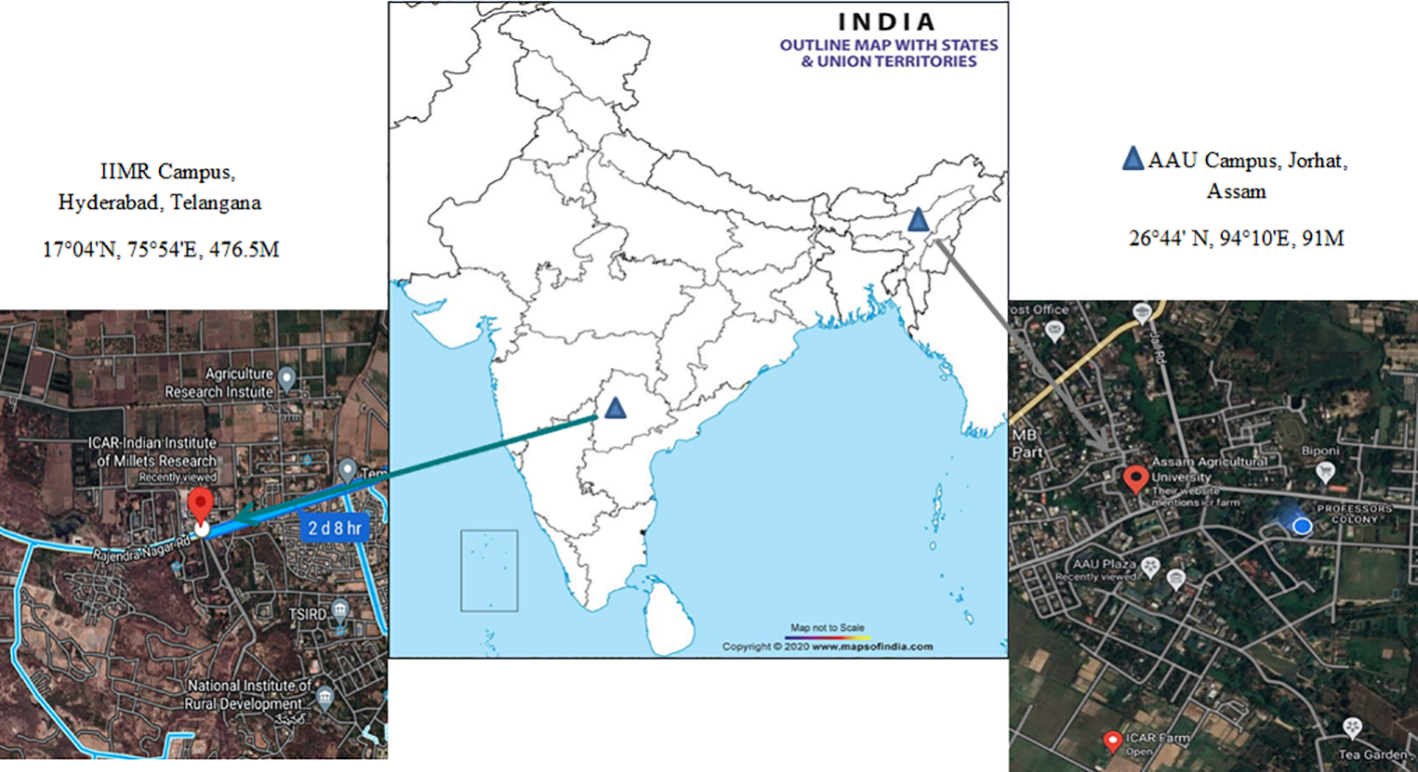
Geographical information of the locations for the testing of 95 forage sorghum genotypes.

The trials were carried out across the different growing season at two locations, viz., Instructional-cum-Research (ICR) Farm, Assam Agricultural University, Jorhat (Location 1) and Breeding Research field, Indian Institute of Millet Research (IIMR), Rajendra Nagar, Hyderabad (Location 2) (**Figure 1**). The first site is in North – Eastern regions of India (26°44’ N, 94°10’ E, elevation of 91masl). The region is characterized as hilly, high rainfall, humid region with mild winters (average mean temperature of 17 degree Celsius). The weather conditions during the evaluation period from October 2020 to January 2022 were almost normal and favourable for crop growth. The second site is located at Southern part of India (17°04’ N, 75°54’ E, elevation of 476.5 MASL) and it is a semi-arid region. The five environments were as follows 1) Rabi season, 2020, Research field, IIMR, Hyderabad (E1); 2) Rabi season, 2020, ICR Farm, AAU, Jorhat (E2); 3) Summer season, 2021, ICR Farm, AAU, Jorhat (E3); 4) Kharif season, 2021, ICR Farm, AAU, Jorhat (E4); 5) Rabi season, 2021, ICR Farm, AAU, Jorhat (E5). The in-depth description of five test environments (E1 to E5) including three growing seasons with location combinations was shown in **Table 1**. The meteorological data based on standard weeks including mean temperature (maximum & minimum), mean relative humidity (morning & evening), total rainfall and bright sunshine hour during the crop growing period at both locations were shown in **Supplementary Figure 1**.

**Table 1:** Characterization of the growing environment based on location, growing season, and meteorological parameters.

Sl No	Particulars	E1	E2	E3	E4	E5
1	Environment Name	Rabi season, 2020	Rabi season, 2020	Summer Season, 2021	Kharif Season, 2021	Rabi Season, 2021
2	Location	Research Field, IIMR, Hyderabad	ICR Farm, AAU, Jorhat	ICR Farm, AAU, Jorhat	ICR Farm, AAU, Jorhat	ICR Farm, AAU, Jorhat
3	Lattitude/Longitude/Altitude	17°04’N, 75°54’E, 476.5M	26°44’ N, 94°10’E, 91M	26°44’ N, 94°10’ E, 91M	26°44’ N, 94°10’ E, 91M	26°44’ N,94°10’E, 91M
4	Soil Texture	Red clay & Red gravelly loam	Sandy loam	Sandy loam	Sandy loam	Sandy loam
5	Date of Sowing	October, 2020	14-10-2020	12-02-2021	25-05-2021	11-10-2021
6	Mean Maximum Temperature (°C)	31.00	27.10	29.99	32.71	27.97
7	Mean Minimum Temperature (°C)	18.33	15.08	17.33	24.85	14.21
8	Mean Relative Humidity (%) (Morning)	98.50	97.84	93.16	93.39	97.03
9	Mean Relative Humidity (%) (Evening)	30.67	64.60	56.63	75.05	59.85
10	Total Rainfall (mm)	27.70	13.23	16.35	50.27	8.91
11	BSSH (hr/day)	7.81	5.58	4.93	3.99	6.73

### 2.3 Experimental design

Ninety-five genotypes were investigated at five different environments at ICR farm, AAU, Jorhat and Research field, IIMR, Hyderabad during the year 2020 and 2021. The experiments layout was randomized complete block design (RCBD) with two replications for all trials under study. In each environment, 95 genotypes are investigated in five different trials and each containing 19 genotypes. Each trial is treated as block. Manual hand sowing was done in two rows of 3 m length with a standard spacing of 45 cm between the rows and 20 cm between plants. All the recommended agronomic and cultural operations including irrigation and plant protection measures were taken care of.

### 2.4 Morphological traits evaluated

A total of fourteen morphological traits were recorded during flowering, maturity and post - harvest stage according to the guidelines for DUS, 2007 of protection of plant varieties and farmer’s rights authority (PPV & FRA), Government of India, New Delhi. The mean value of days to 50% flowering (FDF, in days) was recorded on a plot basis and rest of the other forage yield attributing adaptive traits viz., plant height at 50% flowering (PH, in cm), number of leaves per plant (NLP), leaf length (LFL, in cm), leaf width (LFW, in cm), leaf area index (LAI), leaf to stem ratio (LSR), stem girth (SGT, in mm), number of nodes per plant (NNP), inter-nodal distance (IL, in cm), panicle length (PL, in cm), dry matter content (DMC, in %), dry fodder yield per plant (DFYP, in g) and green fodder yield per plant (GFYP, in g) were made on 5 randomly chosen competitive plants of each genotype in each environment during 2020-2021.

### 2.5 Data analysis and software

#### 2.5.1 Variance component analysis

The best linear unbiased prediction (BLUP) based mixed linear model predicts random effects and genotype mean values more precisely than the other existing stability models by considering genotype, genotypic and interaction effects (GEI) as random and environment and replication effect were considered as fixed effect (Olivoto et al., 2019a). The gamem_met () function from the metan package (Olivoto and Lúcio, 2020) was used to calculate the standard linear mixed model described by Yang (2007).


y=Xβ+Zu+ε

where **y** is a vector of response variable (like grain yield), *β* is a vector of fixed effects, u is a vector of random effects vector, X and Z are design matrices of 0s and 1s relating *β* and u to y, respectively, and *ϵ* is a vector of random errors. A two-tailed chi-square test with one degree of freedom was conducted using a likelihood ratio test (LRT) to assess the statistical significance of the random effects. The genetic parameters estimated in this multi-environmental trial (METs) are calculated by using the methodologies as given below by (Olivoto et al., 2019a; Olivoto and Lúcio, 2020).

Heritability is the broad-sense heritability, $h_{g}^{2}$ , calculated by


$$h_{g}^{2}=\frac{\sigma_{ɡ}^{2}}{\sigma_{ɡ}^{2}+\sigma_{i}^{2}+\sigma_{e}^{2}}$$

The symbols $\sigma_{ɡ}^{2}$ , $\sigma_{i}^{2}$ , and $\sigma_{e}^{2}$ signify variance due to genotypes, genotype-by-environment interaction, and residual term, respectively.

Heritability of means is the heritability on the mean basis, $h_{gm}^{2}$ , estimated by


$$h_{gm}^{2}=\frac{\sigma_{ɡ}^{2}}{\sigma_{ɡ}^{2}+\sigma_{i}^{2}/e+\sigma_{e}^{2}/eb}$$

Where e and b are the number of environments and blocks, respectively.

Accuracy is the accuracy of selection, Ac, calculated by


$$AC=\sqrt{h_{ɡm}^{2}}$$

GEIr^2^ is the coefficient of determination of the interaction effects, $r_{i}^{2}$ , estimated by


$$r_{i}^{2}=\frac{\sigma_{i}^{2}}{\sigma_{ɡ}^{2}+\sigma_{i}^{2}+\sigma_{e}^{2}}$$

r_ge_ is the genotype-environment correlation, estimated by


$$r_{=\frac{\sigma_{ɡ}^{2}}{\sigma_{ɡ}^{2}+\sigma_{i}^{2}}}$$

CVg and CVr are the genotypic coefficient of variation and the residual coefficient of variation estimated, respectively, by


$$CVg=\left(\sqrt{\frac{\sigma_{g}^{2}}{\mu }}\right)x100$$

$$CVr=\left(\sqrt{\frac{\sigma_{g}^{2}}{\mu }}\right)x100$$

Where μ is the grand mean.

#### 2.5.2 Multi – trait based stability evaluation methods

These multi-trait-based stability methods were used to identify the ideotype or ideal type genotypes, considering all the studied traits. In the present investigation, only days to 50% flowering was desired for a lower mean value, and the rest of the forage yield traits were desired for a higher mean value.

##### 2.5.2.1 Multi-trait genotype-ideotype distance index

The MGIDI was computed using the methodology of Olivoto and Nardino (2021) in four steps, which is as follows: rescaling the traits, factor analysis, ideotype designing (the ideotype exhibits a rescaled value of 100 for all evaluated traits) and calculating the MGIDI index.


$$MGIDI_{i}=\sqrt{\left(\sum_{j=1}^{f}{\left(Y_{ij}-Y_{j}\right)}^{2}\right)}$$

Where, *MGIDI_i_* is the multi-trait genotype-ideotype distance index for *i*th genotype, *γ_ij_* is the score of the *i*th genotype in the *j*th factor (*i = 1, 2,., t*; *j = 1, 2,., f*), being *t* and *f* the number of genotypes and factors and *γ_j_* is the *j*th score of ideotype.

The strength and weakness of genotypes were assessed by calculating the proportion of the MGIDI of the *i*th genotype explained by the *j*th factor (*ω_ij_)* as follows.


$$\omega_{ij}=\frac{\sqrt{D_{ij}^{2}}}{\sum_{j=1}^{j}\sqrt{D_{ij}^{2}}}$$

Where, *D_ij_* is the distance between the *i*th genotype and ideal genotype for the *j*th factor. A trait with low contribution indicates that the genotypes within such trait are close to ideal genotype.

##### 2.5.2.2 The multi-trait stability index

The multi-trait stability index (MTSI) was derived through factor analysis. The MTSI differs from the MGIDI in that it incorporates WAASBY (mean performance and stability) values in the Fgp matrix through factor analysis, whereas MGIDI incorporates only BLUP mean (mean performance). The genotype ranking was determined by calculating the Euclidean distance using the scores of each genotype compared to the score of the ideotype. The multi-trait stability index (MTSI) (Olivoto et al., 2019b) was calculated by following formula.


$$MTSI_{i}=\sqrt{\left(\sum_{j=1}^{f}{\left(F_{ij}-F_{j}\right)}^{2}\right)}$$

Where, the *MTSIi* is the multi-trait stability index for the *i*th genotype, *F_ij_* = *j*th score of the *i*th genotype; *F_j_* = *j*th score of the ideotype.

MTSI utilises the harmony between average performance and stability to identify genotypes that exhibit both high performance and stability. Assigning all trait weights in the MTSI to average performance results in the transformation of the MTSI into the MGIDI index. MGIDI ranks genotypes based on multiple traits but does not account for genotype stability.

The multi-trait mean performances and stability index (MTMPS) is derived from the multi-trait stability index, MTSI, as proposed by Olivoto et al. (2019b) and Zuffo et al. (2020). In this study, the MTMPS was calculated using Wricke’s Ecovalence (Wi) instead of the WAASB index, which is the only difference from the MTSI (Olivoto and Nardino, 2021).

##### 2.5.2.3 Multi-trait index based on factor analysis and genotype-ideotype distance (FAI-BLUP Index)

Once the ideotype is established, calculate the spatial probability of each genotype by estimating its distance from the ideotype. This ranking will aid in the evaluation of the genotypes. The calculation formula of Multi-Trait Index Based on Factor Analysis and Genotype-Ideotype Distance (FAI-BLUP Index) is as follows:


$$P_{ij}=\left[\frac{\left(\frac{1}{d_{ij}}\right)}{\sum_{i=1;j=1}^{i=n;j=m}\left(\frac{1}{d_{ij}}\right)}\right]$$

Where *P_ij_* represents the probability that the *i*th genotype (*i = 1, 2,…, n*) is similar to the *j*th genotype *(j = 1, 2,…, m*); *d_ij_* represents the genotype-ideotype distance from the *i*th genotype to the *j*th ideotype according to the standardized average Euclidean distance (Rocha et al., 2018).

##### 2.5.2.4 Selection differential and genetic gain under selection

The genotypes were selected under different growing environments through MGIDI values by considering a selection intensity of ~15% and the selection differential in the percentage of population mean (Δ*S*%) was then computed for each trait as follows


$$\Delta S\%=\frac{\left(X_{s}-X_{o}\right)}{X_{o}}X100$$

Genetic gain percentage under selection (Δ*G*%)is calculated as follows


$$\Delta G\%=\frac{\left(X_{s}-X_{o}\right)}{X_{o}}XhX100$$

Where, Xo = mean for WAASBY index of the original population; Xs = mean for WAASBY index of the selected genotypes; ΔS: Selection differential; ΔG: Selection gains

### 2.6 Software used for statistical analysis

All analyses were done in R studio (Posit team, 2022) environment with R version 4.1.2 (R Core Team, 2021). Stability analysis for different models with different parameters was analysed using “metan” (Olivoto and Lúcio, 2020) and “ggplot2” version 3.3.4 (Wickham, 2016) packages. The MGIDI index is calculated using the mgidi() function. The mtsi() function is used for calculating the multi-trait stability index (MTSI), as suggested by Olivoto et al. (2019) in metan. The FAI-BLUP is a multi-trait index that utilises factor analysis and ideotype design. It can be calculated using the fai_blup function, as described by Rocha et al. (2018). The coincidence_indexes) function in metan can calculate the coincidence index as defined by Hamblin and Zimmermann (1986), along with other multi-trait indexes.

## 3 Results

### 3.1 Mean performances

Mean performances of 95 forage sorghum genotypes in five environments for 14 adaptive or yield contributing traits are presented in the **Table 2**, **Supplementary Table 2**. The highest and lowest values for green forage yield per plant (g) were observed in the genotypes G63 (447.51 g) and G67 (102.5 g), respectively, with an average of 260.59 g. The top four genotypes with green forage yield were G35, G69, G36 and G58; 46 genotypes were above overall mean. The mean performance in general was high and the highest in E2 environment, whereas E5 general mean was the lowest. The lowest and highest values for days to 50% flowering were observed in the genotypes G67 (49.5 days) and G56 (91.5 days), respectively, with an average of 72.62 days. The lower value of days to 50% flowering is preferable in crop breeding. The most desirable early flowering genotypes were G44, G17, G45 and G18 and 47 genotypes out of 95 had a desired mean over the grand mean.

**Table 2:** Mean performance of 95 forage sorghum genotypes for 14 adaptive traits in five environments (Summary).

Particulars	FDF	PH	NLP	LFL	LFW	LAI	SGT
Min	46 (G67 in E5)	72.55 (G94 in E3)	5.2 (G20 in E5)	43.12 (G77 in E3)	2.12 (G67 in E3)	0.62 (G67 in E3)	6.51 (G17 in E4)
Max	98 (G82 in E2)	279.4 (G17 in E1)	13.82 (G21 in E1)	102.79 (G17 in E5)	9.8 (G62 in E2)	7.82 (G64 in E5)	28.91 (G26 in E3)
Min ENV	E5 (69.91)	E4 (134.15)	E5 (9.27)	E4 (60.19)	E3 (6.61)	E3 (3.38)	E3 (16.22)
Max ENV	E2 (76.79)	E2 (150.74)	E2 (10.32)	E5 (65.37)	E2 (7.38)	E2 (4.19)	E2 (17.9)
Min GEN	G67 (49.5)	G94 (93.63)	G67 (7.05)	G42 (48.52)	G67 (3.11)	G67 (1.03)	G67 (8.42)
Max GEN	G56 (91.5)	G17 (248.05)	G21 (11.84)	G64 (78.65)	G21 (8.46)	G64 (6.25)	G26 (23.44)
Mean	72.62	139.85	9.88	62.5	6.99	3.66	16.87
SEm	1.032	4.097	0.206	1.046	0.196	0.197	0.612
No of Gen above Mean	47	37	50	51	52	51	50
Particulars	LSR	NNP	IL	PL	DMC	DFYP	GFYP
Min	0.08 (G69 in E4)	2.85 (G59 in E1)	3.37 (G52 in E4)	12.81 (G46 in E4)	15 (G57 in E3)	18.93 (G27 in E3)	65.91 (G17 in E4)
Max	0.34 (G61 in E4)	9 (G63 in E2)	37.76 (G17 in E2)	42.15 (G17 in E5)	35.92 (G75 in E4)	189.93 (G69 in E2)	545.43 (G69 in E1)
Min ENV	E4 (0.2)	E1 (4.72)	E5 (11.58)	E3 (23.85)	E4 (27.93)	E5 (69.52)	E5 (243.34)
Max ENV	E2 (0.22)	E2 (5.56)	E2 (13.27)	E2 (26.3)	E2 (29.53)	E2 (82.06)	E2 (276.45)
Min GEN	G44 (0.1)	G26 (3.52)	G52 (5.21)	G46 (16.2)	G47 (19.86)	G67 (30.93)	G67 (102.5)
Max GEN	G26 (0.29)	G63 (8.42)	G17 (30.94)	G17 (39.01)	G86 (33.03)	G69 (139.84)	G63 (447.51)
Mean	0.21	5	12.05	24.77	28.7	74.99	260.59
SEm	0.007	0.122	0.368	0.588	0.475	2.616	9.402
No of Gen above Mean	47	42	37	46	49	40	46

[days to 50% flowering (FDF, in days), plant height at 50% flowering (PH, in cm), number of leaves per plant (NLP), leaf length (LFL, in cm), leaf width (LFW, in cm), leaf area index (LAI), leaf to stem ratio (LSR), stem girth (SGT, in mm), number of nodes per plant (NNP), inter-nodal distance (IL, in cm), panicle length (PL, in cm), dry matter content (DMC, in %), dry fodder yield per plant (DFYP, in g) and green fodder yield per plant (GFYP, in g).

### 3.2 Combined analysis of variance for adaptive traits over five environments

The mean squares from the combined analysis of variance for forage yield and its attributed traits are presented in **Table 3**. The results of the combined analysis of variance for different traits showed that mean squares due to genotypes, environments and genotype x environment interactions (GEI) are highly significant.

**Table 3:** Mean squares from combined analysis of variance for forage yield and its attributing traits of 95 forage sorghum genotypes across five environments.

Source	Df	FDF	PH	NLP	LFL	LFW	LAI	SGT	LSR	NNP	IL	PL	DMC	DFYP	GFYP
ENV	4	1229.91**	9566.86**	32.49**	1227.56**	14.30	19.21	99.04	0.018**	24.86**	93.14**	212.38**	63.95**	5802.26**	41863.68**
REP(ENV)	5	9.19	113.75	0.81	4.96	0.32	0.51	3.4	0.0003	0.02	0.97	2.45	4.34**	78.87	956.00
BLOCK (REP*ENV)	20	9.16*	199.54**	0.26	12.68*	0.33*	0.22	4.14	0.00048	0.23*	1.53	5.55**	3.04**	86.69	880.37
GEN	94	340.96**	7664.53**	8.84**	386.73**	8.59**	6.86**	76.22	0.0235**	8.21**	234.57**	123.18**	62.68**	6326.95**	65154.05**
GEN : ENV	376	22.65**	515.89**	2.04**	51.75**	1.04**	0.95**	5.48	0.0008**	0.58**	7.01**	6.80**	10.50**	437.92**	4529.82**
Residuals	450	5.43	97.12	0.37	7.82	0.20	0.27	2.37	0.0003	0.13	1.00	2.34	2.19	67.27	589.47
CV (%)		3.21	7.05	6.12	4.47	6.42	14.11	9.12	8.76	7.22	8.32	6.18	5.16	10.94	9.32

**p < 0.01 and *p < 0.05.

[days to 50% flowering (FDF, in days), plant height at 50% flowering (PH, in cm), number of leaves per plant (NLP), leaf length (LFL, in cm), leaf width (LFW, in cm), leaf area index (LAI), leaf to stem ratio (LSR), stem girth (SGT, in mm), number of nodes per plant (NNP), inter-nodal distance (IL, in cm), panicle length (PL, in cm), dry matter content (DMC, in %), dry fodder yield per plant (DFYP, in g) and green fodder yield per plant (GFYP, in g)]

### 3.3 BLUP based genetic parameter analysis

The BLUP based variance components for all 14 traits in five environments is presented in **Table 4**. The genotype and genotype x environment interactions had highly significant effects (P < 0.001) on the sorghum forage yield and contributing traits according to the likelihood ratio tested against the *Chi*-square value. The BLUP based heritability was high for inter-nodal length (85%) and lowest for number of leaves per plant (36.2%), however, mean based heritability showed 97% and 77% respectively for the same two traits.

**Table 4:** Likelihood ratio test and genetic parameters analysis of forage sorghum genotypes for forage yield and its attributes.

Parameters	LRT_g_	LRT_ge_	σ^2^p	Heritability (%)	GEIr2 (R^2^ge)	h^2^mg (%)	Selection Accuracy (%)	r_ge_	CV_g_	CV_r_	CV_g_/CV_r_
FDF	374***	199***	45.9	69.3	0.186	93.4	96.6	0.604	7.77	3.25	2.39
PH	370***	264***	1.02E+03	69.8	0.202	93.3	96.6	0.671	19.1	7.20	2.65
NLP	103***	296***	1.88	36.2	0.446	77.0	87.7	0.699	8.35	6.08	1.37
LFL	201***	340***	63.4	52.8	0.345	86.6	93.1	0.731	9.26	4.53	2.04
LFW	223***	261***	1.38	54.7	0.303	87.9	93.7	0.669	12.4	6.51	1.91
LAI	194***	168***	1.20	49.4	0.286	86.2	92.8	0.565	21	14.1	1.50
SGT	352***	68.4***	11	64.1	0.138	92.8	96.3	0.383	15.8	9.27	1.70
LSR	554***	92.9***	0.00286	79.3	0.0911	96.4	98.2	0.44	23.1	8.84	2.61
NNP	358***	214***	1.12	68.2	0.198	93.0	96.4	0.622	17.5	7.33	2.38
IL	617***	359***	26.8	85.0	0.112	97.0	98.5	0.745	39.6	8.41	4.71
PL	426***	106***	16.3	71.5	0.133	94.5	97.2	0.466	13.8	6.36	2.17
DMC	156***	242***	11.6	45.0	0.357	83.2	91.2	0.65	7.96	5.20	1.53
DFYP	363***	339***	842	69.9	0.22	93.1	96.5	0.731	32.4	11	2.94
GFYP	361***	393***	8630.000	70.3	0.228	93.0	96.5	0.765	29.9	9.41	3.17

*** significant at P < 0.001, LRT significance test is conducted against the Chi-square value.

Where, (LRT - The Likelihood Ratio Test for the random effects; Phenotypic variance the phenotypic variance; Heritability the broad-sense heritability BLUP basis; ${\text{R}}_{\text{ge}}^{2}$ the coefficient of determination of the interaction effects; ${\text{h}}_{\text{mg}}^{2}$ the heritability on the mean basis; Accuracy the selective accuracy; rge the genotype-environment correlation; CV_g_ the genotypic coefficient of variation; CVr the residual coefficient of variation; CV ratio the ratio between genotypic and residual coefficient of variation).

So, the selection accuracy (AS) of 95 forage sorghum genotypes was the lowest for number of leaves per plant and the highest for inter-nodal length and its high values for all the traits. The coefficient of determination of the interaction effects ( ${\text{R}}_{\text{ge}}^{2}$ ) was the lowest for leaf to stem ratio and the highest for number of leaves per plant and low values were observed in almost all the traits. The genotypic correlation among environments (r_ge_) was high for green forage yield per plant, inter-nodal length, dry forage yield per plant, leaf length, number of leaves per plant, plant height, leaf width, dry matter content, number of nodes per plant, days to 50% flowering and leaf area index.

### 3.4 Loadings and factor description for MGIDI

The eigenvalues, explained variance, factorial loadings after varimax rotation, and communalities obtained in the factor analysis through MGIDI analysis of 14 adaptive traits in 95 forage sorghum were presented in **Table 5**. The factors with eigen values >1 were retained in the four factors, which indicated that these four factors explained 76.52% of the total variation present among the attributes based on the WAASBY value of BULP estimates. After varimax rotation, the average communality (h) was 0.76, and the maximum and minimum values recorded by 0.91 in plant height and 0.51 in days to 50% flowering. These studied traits were divided into four factors. Number of leaves per plant, leaf width, leaf area index, stem girth, dry forage yield per plant and green forage yield per plant; belonged to FA1. FA2 included traits such as plant height, leaf length, number of nodes per plant and inter-nodal length, whereas, FA3 included the trait, panicle length. The remaining three traits, days to 50%, leaf to stem ratio and dry matter content, were in FA4.

**Table 5:** Loadings and factor description through MGIDI analysis in 14 adaptive traits in 95 forage sorghum.

Trait	FA1	FA2	FA3	FA4	Communality
FDF	0.395	-0.353	0.193	0.435	0.51
PH	0.004	-0.919	0.193	0.167	0.91
NLP	-0.622	0.074	-0.490	-0.256	0.70
LFL	-0.466	-0.568	0.448	-0.133	0.76
LFW	-0.854	0.150	0.109	0.020	0.76
LAI	-0.921	-0.151	0.047	-0.120	0.89
IL	0.223	-0.833	0.197	0.163	0.81
SGT	-0.698	0.382	0.045	0.061	0.64
LSR	0.087	0.397	0.052	-0.781	0.78
PL	-0.042	-0.095	0.886	0.013	0.80
NNP	-0.179	-0.791	-0.217	0.272	0.78
DMC	-0.033	-0.011	0.053	0.811	0.66
DFYP	-0.727	-0.357	-0.089	0.486	0.90
GFYP	-0.758	-0.406	-0.107	0.276	0.83
Eigenvalues	4.447	3.645	1.475	1.146	
Variance (%)	31.761	26.039	10.538	8.183	
Cum. variance (%)	31.761	57.800	68.338	76.521	

### 3.5 Genetic correlations among the forage yield and its contributing traits

**Figure 2:**
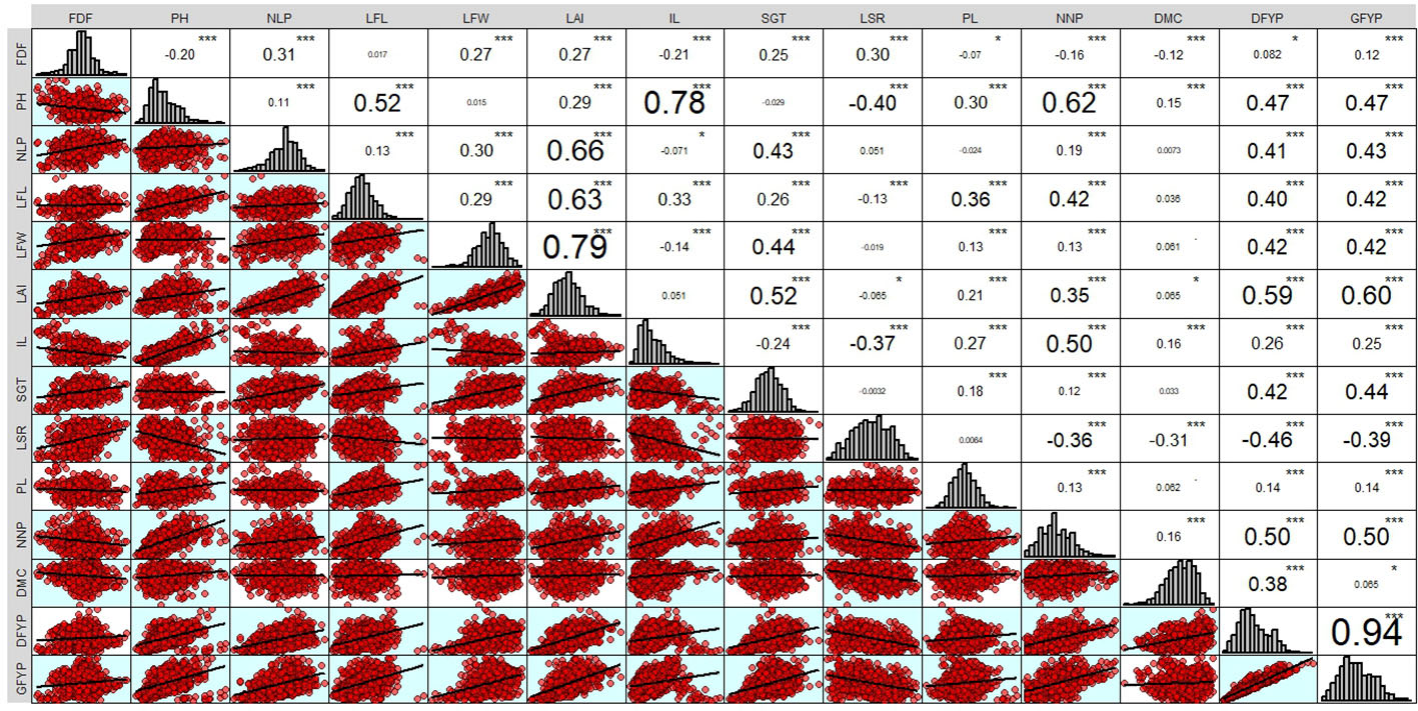
Genetic correlations among the sorghum forage yield and its attributing traits. * = p < 0.05 and *** = p <0.001.

The genotypic correlation of 95 forage sorghum genotypes for 14 adaptive traits is presented in **Figure 2**. In the present study, the green forage yield per plant was dependent trait and the remaining thirteen yield contributing traits were independent (causal) traits. In general, genotypic correlation is usually greater than phenotypic correlation. Green forage yield per plant (g) recorded a significant and positive association with dry forage yield per plant, leaf area index, plant height, number of nodes per plant, leaf width, leaf length, stem girth and number of leaves per plant.

### 3.6 Selection of genotypes based on MGIDI analysis

**Figure 3:**
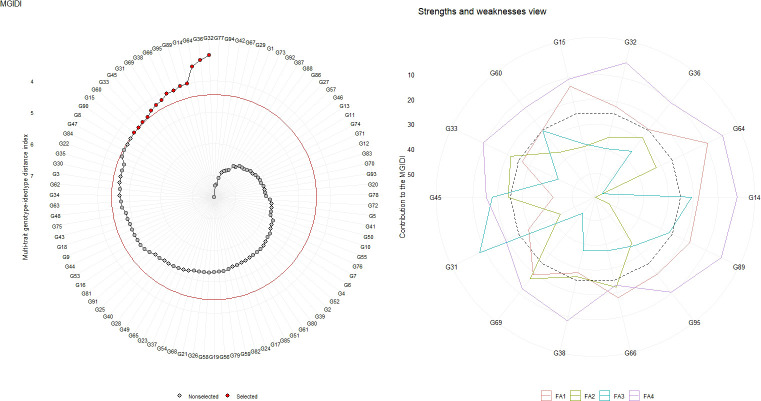
Genotype ranking of selected genotypes based on MGIDI scores, and the strengths and weaknesses view of the selected genotypes is shown as the proportion of each factor considering a selection intensity of 15%.

Out of 95 forage sorghum genotypes, 14 were selected based on 15% selection intensity. The genotype ranking, determined by the MGIDI score, is presented in **Figure 3**, **Supplementary Table 3**. The genotypes G32, G36, G64, G14, G89, G95, G66, G38, G69, G31, G45, G33, G60, and G15 were selected according to their MGIDI scores.

### 3.7 Predicted genetic gains under selection

The predicted genetic gains under selection and selection differential for all the traits were presented in **Table 6**. The MGIDI Index achieved a success rate of 78.57% in selecting desired traits based on BLUP matrix, with a desired selection differential (SD) for 11 out of 14 traits. Positive selection differentials were observed in all traits except for three traits i.e. days to 50% flowering (-0.96), stem girth (-0.40), and leaf to stem ratio (-0.03), which had undesired negative selection differentials. The WAASBY index had a mean selection differential of 11.12%, with the leaf to stem ratio having the lowest (-13.16%) and the dry forage yield per plant having the highest (32.51%). The mean genetic gain under selection (SG%) was 8.83%, being the lowest one (-11.54%) for the leaf to stem ratio and the highest one (26.81%) for the dry forage yield per plant.

**Table 6:** Selection gain for mean performance across the environments based on the MGIDI values.

Trait	Factor	Xo	Xs	SD	SD (%)	h2	SG	SG (%)	Indicator
NLP	FA1	9.88	9.97	0.09	0.89	0.55	0.05	0.49	increase
LFW	FA1	6.99	7.51	0.52	7.47	0.71	0.37	5.32	increase
LAI	FA1	3.66	4.37	0.71	19.53	0.65	0.47	12.78	increase
SGT	FA1	16.87	16.46	-0.40	-2.39	0.77	-0.31	-1.85	increase
DFYP	FA1	74.99	99.37	24.38	32.51	0.82	20.11	26.81	increase
GFYP	FA1	260.59	336.04	75.45	28.95	0.83	62.80	24.10	increase
PH	FA2	139.85	165.02	25.17	18.00	0.82	20.57	14.71	increase
LFL	FA2	62.50	69.03	6.54	10.46	0.68	4.46	7.13	increase
IL	FA2	12.05	15.55	3.50	29.09	0.92	3.22	26.72	increase
NNP	FA2	5.00	5.70	0.69	13.84	0.78	0.54	10.76	increase
PL	FA3	24.77	27.02	2.25	9.08	0.81	1.83	7.39	increase
FDF	FA4	72.62	71.66	-0.96	-1.33	0.77	-0.75	-1.03	decrease
LSR	FA4	0.21	0.18	-0.03	-13.16	0.88	-0.02	-11.54	increase
DMC	FA4	28.70	29.49	0.79	2.74	0.65	0.51	1.77	increase
Mean =				9.91	11.12		8.13	8.83	
Total SD perc (Increase) = 157.00						Total SG perc (Increase) = 124.60			
Total SD perc (decrease) = -1.33						Total SG perc (decrease) = -1.03			

Xo = mean for WAASBY index of the original population; Xs = mean for WAASBY index of the selected genotypes; SD and SD perc, The selection differential and selection differential in percentage, respectively; SG and SG perc, The selection gains and selection gains in percentage, respectively

### 3.8 The strengths and weaknesses view

**Figure 4:**
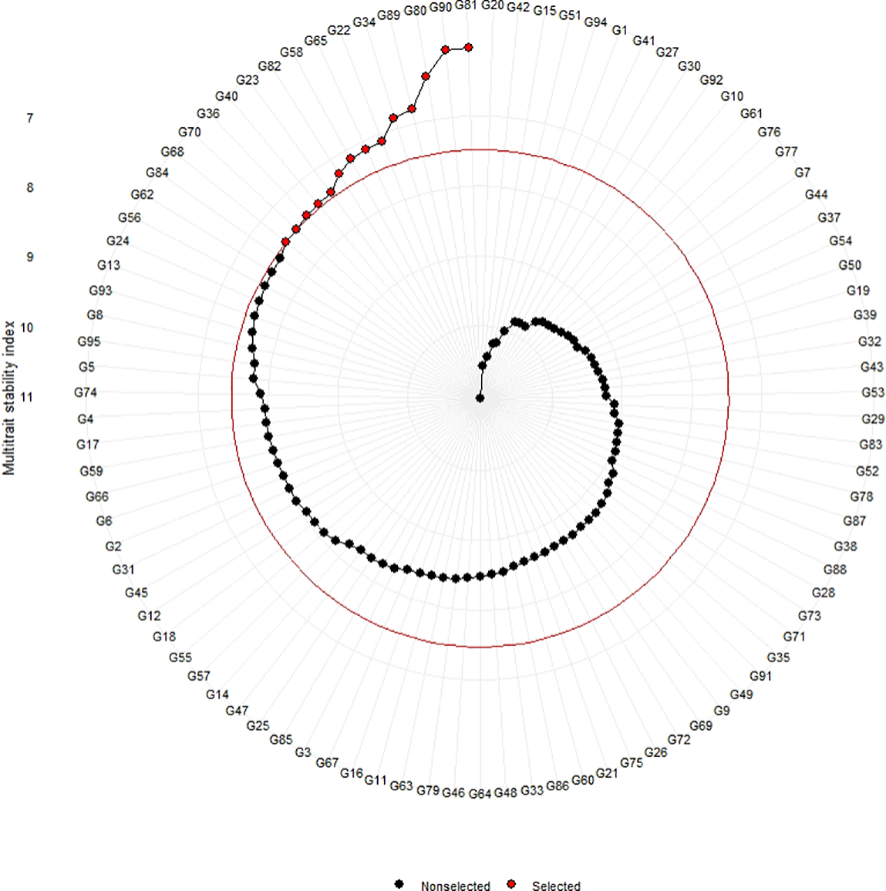
Genotype ranking of selected genotypes based on MTSI scores considering a selection intensity of 15%.

The contribution of each component to the MGIDI was divided into two categories: less and more contributing factors. The more significant contributing factors were displayed towards the centre, while the less significant contributing factors were placed towards the edge. The strength and weakness view of selected genotypes out of 95 forage sorghum genotypes based on MGIDI score was presented in the **Figure 4** and supported by the **Supplementary Table 4**. In the case of FA1 (number of leaves per plant, leaf width, leaf area index, stem girth, dry forage yield per plant and green forage yield per plant), most of the selected genotypes were weak contributors except G45 and G38. In the case of FA2 (plant height, leaf length, number of nodes per plant and inter-nodal length), genotypes G69, G66, G33 and G45 were weak contributors to MGIDI compared to the remaining traits. Most of the selected genotypes were weak contributor for the traits grouped in FA1. As their comparatively strong contributions indicated, these genotypes were desirable and closer to the ideotype except G45 and G38 for these traits. These genotypes, G69, G66, G33 and G45 were weak contributors, stable and closer to an ideotype. They were selected and involved in breeding programmes for the improvement of these traits included in FA2. In the case of FA3 (panicle length), G31 and G14 were weak contributors to MGIDI as compared to the remaining traits that indicated genotypes were stable and closer to the ideotype. In FA4 (trait days to 50%, leaf to stem ratio and dry matter content), all the genotypes were weak contributors, while MGIDI indicated genotypes were stable and nearer to the ideotype for the above mentioned trait.

### 3.9 Coincidence, common genotypes selected from the different multi-trait based stability models

**Figure 5:**
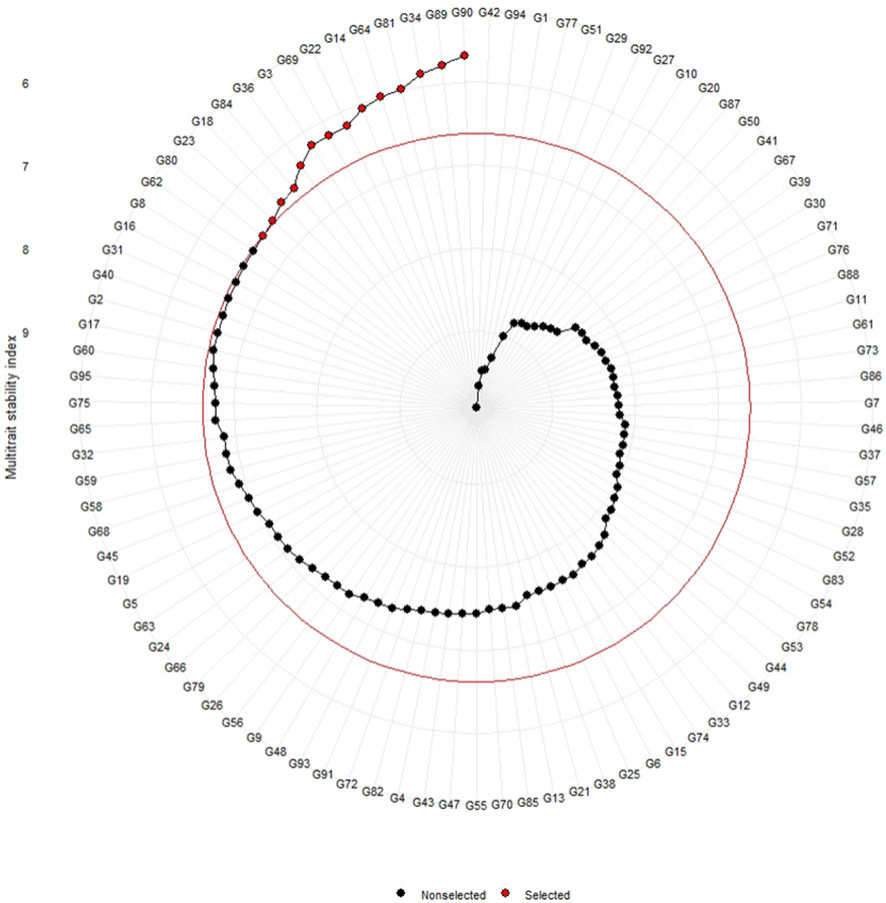
Genotype ranking of selected genotypes based on MTMPS scores considering a selection intensity of 15%.

**Figure 6:**
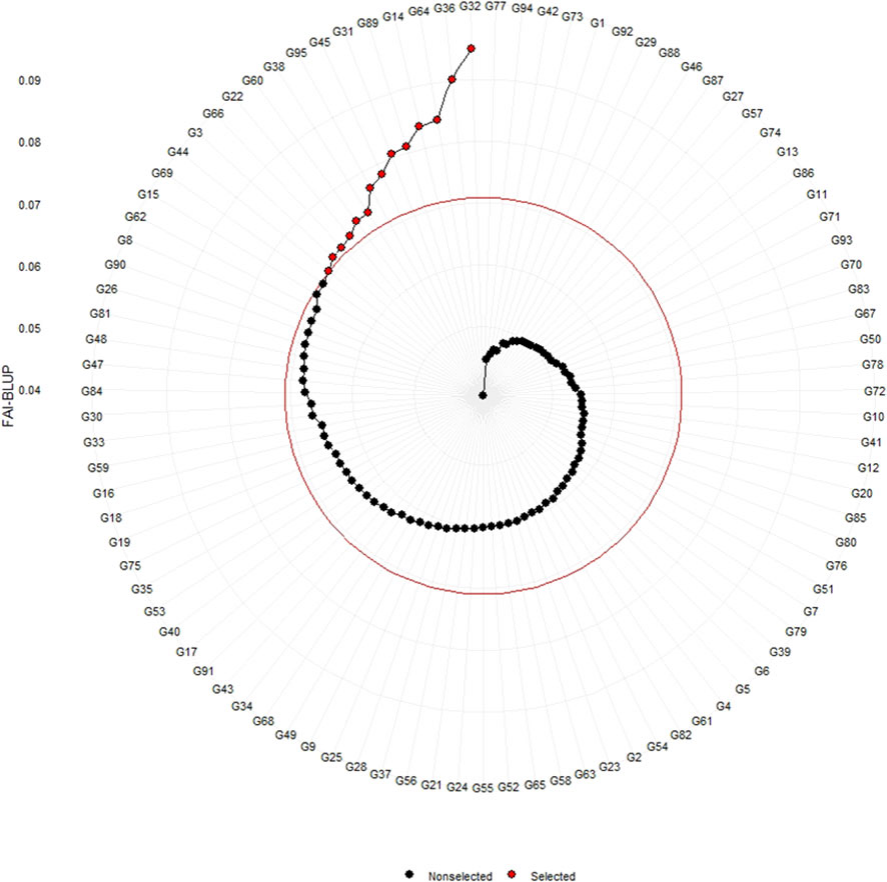
Genotype ranking of selected genotypes based on FAI – BLUP scores considering a selection intensity of 15%.

**Figure 7:**
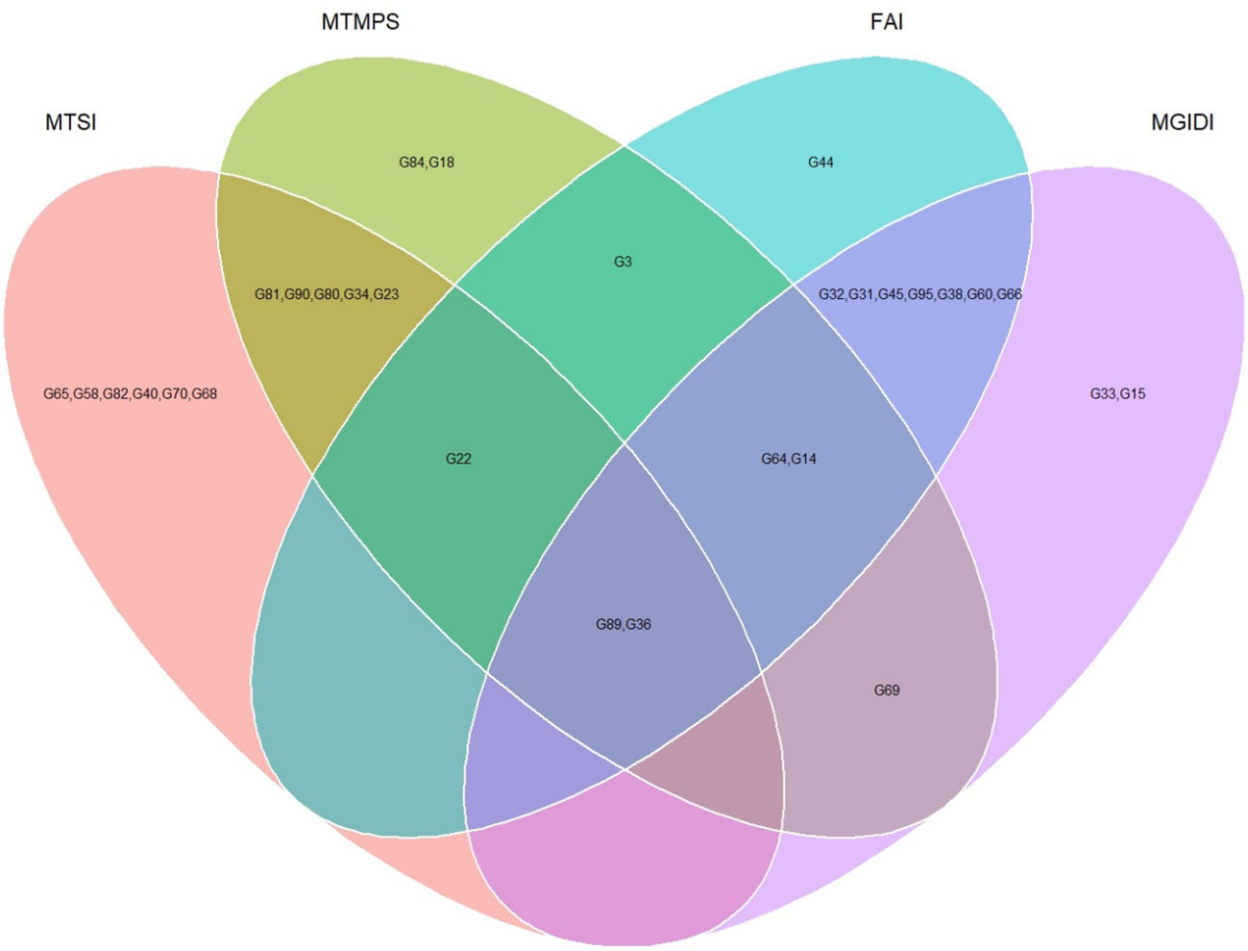
Venn chart of common genotypes and number of genotypes selected across the different multi trait stability analysis considering a selection intensity of 15%.

The genotypes were selected based on the different multi trait stability index, considering the selection intensity of 15% as presented in **Table 7**; **Supplementary Table 3**. The genotypes G32, G36, G64, G14, G89, G95, G66, G38, G69, G31, G45, G33, G60 and G15 based on MGIDI (**Figure 3**). The genotypes selected according to the multi trait stability index (MTSI) were G81, G90, G80, G89, G34, G22, G65, G58, G82, G23, G40, G36, G70 and G68 (**Figure 4**). The multi-trait mean performance and stability index (MTMPS) identified genotypes G90, G89, G34, G81, G64, G14, G22, G69, G3, G36, G84, G18, G23 and G80 (**Figure 5**). The genotypes selected according to the multi trait index based on factor analysis and ideotype-design (FAI BLUP) were G32, G36, G64, G14, G89, G31, G45, G95, G38, G60, G22, G66, G3 and G44 (**Figure 6**). Comparing the different multi-trait based stability models; the coincidence index was higher between the two models that have more common genotypes between them. The common genotypes and coincidence index were presented in **Table 8** and the Venn chart in **Figure 7**. The maximum coincidence index with common genotypes was observed between MGIDI and FAI-BLUP (74.79%, 11), respectively, followed by MTSI and MTMPS (49.57%, 8), FAI – BLUP and MTMPS (32.77%, 6), MGIDI and MTMPS (24.37%, 5), MTSI and FAI – BLUP (7.56%, 3) and the least value was observed between MGIDI and MTSI (-0.84%, 2). The common genotypes observed across all models were G36 (302B) and G89 (348B).

**Table 7:** Selection of genotypes based on different multi trait stability model.

MTSI Model	Selected Genotypes
MGIDI	G32, G36, G64, G14, G89, G95, G66, G38, G69, G31, G45, G33, G60, G15
MTSI	G81, G90, G80, G89, G34, G22, G65, G58, G82, G23, G40, G36, G70, G68
MTMPS	G90, G89, G34, G81, G64, G14, G22, G69, G3, G36, G84, G18, G23,G80
FAI-BLUP	G32, G36, G64, G14, G89, G31, G45, G95, G38, G60, G22, G66, G3, G44

**Table 8:** Common genotypes between the multi trait stability models based on coincidence index.

Variable-1	Variable-2	Coincidence index	common	genotypes	Common genotype across all models
MGIDI	MTSI	-0.84	2	G36,G89	G36,G89
MGIDI	FAI – BLUP	74.79	11	G32,G36,G64,G14,G89,G95,G66,G38,G31,G45,G60	
MGIDI	MTMPS	24.37	5	G64,G89,G69, G36, G14	
MTSI	FAI – BLUP	7.56	3	G89,G22,G36	
MTSI	MTMPS	49.57	8	G90,G89,G22,G36, G23, G34, G80, G81	
FAI – BLUP	MTMPS	32.77	6	G22,G36,G64,G89,G3,G14	

## 4 Discussions

In this present investigation, the performance of 95 Sorghum forage genotypes were evaluated for 14 agronomic traits in two consecutive planting years (2020–2021) under the climatic conditions of Jorhat of Assam and Indian institute of millet research (IIMR) of Rajendra Nagar, Hyderabad. To our knowledge, this is the first report of employing WAASB and multi – trait stability model in understanding Genotype × Environment interactions and stability analysis in Sorghum forage hybrids. Developing high-yielding fodder hybrids is one strategy that could be used to address the massive global shortage of green and dry fodder. A significant hindrance to the progress of forage sorghum hybrid creation has been the scarcity of female parents (male-sterile), maintainer, and restorer lines that have been particularly designated or customized for the purpose of forage sorghum hybrid development. Identifying promising parents and subsequently producing a commercial hybrid from them is a formidable undertaking (Patel et al., 2019). The evaluations of stability and adaptability of these lines are in accordance with the principles of sustainable agriculture. They aim to foster the growth of crop varieties that are less susceptible to failure, contribute to long-term environmental and economic sustainability, and assist in tackling the difficulties posed by fluctuating and evolving environmental conditions (Rao et al., 2011). The work is primarily focused on minimizing the duration of breeding, specifically addressing the environment, lowering resource usage, and supplying the appropriate genotypes for hybrid development.

Genotype-by-environment interactions (GEI) can significantly impede selection efficiency and hinder the development of adapted varieties, particularly for complex traits like forage yield (Rao et al., 2011). Accuracy of prediction models aid in interpretation of multi-environment trials (Gauch and Zobel, 1988). GEI studies with linear mixed models are more efficient in predicting genotypic responses compared to fixed effect models like AMMI family models (van Eeuwijk et al., 2016). Mixed models can be used to estimate various biometrical parameters, including broad-sense variance, heritability estimates, and genetic correlation among traits (Olivoto et al., 2019a). Olivoto et al. (2019b) proposes to use mean performance and stability of multiple desirable agronomic traits to improve varietal recommendations. The ideotype is postulated to possess a maximum WAASBY score of 100 for all the considered traits. The genotype with the lowest multi trait based selection index is selected as it is closer to the ideotype. In this present investigation, simultaneous selection for forage yield and early maturity has been carried out through four important multi trait based stability evaluation methods including MGIDI, MTSI, MTMPS and FAI – BLUP and a comparative study in the selection of ideal genotypes had done. Among the four methods, MGIDI had given more weightage in this current study.

Evaluating the agronomic performance of germplasm is crucial for selecting stably performing genotypes across locations with desirable traits. Such selected genotypes can be constituted to from a core-subset for trait improvement. In the present study lines with high value for multiple traits were identified as superior viz. G44 (309B), G69 (352B), G17 (CSV33MF), G45 (373B), G90 (424B) and G63 (442B) showed desirable mean performance for multiple traits along with high forage yield. The genotypes, G63, G38, G62, G64, G32, G75 and G44 were the desirable genotypes with early maturity with high yield and these genotypes could be included in development of high yielding varieties breeding programmes.

The results of pooled ANOVA showed that there is significant genotype, environment, and genotype x environment interactions effects for most of the traits suggested that the genotypes were suitable for estimating the GEI and stability parameters. Similarly, Patel et al. (2019) found significant differences in genotypes and G x E for days to flowering, plant height, number of leaves per plant, stem diameter, and yield of green and dry fodder per plant in both the individual and pooled environments. Likewise, these kinds of results were also reported by Chala et al. (2019) and Nagesh Kumar et al. (2021).

The BLUP model, which incorporates genotypic and interaction effects (GEI) as random, is superior to other models in predicting random effects and genotype mean values with greater precision. Genetic variability is crucial for enhancing agronomic traits in plant breeding selection procedures. Heritability knowledge aids plant breeders in selecting an appropriate method for enhancing a trait, estimating the selection’s benefit, and assessing the genetic effects’ significance. The study found statistically significant genotypic and GEI effects for all recorded traits (p ≤ 0.001). This showed that genotype mean performances differed among growing conditions, which could increase genotype diversity and provide sufficient variation for easy selection (Yue et al., 2022). So, a higher heritability of a trait indicates a higher effectiveness of selection with high selection accuracy. The high selection accuracy of these traits illustrated the model’s reliability in the selection of better genotypes. The low values of R^2^_ge_ were observed in almost all the traits indicating considerable residual variance in the G x E interaction component, unlike the AMMI ANOVA, which explained most of it via the first two IPCAs and the GEI variance contribute less towards the phenotypic variance component (Olivoto et al., 2019a; Yue et al., 2022).

The high genotypic correlation among environments (r_ge_) was recorded in most of the traits indicated a consistent trend across environments and easier identification of stable and superior genotypes while it was low for other remaining traits, suggesting that selecting stable and superior genotypes for these attributes is challenging and requires detailed, reliable information. BLUP based genetic parameters were comparatively smaller than mean based genetic parameters, with the same trend. Similar results were reported by Sousa et al. (2019) in cowpea for immature seed production, Koundinya et al. (2021) in 25 cassava genotypes and Yue et al. (2022) in 28 maize genotypes. The four factors (FA1 to FA4) with eigenvalues greater than one accounted for a significant proportion of the total variation. Therefore, it was possible to maintain strong explanatory strength while reducing the dimensionality of data. The maximum value of communality indicating that a significant portion of the variance of each trait might be explained by these factors (FA). All the studied traits were grouped in 4 factors based on their communality value. Similar kinds of results were also reported in barley for identification of salt-tolerant barley genotypes using multiple-traits index and yield performance at the early growth and maturity stages by Pour-Aboughadareh et al. (2021); Yue et al. (2022) in maize hybrids; Olivoto et al. (2019b) in oat; Olivoto and Nardino (2021) in wheat and Debsharma et al. (2023) in rice.

Yield is a complex trait that arises from the interplay of various yield-contributing factors that exhibit either positive or negative correlations with yield as well as with each other. The correlation coefficient is useful in identifying beneficial indicators of high yield by evaluating the relative influence of various characters on yield and among themselves during selection. Characters that had notable positive or negative correlations with green fodder yield were prioritised during the selection process for achieving a high green forage yield per plant. Arvinth et al. (2021) also reported similar results, where Green fodder yield per plant have positive and significant association with number of leaves per plant, stem girth, leaf length of blade, leaf width of blade and dry fodder yield per plant. These results were also supported by several breeders (Santhiya et al., 2021; Thant et al., 2021; Patil et al., 2022 and Chauhan and Pandey, 2021).

Selected genotypes were used to calculate selection differentials. Genotype G15, with an MGIDI score of 4.44, exhibited the cut point at the final red circle, accounting for selection intensity. Genotype G90, located in close proximity to the circle, may possess significant traits. Therefore, further investigation is required for the genotypes at the cut point. Positive selection differences were noted in adaptive traits except for leaf-to-stem ratio, stem girth, and days to 50% flowering. This suggests that these selected genotypes were more stable, as per the WAASBY index for these traits. A negative selection differential (%) and genetic gain under selection (%) were advantageous due to the lower desired value of the trait, such as days to 50% flowering. Genotypes selected for dry forage yield per plant, inter-nodal length, green forage yield per plant, and plant height based on the WAASBY index exhibited high heritability and genetic gain, indicating their stability and desirability for these traits. The aforementioned traits could be improved by directly selecting and incorporating these genotypes into the breeding programme. Olivoto and Nardino (2021) reported similar results in wheat, while Yue et al. (2022) found comparable outcomes in maize hybrids.

The strength and weakness of traits could be judged by the factor analysis viz., the contribution of each component to the MGIDI. Traits that are closer to the ideotype tend to have lower proportions, explained by the factor, which is located near the exterior edge of the figure. All genotypes, except G45 and G38, exhibited weak contribution and stability and were closer to the ideotype for various plant characteristics such as number of leaves, leaf width, leaf area index, stem girth, dry forage yield, and green forage yield. Genotypes G69, G66, G33, and G45 exhibited greater stability in terms of plant height, leaf length, number of nodes per plant, and inter-nodal length. G31 and G14 exhibited greater stability with respect to panicle length. All genotypes exhibited stability and proximity to the ideotype for days to 50% flowering. So, strength and weakness plot is a potential graphical tool to identify and select genotype based on their strength and weakness for the respective traits can be improved. Similar kinds of results were reported by Olivoto et al. (2021) in strawberry, Benakanahalli et al. (2021) in Guar, Mamun et al. (2022) in EMS induced rice mutant and Debsharma et al. (2023) in rice.

The application of a multi-trait-based stability method would enable the selection of genotypes based on their stability across multiple traits and mean performance. The method relies on genotype-ideotype distance and factor analysis scores. The ideotype exhibits the maximum WAASBY values across all the observed traits (Olivoto et al., 2019b). Multi-trait-based stability methods’ lower values indicate stable genotypes across multiple traits.

The genotypes G36 and G89 were consistently observed in all models and were considered highly desirable and reliable. Consequently, these genotypes were incorporated into the ideotype-based breeding programmes. The two genotypes exhibited ideal mean performances based on MGIDI selection and stability as per the MTSI selection criteria. Genotype G90 was found to be close to the cut point in the MGIDI and was detected in the models MTSI and MTMPS. On the other hand, genotype G89 was detected in all models and exhibited a high green forage yield per plant. These genotypes were considered ideal for both forage yield and ideotype design in the ideotype breeding pipeline. Yue et al. (2022) reported comparable findings in Maize hybrids, while Olivoto and Nardino (2021) and Benakanahalli et al. (2021) observed similar outcomes in Wheat and Guar, respectively. When MET data is used for genetic analysis and genomic selection, noise is produced and phenotypic values are misinterpreted, leading to wrong inferences. Multi-trait stability analysis could speed up the process of curation of phenotypic data by identifying stable genotypes in a varied panel across contexts. The correlation between the stability of parents and hybrids has not been demonstrated. However, quality parameters are more susceptible to being impacted by multi-environment factors compared to parameters relating to forage production.

The current study based on four techniques to identify genotypes that consistently perform well across many traits. may introduce bias towards specific traits or combinations of traits. This could lead to the exclusion of potentially valuable genotypes that perform well in certain environments or for specific traits not emphasized in the selection process. Since the current study was with limited number of environments, a better picture could have been possible with the use of more diverse environmental conditions. A long-term assessment of genotype performance and stability over multiple years may provide more robust insights into their suitability for climate-resilient fodder sorghum production. The performance of the genotypes is determined by both the direct measurement of the characteristic and the influence of associated traits. The influence of the associated characteristics on the economic characteristic helps in choosing genotypes in populations that remain consistent across different environments, which also serves as an indirect indicator of heritability across different locations. Further research is warranted to validate the effectiveness of the identified genotypes, especially under field conditions and across a wider range of environments. Additionally, exploring additional traits relevant to climate resilience and abiotic stress tolerance could enhance breeding efforts for developing climate-resilient forage sorghum genotypes.

## 5 Conclusion

The use of the BLUP based multi –trait stability techniques in multi-environment trials facilitated an accurate depiction of genotype-environment interaction (GEI). Four distinct methods for evaluating genotype ranking, namely MGIDI, MTSI, MTMPS, and FAI-BLUP, have been employed. The multi-trait-based stability evaluation methods facilitated the selection of stable genotypes, with positive selection differentials for traits that wanted to increase and negative selection differentials for one trait that wanted to decrease. This means that breeders and agronomists that aim at simultaneous selection for mean performance and stability while considering various attributes might find these strategies effective. It provides a unique selection process, including weights for each trait that are easy to interpret, and considers the correlation structure among the traits, which will make the index more applicable and provide better treatment recommendations. The study aims to enhance specific traits in specific environments. Overall, this study opens the door to the use of these tools to analyse plant multivariate data, standing out as a powerful tool to develop better recommendation strategies in sorghum forage. The results showed that genotypes G36 (302B) and G89 (348B) were found to be common across all four evaluation methods based on all the studied traits. The aforementioned genotypes were found to be the most reliable, high-yielding, and early-maturing and could be suggested for variety development and ideotype breeding programmes to ensure the food and nutritional security of an ever increasing population.

## Data availability statement

The raw data supporting the conclusions of this article will be made available by the authors, without undue reservation.

## Author contributions

PB, RN and AS designed the research and performed the experiments. BB, AS and VR contributed experiment materials to perform field experiment and provide critical support during laboratory based analysis. RS, JB and PM provide support during data analysis. PB, HV, LG, NB and NS wrote the manuscript with help from all the other authors. All authors contributed to the article and approved the submitted version.
