# Supplementary figures and images for: Genetic gains in forage sorghum for adaptive traits for non - conventional area through multi-trait-based stability selection methods
Source: Front Plant Sci. 2024 Mar 7;15:1248663. doi: 10.3389/fpls.2024.1248663 (PMC10961980; doi:10.3389/fpls.2024.1248663)

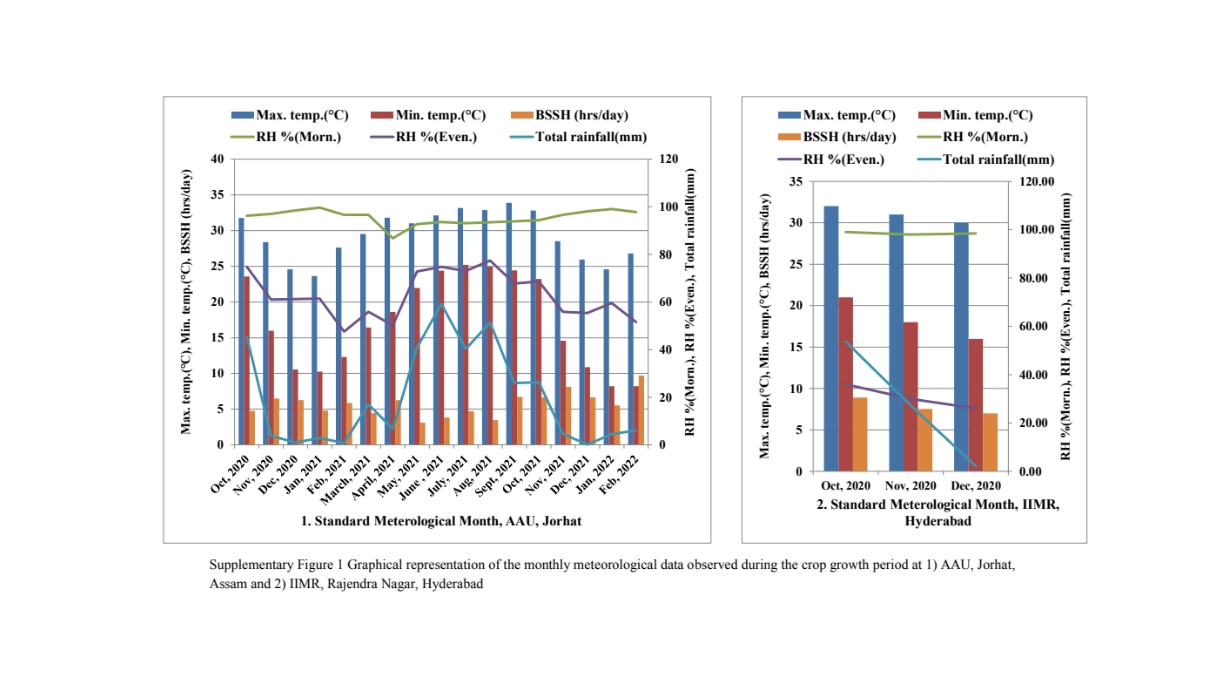

Supplement: Supplementary file 1 [file Image_1.jpeg]
