# Supplementary material for: Genetic gains in forage sorghum for adaptive traits for non - conventional area through multi-trait-based stability selection methods
Source: Front Plant Sci. 2024 Mar 7;15:1248663. doi: 10.3389/fpls.2024.1248663 (PMC10961980; doi:10.3389/fpls.2024.1248663)
Supplement: Supplementary file 2 [file Table_1.docx]

Supplementary Table 1. List of genotypes included in the present study (Source - BVB, IIMR, Hyderabad)

| Genotype Code | Sample code | Genotype Name | Type | Pedigree Information |
| --- | --- | --- | --- | --- |
| G1 | Sample 1 _ 20 | 403B | B Line | (NSSB 1003 X NSSB 26)-3-1 |
| G2 | Sample 10 _ 20 | 415B | B Line | (NSSB 1003 x ICSB 342)-1-3-1 |
| G3 | Sample 11 _ 20 | 349B | B Line | (ICSB 342 x ICSB 467)-3-1-3 |
| G4 | Sample 13 _ 19 | 321B | B Line | (NSSB 1002 X 296B)-7-1 |
| G5 | Sample 14 _ 20 | 445B | B Line | 44121B x NSS 20B-5 |
| G6 | Sample 122 _ 20 | 370B (whIte) | B Line | (90001B x NSSB 1005)-4-1-2 |
| G7 | Sample 17 _ 20 | 353B | B Line | (ICSB 342 x ICSB 467)-10-1-1 |
| G8 | Sample 18 _ 20 | 336B | B Line | (NSSB 1002 x NSSB 1005)-3-5-3 |
| G9 | Sample 19 _ 20 | 301B | B Line | (NSSB 2 X 2219B)-3-1 |
| G10 | Sample 20 _ 19 | 412B | B Line | (NSSB 1002 x NSSB 1005)-3-5-3 |
| G11 | Sample 21 _ 19 | 354B | B Line | (ICSB 342 x ICSB 687)-1-3-2 |
| G12 | Sample 2 _ 20 | ICS56B | B Line | (Serere elite x IS 9530)-2 |
| G13 | Sample 23 _ 20 | 429B | B Line | (ICSB 342 x ICSB 687)-1-3-2 |
| G14 | Sample 24 _ 20 | NSS11B | B Line | Pedigree not available |
| G15 | Sample 26 _ 20 | 402B | B Line | (NSSB 1003 X NSSB 26)-2-2 |
| G16 | Sample 123 _ 20 | 428B | B Line | (ICSB 342 x ICSB 467)-10-2-2 |
| G17 | Sample 119 _ 20 | CSV33MF | Variety | EMS mutant of CO FS 29 |
| G18 | Sample 29 _ 20 | 384B | B Line | (NSSB 5 X 2219B)-5-1 |
| G19 | Sample 30 _ 20 | 308B | B Line | (NSSB 5 X 2219B)-4-2 |
| G20 | Sample 31 _ 20 | 370B(Black) | B Line | (90001B x NSSB 1005)-4-1-2 |
| G21 | Sample 32 _ 20 | 323B | B Line | (NSSB 1002 X 296B)-12-1 |
| G22 | Sample 33 _ 20 | NSS23B | B Line | Pedigree not available |
| G23 | Sample 3 _ 20 | 334B | B Line | (27B X NSSB 1002)-2-3 |
| G24 | Sample 34 _ 20 | 413B | B Line | (NSSB 1002 x NSSB 1005)-5-1-2 |
| G25 | Sample 35 _ 20 | 337B | B Line | (NSSB 1002 x NSSB 1005)-5-1-2 |
| G26 | Sample 36 _ 20 | 345B | B Line | (ICSB 338 x ICSB 342)-6-1-1 |
| G27 | Sample 37 _ 20 | 359B | B Line | (ICSB 687 x ICSB 702)-4-3-3 |
| G28 | Sample 38 _ 20 | 367B | B Line | (ICSB 702 x ICSB 697)-5-3-1 |
| G29 | Sample 39 _ 19 | 2077B | B Line | IS 2046 selection |
| G30 | Sample 40 _ 19 | 388B | B Line | (NSSB 6 X (CSV 17 X PKV 809))-8-2 |
| G31 | Sample 41 _ 20 | NSS10B | B Line | Pedigree not available |
| G32 | Sample 42 _ 20 | 322B | B Line | (NSSB 1002 X 296B)-8-1 |
| G33 | Sample 43 _ 20 | 311B | B Line | (NSSB 6 X (CSV 17 X PKV 809))-4-2 |
| G34 | Sample 4 _ 20 | 389B | B Line | (NSSB 6 X (CSV 17 X PKV 809))-10-2 |
| G35 | Sample 45 _ 19 | NSS1B | B Line | Pedigree not available |
| G36 | Sample 46 _ 20 | 302B | B Line | (NSSB 2 X 2219B)-3-2 |
| G37 | Sample 47 _ 20 | 338B | B Line | (NSSB 1003 x ICSB 342)-1-3-1 |
| G38 | Sample 48 _ 20 | 363B | B Line | (ICSB 687 x ICSB 702)-9-1-3 |
| G39 | Sample 49 _ 20 | 385B | B Line | (NSSB 5 X 2219B)-8-2 |
| G40 | Sample 50 _ 20 | 346B | B Line | (ICSB 338 x ICSB 342)-7-3-1 |

**Supplementary Table 1 Continued….**

| Genotype Code | Sample code | Genotype Name | Type | Pedigree Information |
| --- | --- | --- | --- | --- |
| G41 | Sample 55 _ 20 | NSS1008B | B Line | Pedigree not available |
| G42 | Sample 57 _ 20 | 365B | B Line | (ICSB 686 x NSSB 1)-1-1-1 |
| G43 | Sample 58 _ 20 | 407B | B Line | (27B X NSSB 20)-1-3 |
| G44 | Sample 59 _ 20 | 309B | B Line | (NSSB 5 X 2219B)-5-1 |
| G45 | Sample 5 _ 20 | 373B | B Line | (ICSB 702 x (27BxSSV 84))-10-3-1 |
| G46 | Sample 60 _ 20 | CSV30F | Variety | NSS 223 x NARI 111 |
| G47 | Sample 61 _ 20 | NSS5B | B Line | Pedigree not available |
| G48 | Sample 62 _ 20 | 358B | B Line | (ICSB 687 x ICSB 702)-4-2-1 |
| G49 | Sample 63 _ 20 | 339B | B Line | (NSSB 1003 x ICSB 342)-3-2-3 |
| G50 | Sample 64 _ 20 | 360B | B Line | (ICSB 687 x ICSB 702)-6-1-1 |
| G51 | Sample 65 _ 19 | 436B | B Line | (ICSB 687 x ICSB 702)-4-3-3 |
| G52 | Sample 67 _ 20 | 419B | B Line | (ICSB 338 x ICSB 342)-2-3-2 |
| G53 | Sample 68 _ 19 | 375B | B Line | 44121B x NSS 20B-5 |
| G54 | Sample 69 _ 19 | 410B | B Line | (NSSB 1002 x NSSB 1005)-1-1-3 |
| G55 | Sample 71 _ 20 | 296B | B Line | Karad Local x IS 3922 |
| G56 | Sample 118 _ 20 | CSV27 | Variety | (GJ 38 x Indore 12) - 2 - 1 - 2 - 1 GJ 38 = GJ 35 x E 35 - 1 |
| G57 | Sample 74 _ 19 | 408B | B Line | (27B X NSSB 1002)-2-3 |
| G58 | Sample 75 _ 20 | NSS1003B | B Line | Pedigree not available |
| G59 | Sample 77 _ 19 | 329B | B Line | (NSSB 1003 X NSSB 26)-3-2 |
| G60 | Sample 78 _ 19 | NSS7B | B Line | Pedigree not available |
| G61 | Sample 79 _ 20 | 435B | B Line | (ICSB 687 x ICSB 702)-4-2-1 |
| G62 | Sample 80 _ 20 | ICS27B | B Line | Pedigree not available |
| G63 | Sample 81 _ 20 | 442B | B Line | (90001B x NSSB 1005)-6-3-1 |
| G64 | Sample 96 _ 20 | 2219B | B Line | kafir shallu seletion |
| G65 | Sample 97 _ 20 | 325B | B Line | (NSSB 1002 X NSSB 23)-10-1 |
| G66 | Sample 98 _ 20 | 313B | B Line | (NSSB 6 X (CSV 17 X PKV 809))-10-2 |
| G67 | Sample 7 _ 19 | PC615 | Restorer | Pusa chari 40 × Pusa Chari 67 |
| G68 | Sample 113 _ 20 | 444B | B Line | 44121B x NSS 13B-3 |
| G69 | Sample 101 _ 20 | 352B | B Line | (ICSB 342 x ICSB 467)-9-1-2 |
| G70 | Sample 102 _ 20 | 335B | B Line | (NSSB 13 x ICSB 699)-1-1-1 |
| G71 | Sample 120 _ 20 | NSS1002B | B Line | Pedigree not available |
| G72 | Sample 106 _ 20 | 351B | B Line | (ICSB 342 x ICSB 467)-8-2-1 |
| G73 | Sample 115 _ 20 | CSV-20 | Variety | SPV 946 x Kh 89 -246 |
| G74 | Sample 110 _ 20 | 377B | B Line | 94001B x 41735-1 |
| G75 | Sample 8 _ 20 | 342B | B Line | (ICSB 4 x NSSB 13)-4-1-2 |
| G76 | Sample 9 _ 20 | 330B | B Line | (NSSB 1003 X NSSB 26)-9-1 |
| G77 | sample12 _ 19 | MP CHARI | Restorer | K- 49 x J-57 |
| G78 | sample15 _ 19 | 369B | B Line | (90001B x NSSB 1005)-3-1-1 |
| G79 | sample16 _ 19 | 355B | B Line | (ICSB 683 x NSSB 8)-2-2-3 |
| G80 | sample22 _ 19 | 382B | B Line | (NSSB 2 X 2219B)-3-2 |
| G81 | sample25 _ 19 | RS29 | Restorer | Pedigree not available |
| G82 | sample27 _ 19 | 467B | B Line | [((ICSB 11 × ICSV 700) × PS 19349B) × ICSB 13]4-1 |

**Supplementary Table 1 Continued….**

| Genotype Code | Sample code | Genotype Name | Type | Pedigree Information |
| --- | --- | --- | --- | --- |
| G83 | sample28 _ 19 | Red B | B Line | Pedigree not available |
| G84 | sample44 _ 19 | 327B | B Line | (NSSB 1003 X NSSB 26)-2-2 |
| G85 | sample51 _ 19 | CSV33MF | Variety/ Restorer | Derived from COFS29 |
| G86 | sample52 _ 19 | CSV32F | Variety/ Restorer | HC 260 x B 35 |
| G87 | sample53 _ 19 | CSV21F | Variety/ Restorer | GSSV 148 x SR 897 |
| G88 | sample54 _ 19 | UPMC503 | Restorer | Selection from IS 5977 |
| G89 | sample56 _ 19 | 348B | B Line | (ICSB 342 x ICSB 467)-2-3-3 |
| G90 | sample66 _ 19 | 424B | B Line | (ICSB 342 x ICSB 467)-2-3-3 |
| G91 | sample70 _ 19 | 314B | B Line | (NSSB 15 X 296B)-2-1 |
| G92 | sample72 _ 19 | PCD-8-1-2 | Restorer |  |
| G93 | sample73 _ 19 | 307B | B Line | (NSSB 5 X 2219B)-4-1 |
| G94 | sample76 _ 19 | 409B | B Line | (27B X NSSB 1002)-8-2 |
| G95 | sample 6 _ 19 | SSG-59-3 | Restorer | Non sweet sudan grass × IS-263 |

Supplementary Table 2 Mean performances of 95 sorghum genotypes for 14 adaptive traits in individual environments

| GEN | FDF | | | | | | PH | | | | | | NLP | | | | | |
| --- | --- | --- | --- | --- | --- | --- | --- | --- | --- | --- | --- | --- | --- | --- | --- | --- | --- | --- |
|  | E1 | E2 | E3 | E4 | E5 | AVG | E1 | E2 | E3 | E4 | E5 | AVG | E1 | E2 | E3 | E4 | E5 | AVG |
| G1 | 64.50 | 64.50 | 62.50 | 66.50 | 70.50 | 65.70 | 113.25 | 107.20 | 104.90 | 113.84 | 153.75 | 118.59 | 10.01 | 10.50 | 6.80 | 9.15 | 9.20 | 9.13 |
| G2 | 73.00 | 73.50 | 73.00 | 72.50 | 67.00 | 71.80 | 124.31 | 133.15 | 105.75 | 107.92 | 112.25 | 116.67 | 9.51 | 8.80 | 8.90 | 8.50 | 6.50 | 8.44 |
| G3 | 56.50 | 77.50 | 64.50 | 76.00 | 71.50 | 69.20 | 131.67 | 149.81 | 123.09 | 125.85 | 130.26 | 132.13 | 10.72 | 10.85 | 7.30 | 10.80 | 9.20 | 9.77 |
| G4 | 76.50 | 82.50 | 71.00 | 80.50 | 77.50 | 77.60 | 113.21 | 166.60 | 101.68 | 109.15 | 123.25 | 122.78 | 10.59 | 10.95 | 8.85 | 10.35 | 9.60 | 10.07 |
| G5 | 75.50 | 79.00 | 75.00 | 75.00 | 69.00 | 74.70 | 116.52 | 145.23 | 98.93 | 101.47 | 106.65 | 113.76 | 8.58 | 9.00 | 8.70 | 8.55 | 6.90 | 8.35 |
| G6 | 77.00 | 81.00 | 74.00 | 76.00 | 69.00 | 75.40 | 124.70 | 163.46 | 102.12 | 110.89 | 111.06 | 122.45 | 10.44 | 10.60 | 7.20 | 10.70 | 9.60 | 9.71 |
| G7 | 79.50 | 81.50 | 72.00 | 79.50 | 67.00 | 75.90 | 116.84 | 133.46 | 164.04 | 111.17 | 113.17 | 127.74 | 11.86 | 11.30 | 11.50 | 10.50 | 9.70 | 10.97 |
| G8 | 72.50 | 75.00 | 69.50 | 73.00 | 72.00 | 72.40 | 129.89 | 168.81 | 116.75 | 150.80 | 127.18 | 138.68 | 11.39 | 10.40 | 9.40 | 10.95 | 9.60 | 10.35 |
| G9 | 67.00 | 75.50 | 68.00 | 74.00 | 70.00 | 70.90 | 163.00 | 180.47 | 134.47 | 151.00 | 194.66 | 164.72 | 10.90 | 11.00 | 9.60 | 10.50 | 10.70 | 10.54 |
| G10 | 79.00 | 78.00 | 73.00 | 62.00 | 70.00 | 72.40 | 130.20 | 146.85 | 115.14 | 130.68 | 104.61 | 125.49 | 10.63 | 10.65 | 9.30 | 10.90 | 7.20 | 9.74 |
| G11 | 74.50 | 79.50 | 67.50 | 74.00 | 70.50 | 73.20 | 133.10 | 133.69 | 117.54 | 119.30 | 111.69 | 123.06 | 11.14 | 12.20 | 10.50 | 10.90 | 9.20 | 10.79 |
| G12 | 63.50 | 72.00 | 64.50 | 66.50 | 63.00 | 65.90 | 116.15 | 119.85 | 110.97 | 114.24 | 104.11 | 113.06 | 9.37 | 10.05 | 9.21 | 9.62 | 6.40 | 8.93 |
| G13 | 78.50 | 80.50 | 74.50 | 77.00 | 70.00 | 76.10 | 129.13 | 145.22 | 112.35 | 115.49 | 106.96 | 121.83 | 10.40 | 10.70 | 10.20 | 11.10 | 7.80 | 10.04 |
| G14 | 73.50 | 79.00 | 74.50 | 74.50 | 71.00 | 74.50 | 158.14 | 177.52 | 131.10 | 140.17 | 108.15 | 143.02 | 10.27 | 10.90 | 10.10 | 10.70 | 9.20 | 10.23 |
| G15 | 59.00 | 77.50 | 72.50 | 72.00 | 68.00 | 69.80 | 165.75 | 147.90 | 171.57 | 178.30 | 125.23 | 157.75 | 11.54 | 12.20 | 11.00 | 11.85 | 7.90 | 10.90 |
| G16 | 78.00 | 81.50 | 77.50 | 78.50 | 74.00 | 77.90 | 133.85 | 145.37 | 127.27 | 140.78 | 133.90 | 136.23 | 10.20 | 10.80 | 9.50 | 10.60 | 9.60 | 10.14 |
| G17 | 55.00 | 60.50 | 54.50 | 57.00 | 59.00 | 57.20 | 274.10 | 248.85 | 239.49 | 218.82 | 258.97 | 248.05 | 11.03 | 7.50 | 6.80 | 7.60 | 7.40 | 8.07 |
| G18 | 65.00 | 68.50 | 65.50 | 65.00 | 59.50 | 64.70 | 151.85 | 169.01 | 143.75 | 151.19 | 210.40 | 165.24 | 7.79 | 8.70 | 7.20 | 7.95 | 9.10 | 8.15 |
| G19 | 68.00 | 73.50 | 66.50 | 67.50 | 63.50 | 67.80 | 144.09 | 127.95 | 141.78 | 117.95 | 146.93 | 135.74 | 8.39 | 8.00 | 7.40 | 7.20 | 8.40 | 7.88 |
| G20 | 77.00 | 78.00 | 67.00 | 72.50 | 71.50 | 73.20 | 148.50 | 145.40 | 145.23 | 126.91 | 115.21 | 136.25 | 7.99 | 7.10 | 8.50 | 6.40 | 5.60 | 7.12 |
| G21 | 83.00 | 83.50 | 79.50 | 79.50 | 78.00 | 80.70 | 135.57 | 163.20 | 105.45 | 128.05 | 115.98 | 129.65 | 13.41 | 12.50 | 10.40 | 12.40 | 10.50 | 11.84 |
| G22 | 71.00 | 74.00 | 68.50 | 73.00 | 67.50 | 70.80 | 180.45 | 178.25 | 128.42 | 162.12 | 144.74 | 158.80 | 9.44 | 9.50 | 8.65 | 9.40 | 8.70 | 9.14 |
| G23 | 66.50 | 70.50 | 62.50 | 68.50 | 69.50 | 67.50 | 130.84 | 136.02 | 120.36 | 120.21 | 127.27 | 126.94 | 11.14 | 11.50 | 11.90 | 6.80 | 10.50 | 10.37 |
| G24 | 76.50 | 79.00 | 75.50 | 75.00 | 69.00 | 75.00 | 123.60 | 135.37 | 112.45 | 108.15 | 102.86 | 116.49 | 10.49 | 10.70 | 11.03 | 10.00 | 7.70 | 9.98 |
| G25 | 78.00 | 82.00 | 74.50 | 77.00 | 71.50 | 76.60 | 151.80 | 146.85 | 113.65 | 147.16 | 109.19 | 133.73 | 11.41 | 10.35 | 10.10 | 10.20 | 8.45 | 10.10 |
| G26 | 85.00 | 89.00 | 77.00 | 80.50 | 84.00 | 83.10 | 105.50 | 115.29 | 119.59 | 94.51 | 90.50 | 105.08 | 10.30 | 10.15 | 9.25 | 8.25 | 8.75 | 9.34 |
| G27 | 78.00 | 79.50 | 76.00 | 75.00 | 68.00 | 75.30 | 108.35 | 109.01 | 92.92 | 98.82 | 123.38 | 106.50 | 8.55 | 9.30 | 7.00 | 9.50 | 10.15 | 8.90 |
| G28 | 78.00 | 77.50 | 71.00 | 74.00 | 72.00 | 74.50 | 119.25 | 128.33 | 143.28 | 121.86 | 115.34 | 125.61 | 10.88 | 11.60 | 10.81 | 11.90 | 7.90 | 10.62 |
| G29 | 64.50 | 79.00 | 75.00 | 72.00 | 70.50 | 72.20 | 92.25 | 104.01 | 118.02 | 88.47 | 100.58 | 100.66 | 10.02 | 10.22 | 11.30 | 9.35 | 10.00 | 10.18 |
| G30 | 74.00 | 78.00 | 73.50 | 74.50 | 69.50 | 73.90 | 175.95 | 154.48 | 167.25 | 174.71 | 100.73 | 154.62 | 11.04 | 10.75 | 9.77 | 11.05 | 10.05 | 10.53 |
| G31 | 75.00 | 77.50 | 68.50 | 73.50 | 72.00 | 73.30 | 134.65 | 158.37 | 139.83 | 144.98 | 152.89 | 146.14 | 9.07 | 10.30 | 9.20 | 7.33 | 9.75 | 9.13 |
| G32 | 57.00 | 75.50 | 76.00 | 72.00 | 68.00 | 69.70 | 177.70 | 183.86 | 147.56 | 179.36 | 150.36 | 167.77 | 10.19 | 10.10 | 9.00 | 9.65 | 9.25 | 9.64 |
| G33 | 73.00 | 78.00 | 71.00 | 72.00 | 70.00 | 72.80 | 175.95 | 179.81 | 180.06 | 149.78 | 164.71 | 170.06 | 10.75 | 9.70 | 9.90 | 10.55 | 11.30 | 10.44 |
| G34 | 68.50 | 77.00 | 70.00 | 70.00 | 66.50 | 70.40 | 178.70 | 188.36 | 170.72 | 174.98 | 169.12 | 176.38 | 10.46 | 11.75 | 11.20 | 11.25 | 8.65 | 10.66 |
| G35 | 74.50 | 79.00 | 74.00 | 74.50 | 70.00 | 74.40 | 178.80 | 198.75 | 154.95 | 150.28 | 142.49 | 165.05 | 9.08 | 9.70 | 9.85 | 9.30 | 7.80 | 9.15 |
| G36 | 75.00 | 79.00 | 69.00 | 72.00 | 70.00 | 73.00 | 174.29 | 177.38 | 151.95 | 142.32 | 129.12 | 155.01 | 10.58 | 9.90 | 8.40 | 10.30 | 7.80 | 9.40 |
| G37 | 69.50 | 82.50 | 88.00 | 78.00 | 76.00 | 78.80 | 135.40 | 143.76 | 162.96 | 129.47 | 146.78 | 143.67 | 9.52 | 9.96 | 10.79 | 8.20 | 9.75 | 9.64 |
| G38 | 63.00 | 73.00 | 70.00 | 70.00 | 67.00 | 68.60 | 189.55 | 203.86 | 183.44 | 187.92 | 125.34 | 178.02 | 9.86 | 10.30 | 10.90 | 10.30 | 6.60 | 9.59 |
| G39 | 66.00 | 72.50 | 66.00 | 64.00 | 64.00 | 66.50 | 163.40 | 155.49 | 175.93 | 150.60 | 125.19 | 154.12 | 12.18 | 11.80 | 12.40 | 11.00 | 8.80 | 11.24 |
| G40 | 75.00 | 78.50 | 74.00 | 75.00 | 71.00 | 74.70 | 151.27 | 162.82 | 123.82 | 134.66 | 113.60 | 137.23 | 9.26 | 10.05 | 8.95 | 8.80 | 8.00 | 9.01 |
| G41 | 68.50 | 73.00 | 70.00 | 69.50 | 67.00 | 69.60 | 137.39 | 127.55 | 118.69 | 130.10 | 149.25 | 132.60 | 9.95 | 10.50 | 8.45 | 10.20 | 11.00 | 10.02 |
| G42 | 80.00 | 70.50 | 66.00 | 63.50 | 64.00 | 68.80 | 134.85 | 128.31 | 113.50 | 121.04 | 149.34 | 129.41 | 10.22 | 7.80 | 8.00 | 9.10 | 10.20 | 9.06 |
| G43 | 71.50 | 69.50 | 74.00 | 68.00 | 70.00 | 70.60 | 144.25 | 169.42 | 145.83 | 123.44 | 138.45 | 144.28 | 9.78 | 10.20 | 11.40 | 11.10 | 6.90 | 9.88 |
| G44 | 56.50 | 60.50 | 58.00 | 55.50 | 54.00 | 56.90 | 223.65 | 203.29 | 226.50 | 226.45 | 152.23 | 206.42 | 9.52 | 8.71 | 9.75 | 8.20 | 7.45 | 8.73 |
| G45 | 63.50 | 69.00 | 64.00 | 61.00 | 63.00 | 64.10 | 187.25 | 182.35 | 189.64 | 168.83 | 206.16 | 186.85 | 7.55 | 9.11 | 8.40 | 7.89 | 9.00 | 8.39 |
| G46 | 88.50 | 92.00 | 84.00 | 90.00 | 81.00 | 87.10 | 169.67 | 188.25 | 132.97 | 157.52 | 181.48 | 165.98 | 11.07 | 11.40 | 10.05 | 10.45 | 10.20 | 10.63 |
| G47 | 74.50 | 79.50 | 67.50 | 77.00 | 76.00 | 74.90 | 189.30 | 216.88 | 111.13 | 173.40 | 178.13 | 173.77 | 8.81 | 9.90 | 10.20 | 8.45 | 9.15 | 9.30 |
| G48 | 63.50 | 78.00 | 73.00 | 72.50 | 74.50 | 72.30 | 168.61 | 175.23 | 152.99 | 151.28 | 136.25 | 156.87 | 9.37 | 9.50 | 8.55 | 8.80 | 8.30 | 8.90 |
| G49 | 74.00 | 76.00 | 72.00 | 74.00 | 70.00 | 73.20 | 124.90 | 137.59 | 152.74 | 133.97 | 115.08 | 132.86 | 9.40 | 10.40 | 10.56 | 9.69 | 8.80 | 9.77 |
| G50 | 79.00 | 81.50 | 78.00 | 75.50 | 73.00 | 77.40 | 138.00 | 148.19 | 178.63 | 114.39 | 145.00 | 144.84 | 8.66 | 9.00 | 9.20 | 9.40 | 8.20 | 8.89 |
| G51 | 59.00 | 69.50 | 67.00 | 65.00 | 64.00 | 64.90 | 135.20 | 145.21 | 157.17 | 128.43 | 103.67 | 133.94 | 9.26 | 9.50 | 10.90 | 8.90 | 8.40 | 9.39 |
| G52 | 80.50 | 85.50 | 80.50 | 76.00 | 72.50 | 79.00 | 119.61 | 130.60 | 133.25 | 108.59 | 97.26 | 117.86 | 9.74 | 10.20 | 10.90 | 10.00 | 9.10 | 9.99 |
| G53 | 74.50 | 76.00 | 73.00 | 73.00 | 72.00 | 73.70 | 128.60 | 150.97 | 165.97 | 131.66 | 153.19 | 146.08 | 11.23 | 10.90 | 11.80 | 10.40 | 8.90 | 10.65 |
| G54 | 63.50 | 76.50 | 74.00 | 76.50 | 71.00 | 72.30 | 130.10 | 137.41 | 150.03 | 125.92 | 132.14 | 135.12 | 11.20 | 11.00 | 12.80 | 12.85 | 7.90 | 11.15 |
| G55 | 80.00 | 82.00 | 78.00 | 82.00 | 79.00 | 80.20 | 116.15 | 105.52 | 117.80 | 97.99 | 114.47 | 110.38 | 9.82 | 9.50 | 10.60 | 10.40 | 7.30 | 9.52 |
| G56 | 89.00 | 94.50 | 88.50 | 93.00 | 92.50 | 91.50 | 134.10 | 170.75 | 113.57 | 122.57 | 188.87 | 145.97 | 9.79 | 9.80 | 10.40 | 10.30 | 10.70 | 10.20 |
| G57 | 56.50 | 75.50 | 72.00 | 72.00 | 70.00 | 69.20 | 134.73 | 138.33 | 135.74 | 130.94 | 113.19 | 130.59 | 10.06 | 9.40 | 9.80 | 9.90 | 8.20 | 9.47 |
| G58 | 71.50 | 75.50 | 70.50 | 74.50 | 69.50 | 72.30 | 150.08 | 164.59 | 117.40 | 134.66 | 132.75 | 139.90 | 11.20 | 10.40 | 11.80 | 10.00 | 8.50 | 10.38 |
| G59 | 74.00 | 75.50 | 74.00 | 67.50 | 69.50 | 72.10 | 121.57 | 115.74 | 122.81 | 105.64 | 105.64 | 114.28 | 10.34 | 11.10 | 11.95 | 9.90 | 8.45 | 10.35 |
| G60 | 84.00 | 70.00 | 67.00 | 68.00 | 63.50 | 70.50 | 154.36 | 149.93 | 141.62 | 143.99 | 184.74 | 154.93 | 9.47 | 9.20 | 9.90 | 10.50 | 8.80 | 9.57 |
| G61 | 56.50 | 71.50 | 70.00 | 69.00 | 66.50 | 66.70 | 115.28 | 117.31 | 128.34 | 105.53 | 110.25 | 115.34 | 9.48 | 9.01 | 9.90 | 9.20 | 7.40 | 9.00 |
| G62 | 69.00 | 71.50 | 68.00 | 68.00 | 65.50 | 68.40 | 133.40 | 140.19 | 123.92 | 125.93 | 120.96 | 128.88 | 10.99 | 10.65 | 10.20 | 10.45 | 6.60 | 9.78 |
| G63 | 69.50 | 74.00 | 71.00 | 70.50 | 66.00 | 70.20 | 203.38 | 192.36 | 174.92 | 176.06 | 131.87 | 175.72 | 10.64 | 11.45 | 10.10 | 10.90 | 9.50 | 10.52 |
| G64 | 70.50 | 72.50 | 69.00 | 69.00 | 67.00 | 69.60 | 197.10 | 227.18 | 126.35 | 190.26 | 163.88 | 180.95 | 11.13 | 10.70 | 11.70 | 12.31 | 11.10 | 11.39 |
| G65 | 70.00 | 74.50 | 69.50 | 70.00 | 68.00 | 70.40 | 128.00 | 123.11 | 151.26 | 114.48 | 140.76 | 131.52 | 10.80 | 10.90 | 11.70 | 8.80 | 11.10 | 10.66 |
| G66 | 81.50 | 76.00 | 75.50 | 73.00 | 69.50 | 75.10 | 170.28 | 174.58 | 212.02 | 163.55 | 190.36 | 182.16 | 10.90 | 11.20 | 11.80 | 7.65 | 11.30 | 10.57 |
| G67 | 51.00 | 51.00 | 49.00 | 50.00 | 46.50 | 49.50 | 187.84 | 195.53 | 177.02 | 186.32 | 237.14 | 196.77 | 6.14 | 5.70 | 6.30 | 6.20 | 10.90 | 7.05 |
| G68 | 74.50 | 77.50 | 72.50 | 72.00 | 67.00 | 72.70 | 112.15 | 112.07 | 104.73 | 107.79 | 115.13 | 110.37 | 10.99 | 10.50 | 11.00 | 12.12 | 7.80 | 10.48 |
| G69 | 74.00 | 79.00 | 73.00 | 72.00 | 69.00 | 73.40 | 215.45 | 232.95 | 176.45 | 223.29 | 206.12 | 210.85 | 10.99 | 10.60 | 10.90 | 11.00 | 8.10 | 10.32 |
| G70 | 67.50 | 76.00 | 65.00 | 72.50 | 71.50 | 70.50 | 117.29 | 150.60 | 104.59 | 122.63 | 120.89 | 123.20 | 10.28 | 10.50 | 9.60 | 9.90 | 8.50 | 9.76 |
| G71 | 74.00 | 78.50 | 74.00 | 75.00 | 68.50 | 74.00 | 112.31 | 119.50 | 153.30 | 110.68 | 121.74 | 123.51 | 11.17 | 11.90 | 12.80 | 9.40 | 10.90 | 11.23 |
| G72 | 82.00 | 84.50 | 77.00 | 81.00 | 78.00 | 80.50 | 104.30 | 102.78 | 125.44 | 101.95 | 115.44 | 109.98 | 11.24 | 11.00 | 11.40 | 9.30 | 9.30 | 10.45 |
| G73 | 78.00 | 82.50 | 78.00 | 81.00 | 78.00 | 79.50 | 136.81 | 136.19 | 114.76 | 121.17 | 122.87 | 126.36 | 9.77 | 9.70 | 9.90 | 10.00 | 9.10 | 9.69 |
| G74 | 67.50 | 75.50 | 72.00 | 74.50 | 72.00 | 72.30 | 119.10 | 160.38 | 117.56 | 121.93 | 99.14 | 123.62 | 10.49 | 10.90 | 10.00 | 9.80 | 9.80 | 10.20 |
| G75 | 73.50 | 72.50 | 70.00 | 69.00 | 66.00 | 70.20 | 148.15 | 151.30 | 125.00 | 140.88 | 118.72 | 136.81 | 11.17 | 12.20 | 10.80 | 9.80 | 8.20 | 10.43 |
| G76 | 75.00 | 73.00 | 72.00 | 70.50 | 68.00 | 71.70 | 129.02 | 137.89 | 115.62 | 128.30 | 115.17 | 125.20 | 9.46 | 11.40 | 9.20 | 10.40 | 9.20 | 9.93 |
| G77 | 66.50 | 71.50 | 67.50 | 70.00 | 66.00 | 68.30 | 108.77 | 113.47 | 101.81 | 101.02 | 117.03 | 108.42 | 11.26 | 10.35 | 9.60 | 8.40 | 11.10 | 10.14 |
| G78 | 73.50 | 69.50 | 68.00 | 66.00 | 67.00 | 68.80 | 126.61 | 110.41 | 103.24 | 94.49 | 115.94 | 110.14 | 10.35 | 10.30 | 9.35 | 8.80 | 10.80 | 9.92 |
| G79 | 58.00 | 71.00 | 68.50 | 72.00 | 68.50 | 67.60 | 136.13 | 141.91 | 116.16 | 127.01 | 133.97 | 131.04 | 10.07 | 11.00 | 9.40 | 9.80 | 10.00 | 10.05 |
| G80 | 72.50 | 78.50 | 72.50 | 72.00 | 72.00 | 73.50 | 180.73 | 186.52 | 152.25 | 164.79 | 155.30 | 167.92 | 10.81 | 10.90 | 9.55 | 10.40 | 9.90 | 10.31 |
| G81 | 68.50 | 72.50 | 66.50 | 64.00 | 67.00 | 67.70 | 190.83 | 192.83 | 178.96 | 170.75 | 180.86 | 182.85 | 7.99 | 8.20 | 7.50 | 8.75 | 8.50 | 8.19 |
| G82 | 88.00 | 95.50 | 86.00 | 90.00 | 81.00 | 88.10 | 129.89 | 133.44 | 115.85 | 101.69 | 122.61 | 120.69 | 9.69 | 9.90 | 10.50 | 8.70 | 10.20 | 9.80 |
| G83 | 81.50 | 82.00 | 75.00 | 78.00 | 73.00 | 77.90 | 103.39 | 105.69 | 94.51 | 104.13 | 120.66 | 105.68 | 11.42 | 11.50 | 10.40 | 10.15 | 12.20 | 11.13 |
| G84 | 69.50 | 78.00 | 73.50 | 71.50 | 68.50 | 72.20 | 130.51 | 129.14 | 115.30 | 119.70 | 133.64 | 125.66 | 10.89 | 10.90 | 10.00 | 10.10 | 12.25 | 10.83 |
| G85 | 66.00 | 76.50 | 71.00 | 73.50 | 70.00 | 71.40 | 180.15 | 188.55 | 164.26 | 155.88 | 190.94 | 175.95 | 11.06 | 11.20 | 9.58 | 11.00 | 11.70 | 10.91 |
| G86 | 76.00 | 79.00 | 75.00 | 71.50 | 69.50 | 74.20 | 116.92 | 125.01 | 102.34 | 108.06 | 122.27 | 114.92 | 10.39 | 10.40 | 9.30 | 9.60 | 11.10 | 10.16 |
| G87 | 65.00 | 79.00 | 73.50 | 76.50 | 72.00 | 73.20 | 117.26 | 121.06 | 104.57 | 109.27 | 131.35 | 116.70 | 11.67 | 12.30 | 11.10 | 10.50 | 12.80 | 11.67 |
| G88 | 81.00 | 72.00 | 79.00 | 77.50 | 74.50 | 76.80 | 113.65 | 121.55 | 107.26 | 104.30 | 119.03 | 113.16 | 10.14 | 10.20 | 10.50 | 10.00 | 11.00 | 10.37 |
| G89 | 78.50 | 81.00 | 66.00 | 68.50 | 73.50 | 73.50 | 130.05 | 142.23 | 117.59 | 124.85 | 132.85 | 129.51 | 10.32 | 11.25 | 9.25 | 10.00 | 10.70 | 10.30 |
| G90 | 76.50 | 79.50 | 74.50 | 73.50 | 70.00 | 74.80 | 171.99 | 171.29 | 153.07 | 169.50 | 157.49 | 164.66 | 12.23 | 12.20 | 10.40 | 10.80 | 11.80 | 11.49 |
| G91 | 81.50 | 84.00 | 79.50 | 77.50 | 72.00 | 78.90 | 119.73 | 129.63 | 111.40 | 134.74 | 136.61 | 126.42 | 10.52 | 10.38 | 10.10 | 11.10 | 11.80 | 10.78 |
| G92 | 79.00 | 81.50 | 77.00 | 77.00 | 73.00 | 77.50 | 92.68 | 91.92 | 98.89 | 93.52 | 108.33 | 97.07 | 7.59 | 8.30 | 9.10 | 7.10 | 9.10 | 8.24 |
| G93 | 76.50 | 81.50 | 76.00 | 74.00 | 74.50 | 76.50 | 116.32 | 126.59 | 105.80 | 110.38 | 138.54 | 119.52 | 11.07 | 11.00 | 9.00 | 9.60 | 11.45 | 10.42 |
| G94 | 59.50 | 78.50 | 67.00 | 73.00 | 72.50 | 70.10 | 94.74 | 96.14 | 78.67 | 85.54 | 113.09 | 93.63 | 10.65 | 11.00 | 9.50 | 9.80 | 11.05 | 10.40 |
| G95 | 76.50 | 81.50 | 75.50 | 72.50 | 66.50 | 74.50 | 179.60 | 153.73 | 149.86 | 168.50 | 163.58 | 163.05 | 10.11 | 10.60 | 9.20 | 9.60 | 10.20 | 9.94 |
| Mean | 72.22 | 77.12 | 72.81 | 72.95 | 70.60 | 73.14 | 143.30 | 150.74 | 134.75 | 134.15 | 136.32 | 139.85 | 10.22 | 10.32 | 9.84 | 9.77 | 9.27 | 9.88 |

Supplementary Table 2 Continued….

| GEN | LFL | | | | | | LFW | | | | | | LAI | | | | | |
| --- | --- | --- | --- | --- | --- | --- | --- | --- | --- | --- | --- | --- | --- | --- | --- | --- | --- | --- |
|  | E1 | E2 | E3 | E4 | E5 | AVG | E1 | E2 | E3 | E4 | E5 | AVG | E1 | E2 | E3 | E4 | E5 | AVG |
| G1 | 49.23 | 64.25 | 48.11 | 53.29 | 73.56 | 57.69 | 5.27 | 5.96 | 3.92 | 5.03 | 7.11 | 5.46 | 2.17 | 3.36 | 1.08 | 2.04 | 4.03 | 2.54 |
| G2 | 62.65 | 63.65 | 61.54 | 60.42 | 72.09 | 64.07 | 7.80 | 7.86 | 7.60 | 7.90 | 8.41 | 7.92 | 3.87 | 3.67 | 3.49 | 3.39 | 3.28 | 3.54 |
| G3 | 70.23 | 74.09 | 62.46 | 67.17 | 67.60 | 68.31 | 8.20 | 8.49 | 7.04 | 9.10 | 6.99 | 7.96 | 5.14 | 5.70 | 2.67 | 5.50 | 3.61 | 4.52 |
| G4 | 63.00 | 63.94 | 48.29 | 62.20 | 60.55 | 59.60 | 6.87 | 7.17 | 5.73 | 6.66 | 6.90 | 6.67 | 3.82 | 4.19 | 2.07 | 3.59 | 3.38 | 3.41 |
| G5 | 63.40 | 68.41 | 55.73 | 63.37 | 64.35 | 63.05 | 7.35 | 7.59 | 6.48 | 7.22 | 5.19 | 6.76 | 3.34 | 3.91 | 2.62 | 3.28 | 1.93 | 3.02 |
| G6 | 52.60 | 62.30 | 52.35 | 55.01 | 72.92 | 59.04 | 7.11 | 8.39 | 7.95 | 6.82 | 7.93 | 7.64 | 3.27 | 4.63 | 2.54 | 3.37 | 4.66 | 3.69 |
| G7 | 56.21 | 63.37 | 58.46 | 52.71 | 66.32 | 59.41 | 6.03 | 6.44 | 7.00 | 5.66 | 7.72 | 6.57 | 3.35 | 3.87 | 3.93 | 2.62 | 4.15 | 3.58 |
| G8 | 69.95 | 70.29 | 65.65 | 67.65 | 80.85 | 70.88 | 8.42 | 8.13 | 6.64 | 7.76 | 8.03 | 7.80 | 5.60 | 4.95 | 3.42 | 4.81 | 5.22 | 4.80 |
| G9 | 64.25 | 67.74 | 61.45 | 61.60 | 74.68 | 65.94 | 7.22 | 7.12 | 6.98 | 6.13 | 8.16 | 7.12 | 4.22 | 4.41 | 3.45 | 3.32 | 5.46 | 4.17 |
| G10 | 51.50 | 63.78 | 49.71 | 58.44 | 47.40 | 54.17 | 8.02 | 7.95 | 6.48 | 7.44 | 6.69 | 7.32 | 3.69 | 4.51 | 2.49 | 3.94 | 1.92 | 3.31 |
| G11 | 55.44 | 66.04 | 49.87 | 54.68 | 49.53 | 55.11 | 6.94 | 6.20 | 5.45 | 5.97 | 6.11 | 6.13 | 3.60 | 4.18 | 2.39 | 2.97 | 2.34 | 3.09 |
| G12 | 52.55 | 66.73 | 53.08 | 56.04 | 51.48 | 55.98 | 8.13 | 6.96 | 6.94 | 7.36 | 7.90 | 7.46 | 3.34 | 3.89 | 2.85 | 3.30 | 2.19 | 3.11 |
| G13 | 53.65 | 58.42 | 53.16 | 55.59 | 68.57 | 57.88 | 7.00 | 7.11 | 5.84 | 6.20 | 6.95 | 6.62 | 3.26 | 3.70 | 2.65 | 3.17 | 3.12 | 3.18 |
| G14 | 65.94 | 72.30 | 64.47 | 71.12 | 73.86 | 69.54 | 8.18 | 8.40 | 7.70 | 7.93 | 7.74 | 7.99 | 4.62 | 5.54 | 4.17 | 5.05 | 4.40 | 4.76 |
| G15 | 63.95 | 68.22 | 63.65 | 74.32 | 67.45 | 67.52 | 8.95 | 9.12 | 7.41 | 9.08 | 5.93 | 8.10 | 5.52 | 6.33 | 4.34 | 6.68 | 2.66 | 5.10 |
| G16 | 61.93 | 65.98 | 60.93 | 68.43 | 63.26 | 64.11 | 8.32 | 8.42 | 7.52 | 7.79 | 6.82 | 7.77 | 4.38 | 5.01 | 3.64 | 4.70 | 3.47 | 4.24 |
| G17 | 73.62 | 72.10 | 72.40 | 72.55 | 100.78 | 78.29 | 3.80 | 3.79 | 4.53 | 3.60 | 3.63 | 3.87 | 2.57 | 1.71 | 1.89 | 1.67 | 2.25 | 2.01 |
| G18 | 66.04 | 70.29 | 65.82 | 62.64 | 69.76 | 66.91 | 6.66 | 7.43 | 7.21 | 6.09 | 7.72 | 7.02 | 2.86 | 3.80 | 2.85 | 2.54 | 4.10 | 3.23 |
| G19 | 61.80 | 68.16 | 62.75 | 59.76 | 66.06 | 63.70 | 6.05 | 6.27 | 6.24 | 5.62 | 8.56 | 6.55 | 2.62 | 2.87 | 2.43 | 2.02 | 3.98 | 2.78 |
| G20 | 58.78 | 61.12 | 59.61 | 55.76 | 61.01 | 59.25 | 5.05 | 5.36 | 6.23 | 5.13 | 5.88 | 5.53 | 1.97 | 1.94 | 2.63 | 1.52 | 1.69 | 1.95 |
| G21 | 62.80 | 60.79 | 55.68 | 58.98 | 74.56 | 62.56 | 9.18 | 8.28 | 8.80 | 7.62 | 8.43 | 8.46 | 6.46 | 5.26 | 4.25 | 4.66 | 5.52 | 5.23 |
| G22 | 62.15 | 65.11 | 57.80 | 63.93 | 71.75 | 64.15 | 8.71 | 8.52 | 8.00 | 8.16 | 5.92 | 7.86 | 4.27 | 4.40 | 3.36 | 4.11 | 3.11 | 3.85 |
| G23 | 65.30 | 68.67 | 67.93 | 63.17 | 68.77 | 66.77 | 7.17 | 8.00 | 6.91 | 7.73 | 5.93 | 7.15 | 4.35 | 5.26 | 4.64 | 2.77 | 3.55 | 4.11 |
| G24 | 55.45 | 65.49 | 55.01 | 56.87 | 53.16 | 57.20 | 7.90 | 8.08 | 7.64 | 7.62 | 6.94 | 7.64 | 3.83 | 4.73 | 3.85 | 3.62 | 2.35 | 3.68 |
| G25 | 62.85 | 65.73 | 58.41 | 56.21 | 74.20 | 63.48 | 7.16 | 7.06 | 7.64 | 6.02 | 7.56 | 7.08 | 4.27 | 4.02 | 3.75 | 2.88 | 3.97 | 3.78 |
| G26 | 70.40 | 74.39 | 73.74 | 65.85 | 75.80 | 72.03 | 7.99 | 6.72 | 6.09 | 7.13 | 6.71 | 6.93 | 4.83 | 4.26 | 3.48 | 3.25 | 3.75 | 3.91 |
| G27 | 57.50 | 57.96 | 55.16 | 56.88 | 62.33 | 57.96 | 5.40 | 6.03 | 6.00 | 6.14 | 6.95 | 6.10 | 2.21 | 2.72 | 1.96 | 2.79 | 3.68 | 2.67 |
| G28 | 62.75 | 61.30 | 74.60 | 55.92 | 59.08 | 62.73 | 6.16 | 6.50 | 7.56 | 5.66 | 7.74 | 6.72 | 3.52 | 3.87 | 5.09 | 3.17 | 3.01 | 3.74 |
| G29 | 51.00 | 54.64 | 54.61 | 49.25 | 73.08 | 56.52 | 5.63 | 6.37 | 6.76 | 4.44 | 5.97 | 5.84 | 2.40 | 2.98 | 3.49 | 1.72 | 3.65 | 2.85 |
| G30 | 50.70 | 70.46 | 78.73 | 54.61 | 51.62 | 61.22 | 8.00 | 8.04 | 7.81 | 7.37 | 7.49 | 7.74 | 3.73 | 5.09 | 5.04 | 3.72 | 3.26 | 4.17 |
| G31 | 70.60 | 74.78 | 69.86 | 72.43 | 62.38 | 70.01 | 8.20 | 8.57 | 8.02 | 7.27 | 5.54 | 7.52 | 4.39 | 5.51 | 4.32 | 3.23 | 2.81 | 4.05 |
| G32 | 70.65 | 79.51 | 73.05 | 80.79 | 78.86 | 76.57 | 9.11 | 8.07 | 7.95 | 7.79 | 8.14 | 8.21 | 5.47 | 5.46 | 4.38 | 5.08 | 4.97 | 5.07 |
| G33 | 61.75 | 59.78 | 61.37 | 65.71 | 73.74 | 64.47 | 7.30 | 7.08 | 7.01 | 6.94 | 8.01 | 7.27 | 4.07 | 3.45 | 3.59 | 4.01 | 5.63 | 4.15 |
| G34 | 65.64 | 72.88 | 64.46 | 62.56 | 62.88 | 65.68 | 7.30 | 7.83 | 6.77 | 7.57 | 6.85 | 7.26 | 4.17 | 5.60 | 4.08 | 4.45 | 3.13 | 4.29 |
| G35 | 58.35 | 64.28 | 56.11 | 62.35 | 75.29 | 63.28 | 6.52 | 7.20 | 5.10 | 7.77 | 7.72 | 6.86 | 2.88 | 3.75 | 2.36 | 3.77 | 3.76 | 3.31 |
| G36 | 69.80 | 79.89 | 73.39 | 74.55 | 73.65 | 74.26 | 6.62 | 7.71 | 5.63 | 7.96 | 6.73 | 6.93 | 4.08 | 5.11 | 2.90 | 5.10 | 3.25 | 4.09 |
| G37 | 56.20 | 60.12 | 62.46 | 58.73 | 59.31 | 59.37 | 7.11 | 8.45 | 8.11 | 7.95 | 7.00 | 7.73 | 3.17 | 4.23 | 4.55 | 3.19 | 3.40 | 3.71 |
| G38 | 62.30 | 72.31 | 63.52 | 63.33 | 57.83 | 63.86 | 7.21 | 7.37 | 6.97 | 7.66 | 5.53 | 6.95 | 3.69 | 4.58 | 4.05 | 4.18 | 1.78 | 3.66 |
| G39 | 53.72 | 54.31 | 63.69 | 52.10 | 46.81 | 54.13 | 6.40 | 7.26 | 7.36 | 6.27 | 6.76 | 6.81 | 3.49 | 3.89 | 4.84 | 3.01 | 2.31 | 3.51 |
| G40 | 62.70 | 62.60 | 53.74 | 57.39 | 58.31 | 58.95 | 7.80 | 9.28 | 7.39 | 8.32 | 7.50 | 8.06 | 3.78 | 4.87 | 2.98 | 3.52 | 2.93 | 3.62 |
| G41 | 54.60 | 57.86 | 52.48 | 52.20 | 66.69 | 56.77 | 7.52 | 7.48 | 7.47 | 6.18 | 8.10 | 7.35 | 3.41 | 3.80 | 2.76 | 2.75 | 4.99 | 3.54 |
| G42 | 47.15 | 51.78 | 44.62 | 46.22 | 52.83 | 48.52 | 7.32 | 6.47 | 6.43 | 6.03 | 5.53 | 6.35 | 2.95 | 2.18 | 1.91 | 2.12 | 2.48 | 2.33 |
| G43 | 62.77 | 66.10 | 65.67 | 62.56 | 64.94 | 64.41 | 7.36 | 7.89 | 8.10 | 7.03 | 5.88 | 7.25 | 3.77 | 4.45 | 5.05 | 4.06 | 2.19 | 3.91 |
| G44 | 61.30 | 64.30 | 64.00 | 57.55 | 63.73 | 62.17 | 5.23 | 5.53 | 6.33 | 5.22 | 7.93 | 6.05 | 2.55 | 2.59 | 3.31 | 2.05 | 3.15 | 2.73 |
| G45 | 63.45 | 63.45 | 64.99 | 60.58 | 77.66 | 66.02 | 5.51 | 5.74 | 6.61 | 5.79 | 8.71 | 6.47 | 2.19 | 2.78 | 3.02 | 2.32 | 5.08 | 3.08 |
| G46 | 65.10 | 67.69 | 64.46 | 57.70 | 68.04 | 64.60 | 7.47 | 6.47 | 6.89 | 5.13 | 6.33 | 6.46 | 4.48 | 4.18 | 3.73 | 2.59 | 3.67 | 3.73 |
| G47 | 64.75 | 85.03 | 62.53 | 68.04 | 67.90 | 69.65 | 6.06 | 6.44 | 6.24 | 6.11 | 7.29 | 6.43 | 2.89 | 4.54 | 3.32 | 2.96 | 3.78 | 3.50 |
| G48 | 61.60 | 83.24 | 61.56 | 60.32 | 70.48 | 67.44 | 7.05 | 8.10 | 7.02 | 7.06 | 5.97 | 7.04 | 3.39 | 5.35 | 3.09 | 3.14 | 2.91 | 3.57 |
| G49 | 63.75 | 66.08 | 69.57 | 62.82 | 58.87 | 64.22 | 7.04 | 8.07 | 6.91 | 6.88 | 6.24 | 7.03 | 3.51 | 4.64 | 4.25 | 3.48 | 2.68 | 3.71 |
| G50 | 52.60 | 59.34 | 65.63 | 53.54 | 56.74 | 57.57 | 6.09 | 6.75 | 5.98 | 6.15 | 7.45 | 6.49 | 2.31 | 3.01 | 3.01 | 2.59 | 2.89 | 2.76 |
| G51 | 64.11 | 63.18 | 82.81 | 57.89 | 56.22 | 64.84 | 6.13 | 6.90 | 6.76 | 5.82 | 8.38 | 6.80 | 3.03 | 3.47 | 5.10 | 2.51 | 3.31 | 3.48 |
| G52 | 60.60 | 64.53 | 62.93 | 54.56 | 62.91 | 61.10 | 5.89 | 7.62 | 7.31 | 6.14 | 6.79 | 6.75 | 2.89 | 4.19 | 4.19 | 2.82 | 3.25 | 3.47 |
| G53 | 66.55 | 62.33 | 83.48 | 55.89 | 62.41 | 66.13 | 6.30 | 7.27 | 5.69 | 7.05 | 7.07 | 6.67 | 3.92 | 4.11 | 4.68 | 3.43 | 3.29 | 3.89 |
| G54 | 60.12 | 60.54 | 74.52 | 57.32 | 64.76 | 63.45 | 6.27 | 7.35 | 6.25 | 6.60 | 8.84 | 7.06 | 3.52 | 4.07 | 4.95 | 4.05 | 3.76 | 4.07 |
| G55 | 57.25 | 60.11 | 61.24 | 54.61 | 65.47 | 59.74 | 7.37 | 7.28 | 7.15 | 7.40 | 7.75 | 7.39 | 3.45 | 3.47 | 3.86 | 3.52 | 3.09 | 3.48 |
| G56 | 66.00 | 75.62 | 60.76 | 59.45 | 64.36 | 65.24 | 6.84 | 7.99 | 7.08 | 6.88 | 6.95 | 7.15 | 3.69 | 4.93 | 3.75 | 3.52 | 4.02 | 3.98 |
| G57 | 53.25 | 61.95 | 53.84 | 57.86 | 61.71 | 57.72 | 7.26 | 6.82 | 6.41 | 7.10 | 5.33 | 6.58 | 3.25 | 3.32 | 2.83 | 3.40 | 2.26 | 3.01 |
| G58 | 61.33 | 76.53 | 64.39 | 67.46 | 62.42 | 66.43 | 7.69 | 7.56 | 4.84 | 7.22 | 8.84 | 7.23 | 4.42 | 5.02 | 3.08 | 4.07 | 3.91 | 4.10 |
| G59 | 57.35 | 58.42 | 61.46 | 57.19 | 63.45 | 59.57 | 7.97 | 9.08 | 7.69 | 8.02 | 7.89 | 8.13 | 3.94 | 4.90 | 4.71 | 3.78 | 3.54 | 4.17 |
| G60 | 58.51 | 62.62 | 59.59 | 59.16 | 76.19 | 63.21 | 7.67 | 8.80 | 7.28 | 8.07 | 7.26 | 7.82 | 3.54 | 4.22 | 3.59 | 4.18 | 4.05 | 3.92 |
| G61 | 63.67 | 61.81 | 64.52 | 62.20 | 56.72 | 61.78 | 8.50 | 9.33 | 7.17 | 9.15 | 4.78 | 7.79 | 4.28 | 4.34 | 3.82 | 4.38 | 1.69 | 3.70 |
| G62 | 62.15 | 62.62 | 58.45 | 67.83 | 74.96 | 65.20 | 8.22 | 9.09 | 7.79 | 8.85 | 6.39 | 8.07 | 4.69 | 5.09 | 3.89 | 5.24 | 2.65 | 4.31 |
| G63 | 65.70 | 66.48 | 62.74 | 71.42 | 63.76 | 66.02 | 7.31 | 7.06 | 5.58 | 7.14 | 6.04 | 6.62 | 4.26 | 4.51 | 2.94 | 4.65 | 3.07 | 3.89 |
| G64 | 77.32 | 78.17 | 74.36 | 79.50 | 83.89 | 78.65 | 8.33 | 8.45 | 7.22 | 8.89 | 8.91 | 8.36 | 5.98 | 5.88 | 5.21 | 7.24 | 6.95 | 6.25 |
| G65 | 57.10 | 58.72 | 66.11 | 55.74 | 55.48 | 58.63 | 8.51 | 8.95 | 6.84 | 8.06 | 9.12 | 8.30 | 4.38 | 4.79 | 4.41 | 3.30 | 4.70 | 4.32 |
| G66 | 67.85 | 71.71 | 72.66 | 64.14 | 80.84 | 71.44 | 8.20 | 7.93 | 7.95 | 8.21 | 7.38 | 7.93 | 5.04 | 5.34 | 5.69 | 3.36 | 5.60 | 5.01 |
| G67 | 53.80 | 56.64 | 55.83 | 51.20 | 67.97 | 57.09 | 3.32 | 4.42 | 2.27 | 3.00 | 2.54 | 3.11 | 0.91 | 1.19 | 0.67 | 0.80 | 1.56 | 1.03 |
| G68 | 66.84 | 69.16 | 64.06 | 62.07 | 81.15 | 68.65 | 6.91 | 7.94 | 6.98 | 6.98 | 7.89 | 7.34 | 4.22 | 4.81 | 4.10 | 4.41 | 4.16 | 4.34 |
| G69 | 81.45 | 77.87 | 73.59 | 79.34 | 76.62 | 77.77 | 6.22 | 6.20 | 6.18 | 7.11 | 7.26 | 6.59 | 4.66 | 4.27 | 4.14 | 5.19 | 3.75 | 4.40 |
| G70 | 59.12 | 62.82 | 48.44 | 51.27 | 53.32 | 54.99 | 6.03 | 7.04 | 6.05 | 6.08 | 7.26 | 6.49 | 3.06 | 3.89 | 2.35 | 2.57 | 2.73 | 2.92 |
| G71 | 49.70 | 54.68 | 70.81 | 48.03 | 59.92 | 56.63 | 6.85 | 7.66 | 5.63 | 7.04 | 8.14 | 7.06 | 3.17 | 4.14 | 4.26 | 2.67 | 4.45 | 3.74 |
| G72 | 59.17 | 63.06 | 58.90 | 58.99 | 64.83 | 60.99 | 7.66 | 8.21 | 6.07 | 7.53 | 7.47 | 7.39 | 4.24 | 4.74 | 3.41 | 3.44 | 3.76 | 3.92 |
| G73 | 53.47 | 57.53 | 56.07 | 52.55 | 66.63 | 57.25 | 6.03 | 6.32 | 5.50 | 6.39 | 6.21 | 6.09 | 2.62 | 2.93 | 2.53 | 2.79 | 3.14 | 2.80 |
| G74 | 58.40 | 60.60 | 57.10 | 55.33 | 61.53 | 58.59 | 6.22 | 6.59 | 5.49 | 7.04 | 6.94 | 6.45 | 3.19 | 3.62 | 2.61 | 3.18 | 3.50 | 3.22 |
| G75 | 70.10 | 76.22 | 61.38 | 64.65 | 69.73 | 68.42 | 8.32 | 8.79 | 7.06 | 9.10 | 9.03 | 8.46 | 5.42 | 6.79 | 3.91 | 4.82 | 4.32 | 5.05 |
| G76 | 71.98 | 73.48 | 65.27 | 67.25 | 55.89 | 66.77 | 6.39 | 6.83 | 5.74 | 7.97 | 5.40 | 6.47 | 3.63 | 4.76 | 2.89 | 4.65 | 2.33 | 3.65 |
| G77 | 47.66 | 57.50 | 44.60 | 46.30 | 57.96 | 50.81 | 5.04 | 4.09 | 4.10 | 4.25 | 5.07 | 4.51 | 2.27 | 2.04 | 1.47 | 1.39 | 2.71 | 1.97 |
| G78 | 52.67 | 55.49 | 52.45 | 48.82 | 56.07 | 53.10 | 7.35 | 7.94 | 6.00 | 7.09 | 6.78 | 7.03 | 3.34 | 3.81 | 2.47 | 2.53 | 3.41 | 3.11 |
| G79 | 68.35 | 69.42 | 65.17 | 64.40 | 71.82 | 67.83 | 7.45 | 7.51 | 6.12 | 8.00 | 6.94 | 7.20 | 4.27 | 4.78 | 3.13 | 4.23 | 4.16 | 4.12 |
| G80 | 61.53 | 65.05 | 60.10 | 61.41 | 61.39 | 61.90 | 7.08 | 7.90 | 6.34 | 6.90 | 6.86 | 7.01 | 3.93 | 4.67 | 3.03 | 3.65 | 3.49 | 3.76 |
| G81 | 60.35 | 66.84 | 57.88 | 64.73 | 63.48 | 62.66 | 6.36 | 6.45 | 5.48 | 5.30 | 6.59 | 6.04 | 2.57 | 2.96 | 1.99 | 2.50 | 2.98 | 2.60 |
| G82 | 60.63 | 63.39 | 61.87 | 59.73 | 57.06 | 60.54 | 7.44 | 8.13 | 7.44 | 7.30 | 7.08 | 7.48 | 3.66 | 4.25 | 4.04 | 3.16 | 3.45 | 3.71 |
| G83 | 54.02 | 57.09 | 53.11 | 51.62 | 58.73 | 54.91 | 5.56 | 5.65 | 5.82 | 5.31 | 7.05 | 5.88 | 2.85 | 3.09 | 2.69 | 2.33 | 4.22 | 3.04 |
| G84 | 69.94 | 72.59 | 71.07 | 66.75 | 72.75 | 70.62 | 8.51 | 8.22 | 8.07 | 7.23 | 7.86 | 7.98 | 5.41 | 5.43 | 4.78 | 4.08 | 5.84 | 5.11 |
| G85 | 60.17 | 62.39 | 57.52 | 58.65 | 65.89 | 60.92 | 6.52 | 7.37 | 6.46 | 6.67 | 6.61 | 6.72 | 3.61 | 4.30 | 2.97 | 3.58 | 4.25 | 3.74 |
| G86 | 53.44 | 52.25 | 47.23 | 49.18 | 57.50 | 51.92 | 7.42 | 7.41 | 7.02 | 7.67 | 7.06 | 7.32 | 3.43 | 3.35 | 2.58 | 3.02 | 3.77 | 3.23 |
| G87 | 49.81 | 55.78 | 46.37 | 49.74 | 54.71 | 51.28 | 6.27 | 6.56 | 6.36 | 5.69 | 6.54 | 6.28 | 3.04 | 3.76 | 2.73 | 2.49 | 3.82 | 3.17 |
| G88 | 51.74 | 59.16 | 48.26 | 52.80 | 56.39 | 53.67 | 6.18 | 6.38 | 6.80 | 6.13 | 6.27 | 6.35 | 2.70 | 3.21 | 2.87 | 2.70 | 3.25 | 2.95 |
| G89 | 63.23 | 68.71 | 60.53 | 66.29 | 65.57 | 64.87 | 7.89 | 8.35 | 7.33 | 7.99 | 7.72 | 7.86 | 4.29 | 5.39 | 3.43 | 4.43 | 4.52 | 4.41 |
| G90 | 62.66 | 66.31 | 62.76 | 67.44 | 65.48 | 64.93 | 8.69 | 8.89 | 7.53 | 8.86 | 8.28 | 8.45 | 5.54 | 6.00 | 4.11 | 5.40 | 5.34 | 5.28 |
| G91 | 61.55 | 62.98 | 58.07 | 62.95 | 67.17 | 62.55 | 7.40 | 8.98 | 6.96 | 8.42 | 7.83 | 7.92 | 3.99 | 4.90 | 3.40 | 4.90 | 5.20 | 4.48 |
| G92 | 53.83 | 58.81 | 57.18 | 53.78 | 61.43 | 57.01 | 5.88 | 6.05 | 6.17 | 5.58 | 6.30 | 6.00 | 2.02 | 2.47 | 2.69 | 1.77 | 2.92 | 2.37 |
| G93 | 55.35 | 56.91 | 52.63 | 53.68 | 58.03 | 55.32 | 6.26 | 7.24 | 4.74 | 5.60 | 6.87 | 6.14 | 3.19 | 3.79 | 1.87 | 2.42 | 3.80 | 3.01 |
| G94 | 46.01 | 57.25 | 45.17 | 53.38 | 59.54 | 52.27 | 5.89 | 5.80 | 5.19 | 5.44 | 6.04 | 5.67 | 2.40 | 3.05 | 1.86 | 2.37 | 3.33 | 2.60 |
| G95 | 66.51 | 62.95 | 64.72 | 67.37 | 72.57 | 66.82 | 7.34 | 8.94 | 5.99 | 8.29 | 8.17 | 7.74 | 4.11 | 4.98 | 2.98 | 4.44 | 5.06 | 4.32 |
| Mean | 60.72 | 65.15 | 61.06 | 60.19 | 65.37 | 62.50 | 7.03 | 7.38 | 6.61 | 6.91 | 7.02 | 6.99 | 3.68 | 4.19 | 3.38 | 3.46 | 3.58 | 3.66 |

Supplementary Table 2 Continued….

| GEN | SGT | | | | | | LSR | | | | | | NNP | | | | | |
| --- | --- | --- | --- | --- | --- | --- | --- | --- | --- | --- | --- | --- | --- | --- | --- | --- | --- | --- |
|  | E1 | E2 | E3 | E4 | E5 | AVG | E1 | E2 | E3 | E4 | E5 | AVG | E1 | E2 | E3 | E4 | E5 | AVG |
| G1 | 13.04 | 14.02 | 10.88 | 13.15 | 14.62 | 13.14 | 0.24 | 0.27 | 0.28 | 0.24 | 0.23 | 0.25 | 4.01 | 5.40 | 3.80 | 4.20 | 5.40 | 4.56 |
| G2 | 13.64 | 14.86 | 12.74 | 14.30 | 16.34 | 14.38 | 0.18 | 0.23 | 0.22 | 0.22 | 0.22 | 0.21 | 3.45 | 4.50 | 3.80 | 4.10 | 3.45 | 3.86 |
| G3 | 21.51 | 23.79 | 20.46 | 22.01 | 22.92 | 22.14 | 0.19 | 0.17 | 0.17 | 0.17 | 0.29 | 0.20 | 4.34 | 5.80 | 3.50 | 4.40 | 5.22 | 4.65 |
| G4 | 19.83 | 22.11 | 17.41 | 20.06 | 21.45 | 20.17 | 0.20 | 0.23 | 0.18 | 0.17 | 0.20 | 0.20 | 3.99 | 4.20 | 3.60 | 3.30 | 3.50 | 3.72 |
| G5 | 17.16 | 21.26 | 16.49 | 18.73 | 20.49 | 18.83 | 0.27 | 0.29 | 0.28 | 0.29 | 0.27 | 0.28 | 3.41 | 4.75 | 3.35 | 3.95 | 4.40 | 3.97 |
| G6 | 17.15 | 16.10 | 16.59 | 16.81 | 17.87 | 16.90 | 0.26 | 0.28 | 0.24 | 0.24 | 0.25 | 0.26 | 4.32 | 6.65 | 4.20 | 3.85 | 5.15 | 4.83 |
| G7 | 17.31 | 16.86 | 17.04 | 16.09 | 14.80 | 16.42 | 0.25 | 0.25 | 0.28 | 0.32 | 0.24 | 0.27 | 4.82 | 4.40 | 5.05 | 4.00 | 3.30 | 4.31 |
| G8 | 18.61 | 18.18 | 16.48 | 17.21 | 16.03 | 17.30 | 0.20 | 0.19 | 0.21 | 0.19 | 0.20 | 0.20 | 5.51 | 5.60 | 3.90 | 4.15 | 4.55 | 4.74 |
| G9 | 16.35 | 18.88 | 17.97 | 16.73 | 17.53 | 17.49 | 0.26 | 0.28 | 0.21 | 0.23 | 0.23 | 0.24 | 5.28 | 5.60 | 4.50 | 5.10 | 6.02 | 5.30 |
| G10 | 18.81 | 19.72 | 15.41 | 16.55 | 14.00 | 16.90 | 0.16 | 0.16 | 0.18 | 0.17 | 0.19 | 0.17 | 5.02 | 5.32 | 3.73 | 4.90 | 3.80 | 4.55 |
| G11 | 17.74 | 21.52 | 15.94 | 19.41 | 14.79 | 17.88 | 0.28 | 0.27 | 0.29 | 0.29 | 0.25 | 0.28 | 3.98 | 5.70 | 4.00 | 4.70 | 4.20 | 4.52 |
| G12 | 17.44 | 20.02 | 16.14 | 18.53 | 18.86 | 18.20 | 0.22 | 0.21 | 0.22 | 0.23 | 0.26 | 0.23 | 3.74 | 5.10 | 3.30 | 4.00 | 3.20 | 3.87 |
| G13 | 17.10 | 19.76 | 14.67 | 17.30 | 15.38 | 16.84 | 0.22 | 0.26 | 0.21 | 0.22 | 0.23 | 0.23 | 4.94 | 5.80 | 4.50 | 5.10 | 4.30 | 4.93 |
| G14 | 18.62 | 21.30 | 17.68 | 19.91 | 19.22 | 19.35 | 0.21 | 0.25 | 0.17 | 0.20 | 0.18 | 0.20 | 4.38 | 5.80 | 3.35 | 5.60 | 4.70 | 4.77 |
| G15 | 18.50 | 21.93 | 16.82 | 21.04 | 17.74 | 19.21 | 0.16 | 0.16 | 0.14 | 0.15 | 0.13 | 0.15 | 5.23 | 6.90 | 5.30 | 6.45 | 5.70 | 5.92 |
| G16 | 17.62 | 18.70 | 15.87 | 19.06 | 17.95 | 17.84 | 0.18 | 0.18 | 0.17 | 0.18 | 0.21 | 0.19 | 4.27 | 5.90 | 5.20 | 5.80 | 5.60 | 5.35 |
| G17 | 10.63 | 8.27 | 10.07 | 7.64 | 8.57 | 9.04 | 0.22 | 0.30 | 0.27 | 0.23 | 0.21 | 0.25 | 4.98 | 5.70 | 5.50 | 4.85 | 5.35 | 5.28 |
| G18 | 16.11 | 17.71 | 14.22 | 15.34 | 17.36 | 16.15 | 0.27 | 0.24 | 0.28 | 0.24 | 0.22 | 0.25 | 5.02 | 6.60 | 5.20 | 5.60 | 6.50 | 5.78 |
| G19 | 19.97 | 21.04 | 22.59 | 18.68 | 22.23 | 20.90 | 0.15 | 0.24 | 0.14 | 0.13 | 0.18 | 0.17 | 4.98 | 5.90 | 4.80 | 4.30 | 6.25 | 5.25 |
| G20 | 14.02 | 15.66 | 16.20 | 12.30 | 11.01 | 13.84 | 0.14 | 0.17 | 0.16 | 0.14 | 0.19 | 0.16 | 5.40 | 4.63 | 5.30 | 4.26 | 3.80 | 4.68 |
| G21 | 17.58 | 23.34 | 17.61 | 18.95 | 20.26 | 19.55 | 0.23 | 0.21 | 0.24 | 0.23 | 0.26 | 0.23 | 3.59 | 4.35 | 3.20 | 4.20 | 3.75 | 3.82 |
| G22 | 22.49 | 22.37 | 19.54 | 19.94 | 20.95 | 21.05 | 0.13 | 0.14 | 0.15 | 0.12 | 0.14 | 0.14 | 6.66 | 5.50 | 4.40 | 5.20 | 5.45 | 5.44 |
| G23 | 22.11 | 22.23 | 20.14 | 21.56 | 24.76 | 22.16 | 0.21 | 0.19 | 0.20 | 0.24 | 0.22 | 0.21 | 5.17 | 6.55 | 5.50 | 4.40 | 5.80 | 5.48 |
| G24 | 15.35 | 16.31 | 17.36 | 17.86 | 14.42 | 16.26 | 0.26 | 0.24 | 0.25 | 0.25 | 0.25 | 0.25 | 5.23 | 4.80 | 4.65 | 3.70 | 3.90 | 4.46 |
| G25 | 14.45 | 15.26 | 13.78 | 13.42 | 16.48 | 14.68 | 0.22 | 0.21 | 0.20 | 0.22 | 0.25 | 0.22 | 5.94 | 4.90 | 3.60 | 4.30 | 3.80 | 4.51 |
| G26 | 22.76 | 22.35 | 27.76 | 21.10 | 23.25 | 23.44 | 0.29 | 0.30 | 0.30 | 0.29 | 0.26 | 0.29 | 3.18 | 3.90 | 3.80 | 3.10 | 3.60 | 3.52 |
| G27 | 11.52 | 12.63 | 11.37 | 11.12 | 13.47 | 12.02 | 0.22 | 0.23 | 0.27 | 0.20 | 0.21 | 0.23 | 4.48 | 4.30 | 3.20 | 3.50 | 4.40 | 3.98 |
| G28 | 15.81 | 16.89 | 19.42 | 21.20 | 19.99 | 18.66 | 0.23 | 0.22 | 0.21 | 0.21 | 0.22 | 0.22 | 4.95 | 4.70 | 6.70 | 4.20 | 5.20 | 5.15 |
| G29 | 20.14 | 17.08 | 17.40 | 17.47 | 21.53 | 18.72 | 0.20 | 0.23 | 0.19 | 0.18 | 0.22 | 0.20 | 3.36 | 3.90 | 3.72 | 3.20 | 3.60 | 3.56 |
| G30 | 15.42 | 16.49 | 17.81 | 17.68 | 11.27 | 15.73 | 0.15 | 0.16 | 0.15 | 0.16 | 0.17 | 0.16 | 5.23 | 5.22 | 5.10 | 5.60 | 5.10 | 5.25 |
| G31 | 14.24 | 17.19 | 12.77 | 13.57 | 18.36 | 15.23 | 0.25 | 0.26 | 0.29 | 0.23 | 0.24 | 0.25 | 4.33 | 5.45 | 4.20 | 3.30 | 4.90 | 4.44 |
| G32 | 15.24 | 16.33 | 14.30 | 17.18 | 17.60 | 16.13 | 0.17 | 0.27 | 0.16 | 0.18 | 0.19 | 0.20 | 6.21 | 7.47 | 5.45 | 7.00 | 7.20 | 6.67 |
| G33 | 12.53 | 13.27 | 15.37 | 11.79 | 15.66 | 13.72 | 0.17 | 0.17 | 0.18 | 0.24 | 0.21 | 0.19 | 6.35 | 6.10 | 7.40 | 6.20 | 7.20 | 6.65 |
| G34 | 19.82 | 18.30 | 15.58 | 18.82 | 16.69 | 17.84 | 0.16 | 0.17 | 0.15 | 0.14 | 0.16 | 0.16 | 6.92 | 8.10 | 7.00 | 6.60 | 7.20 | 7.16 |
| G35 | 17.59 | 24.59 | 17.08 | 21.07 | 20.67 | 20.20 | 0.12 | 0.16 | 0.12 | 0.13 | 0.14 | 0.14 | 4.46 | 6.00 | 5.90 | 5.20 | 6.20 | 5.55 |
| G36 | 22.39 | 18.21 | 18.94 | 20.26 | 14.79 | 18.92 | 0.19 | 0.21 | 0.16 | 0.19 | 0.25 | 0.20 | 6.67 | 6.90 | 5.30 | 6.90 | 6.30 | 6.41 |
| G37 | 16.95 | 16.60 | 18.08 | 15.77 | 15.21 | 16.52 | 0.21 | 0.26 | 0.22 | 0.18 | 0.22 | 0.22 | 3.45 | 4.30 | 4.47 | 3.30 | 4.20 | 3.94 |
| G38 | 14.90 | 15.91 | 16.00 | 16.53 | 14.52 | 15.57 | 0.18 | 0.18 | 0.16 | 0.17 | 0.22 | 0.18 | 4.95 | 6.70 | 6.10 | 6.00 | 6.10 | 5.97 |
| G39 | 20.64 | 20.99 | 21.92 | 18.98 | 16.03 | 19.71 | 0.16 | 0.18 | 0.16 | 0.17 | 0.21 | 0.18 | 4.65 | 6.60 | 6.45 | 5.60 | 6.10 | 5.88 |
| G40 | 16.24 | 17.62 | 15.71 | 15.21 | 19.33 | 16.82 | 0.13 | 0.14 | 0.13 | 0.12 | 0.17 | 0.14 | 3.96 | 5.40 | 3.60 | 4.25 | 5.10 | 4.46 |
| G41 | 15.37 | 14.36 | 12.62 | 11.79 | 17.45 | 14.32 | 0.15 | 0.20 | 0.22 | 0.15 | 0.15 | 0.17 | 4.73 | 4.90 | 4.40 | 4.95 | 5.75 | 4.95 |
| G42 | 13.01 | 12.55 | 11.69 | 11.92 | 15.47 | 12.93 | 0.14 | 0.15 | 0.20 | 0.14 | 0.15 | 0.16 | 4.38 | 5.45 | 3.65 | 4.85 | 5.80 | 4.83 |
| G43 | 13.23 | 15.43 | 16.61 | 13.85 | 13.89 | 14.60 | 0.19 | 0.23 | 0.21 | 0.18 | 0.19 | 0.20 | 4.89 | 6.00 | 5.50 | 4.90 | 5.50 | 5.36 |
| G44 | 16.20 | 17.00 | 20.75 | 16.13 | 14.27 | 16.87 | 0.10 | 0.12 | 0.10 | 0.10 | 0.11 | 0.10 | 7.29 | 8.00 | 8.30 | 6.30 | 7.60 | 7.50 |
| G45 | 11.35 | 14.14 | 14.26 | 12.41 | 15.53 | 13.54 | 0.11 | 0.16 | 0.12 | 0.10 | 0.12 | 0.12 | 5.75 | 6.10 | 6.20 | 5.00 | 7.00 | 6.01 |
| G46 | 14.51 | 14.82 | 14.73 | 13.15 | 13.27 | 14.10 | 0.22 | 0.27 | 0.23 | 0.23 | 0.23 | 0.24 | 6.35 | 6.30 | 5.10 | 5.40 | 6.00 | 5.83 |
| G47 | 15.66 | 17.70 | 14.07 | 15.82 | 15.31 | 15.71 | 0.21 | 0.19 | 0.22 | 0.21 | 0.20 | 0.21 | 5.57 | 6.10 | 4.10 | 5.95 | 4.30 | 5.20 |
| G48 | 17.17 | 18.84 | 14.49 | 16.36 | 18.42 | 17.05 | 0.19 | 0.22 | 0.19 | 0.17 | 0.19 | 0.19 | 4.40 | 5.50 | 3.55 | 4.50 | 4.60 | 4.51 |
| G49 | 17.56 | 18.40 | 18.25 | 17.17 | 16.24 | 17.52 | 0.18 | 0.20 | 0.20 | 0.17 | 0.18 | 0.19 | 4.17 | 4.70 | 5.30 | 4.60 | 4.45 | 4.64 |
| G50 | 11.78 | 14.93 | 16.81 | 12.36 | 16.95 | 14.57 | 0.19 | 0.23 | 0.20 | 0.18 | 0.22 | 0.20 | 4.75 | 5.20 | 5.60 | 4.90 | 4.40 | 4.97 |
| G51 | 11.88 | 13.47 | 15.26 | 10.51 | 11.25 | 12.47 | 0.18 | 0.20 | 0.22 | 0.18 | 0.19 | 0.20 | 4.56 | 4.71 | 5.10 | 4.65 | 3.60 | 4.52 |
| G52 | 15.74 | 15.64 | 17.77 | 14.47 | 16.72 | 16.07 | 0.26 | 0.32 | 0.27 | 0.25 | 0.26 | 0.27 | 3.22 | 4.10 | 4.65 | 3.10 | 3.80 | 3.77 |
| G53 | 17.03 | 19.13 | 20.84 | 15.81 | 21.29 | 18.82 | 0.24 | 0.27 | 0.22 | 0.24 | 0.26 | 0.25 | 3.88 | 4.90 | 4.70 | 4.00 | 4.80 | 4.46 |
| G54 | 17.63 | 20.55 | 20.46 | 19.54 | 20.33 | 19.70 | 0.28 | 0.28 | 0.29 | 0.27 | 0.27 | 0.28 | 3.94 | 5.90 | 5.10 | 4.90 | 4.40 | 4.85 |
| G55 | 17.22 | 20.22 | 21.33 | 17.78 | 20.08 | 19.32 | 0.25 | 0.27 | 0.27 | 0.25 | 0.27 | 0.26 | 3.36 | 3.90 | 4.20 | 3.40 | 4.00 | 3.77 |
| G56 | 19.16 | 19.51 | 15.82 | 16.30 | 18.92 | 17.94 | 0.27 | 0.31 | 0.28 | 0.27 | 0.26 | 0.28 | 3.79 | 4.90 | 4.30 | 3.90 | 3.50 | 4.08 |
| G57 | 16.86 | 19.35 | 16.71 | 16.70 | 19.49 | 17.82 | 0.13 | 0.16 | 0.12 | 0.13 | 0.14 | 0.14 | 4.78 | 5.95 | 3.95 | 4.90 | 5.50 | 5.02 |
| G58 | 19.49 | 23.11 | 21.84 | 19.38 | 19.01 | 20.57 | 0.16 | 0.18 | 0.13 | 0.13 | 0.12 | 0.14 | 3.99 | 5.10 | 4.90 | 5.10 | 5.00 | 4.82 |
| G59 | 20.62 | 22.12 | 22.38 | 19.53 | 19.33 | 20.80 | 0.23 | 0.27 | 0.24 | 0.22 | 0.22 | 0.24 | 2.95 | 3.90 | 4.20 | 3.30 | 3.40 | 3.55 |
| G60 | 14.38 | 15.70 | 13.33 | 17.46 | 16.63 | 15.50 | 0.12 | 0.21 | 0.11 | 0.11 | 0.14 | 0.14 | 5.14 | 6.00 | 5.60 | 5.00 | 6.20 | 5.59 |
| G61 | 13.18 | 13.53 | 14.91 | 12.67 | 13.17 | 13.49 | 0.25 | 0.26 | 0.26 | 0.33 | 0.26 | 0.27 | 3.94 | 4.90 | 5.02 | 4.05 | 5.00 | 4.58 |
| G62 | 21.75 | 22.38 | 19.50 | 20.82 | 20.27 | 20.94 | 0.23 | 0.28 | 0.21 | 0.23 | 0.22 | 0.23 | 4.33 | 5.40 | 4.69 | 5.00 | 4.70 | 4.82 |
| G63 | 16.75 | 18.08 | 13.35 | 16.30 | 14.26 | 15.75 | 0.17 | 0.19 | 0.17 | 0.19 | 0.18 | 0.18 | 7.78 | 8.90 | 8.46 | 8.10 | 8.85 | 8.42 |
| G64 | 16.60 | 18.11 | 14.77 | 18.02 | 16.56 | 16.81 | 0.12 | 0.13 | 0.18 | 0.12 | 0.15 | 0.14 | 5.39 | 6.20 | 5.75 | 5.70 | 6.80 | 5.97 |
| G65 | 17.12 | 18.78 | 15.75 | 16.89 | 18.88 | 17.48 | 0.20 | 0.24 | 0.18 | 0.18 | 0.21 | 0.20 | 5.74 | 5.90 | 4.70 | 5.90 | 5.90 | 5.63 |
| G66 | 15.45 | 15.56 | 15.68 | 12.46 | 17.83 | 15.40 | 0.29 | 0.30 | 0.29 | 0.28 | 0.26 | 0.28 | 5.50 | 6.80 | 5.85 | 6.05 | 6.15 | 6.07 |
| G67 | 8.08 | 9.34 | 7.62 | 8.07 | 8.99 | 8.42 | 0.11 | 0.17 | 0.11 | 0.10 | 0.10 | 0.12 | 5.15 | 6.00 | 5.70 | 4.85 | 5.80 | 5.50 |
| G68 | 17.33 | 16.79 | 16.36 | 16.78 | 13.64 | 16.18 | 0.26 | 0.29 | 0.27 | 0.26 | 0.26 | 0.27 | 3.73 | 4.90 | 4.60 | 4.00 | 5.00 | 4.45 |
| G69 | 16.20 | 15.68 | 13.25 | 15.39 | 14.83 | 15.07 | 0.10 | 0.10 | 0.14 | 0.09 | 0.10 | 0.11 | 5.79 | 6.30 | 5.00 | 6.10 | 5.95 | 5.83 |
| G70 | 15.72 | 16.09 | 15.80 | 15.91 | 16.41 | 15.99 | 0.22 | 0.23 | 0.25 | 0.21 | 0.23 | 0.23 | 3.64 | 4.80 | 4.30 | 4.30 | 4.90 | 4.39 |
| G71 | 18.37 | 17.57 | 20.13 | 18.71 | 19.82 | 18.92 | 0.23 | 0.26 | 0.22 | 0.22 | 0.23 | 0.23 | 4.00 | 5.20 | 5.10 | 4.20 | 5.40 | 4.78 |
| G72 | 18.16 | 18.55 | 18.85 | 14.77 | 17.20 | 17.50 | 0.23 | 0.28 | 0.22 | 0.21 | 0.23 | 0.23 | 3.70 | 5.00 | 3.80 | 3.15 | 4.05 | 3.94 |
| G73 | 11.30 | 13.26 | 11.76 | 12.67 | 14.96 | 12.79 | 0.21 | 0.29 | 0.19 | 0.18 | 0.19 | 0.21 | 3.69 | 4.40 | 3.90 | 4.20 | 5.20 | 4.28 |
| G74 | 14.37 | 16.91 | 13.78 | 14.45 | 13.53 | 14.61 | 0.29 | 0.27 | 0.29 | 0.29 | 0.26 | 0.28 | 5.16 | 5.90 | 4.80 | 4.90 | 5.20 | 5.19 |
| G75 | 21.58 | 21.61 | 18.93 | 19.94 | 16.24 | 19.66 | 0.14 | 0.16 | 0.15 | 0.15 | 0.15 | 0.15 | 6.05 | 6.60 | 4.00 | 6.10 | 6.10 | 5.77 |
| G76 | 16.92 | 20.16 | 15.83 | 19.68 | 17.42 | 18.00 | 0.09 | 0.13 | 0.09 | 0.11 | 0.12 | 0.11 | 5.91 | 6.40 | 4.80 | 5.60 | 5.25 | 5.59 |
| G77 | 12.60 | 14.68 | 12.38 | 9.95 | 15.33 | 12.99 | 0.25 | 0.27 | 0.23 | 0.24 | 0.25 | 0.25 | 4.22 | 5.10 | 4.80 | 5.40 | 5.00 | 4.90 |
| G78 | 17.63 | 20.23 | 15.12 | 14.16 | 19.88 | 17.40 | 0.22 | 0.30 | 0.21 | 0.21 | 0.24 | 0.24 | 3.83 | 4.80 | 4.20 | 4.10 | 5.40 | 4.47 |
| G79 | 22.16 | 19.33 | 19.46 | 21.75 | 20.36 | 20.61 | 0.21 | 0.19 | 0.27 | 0.20 | 0.20 | 0.21 | 5.59 | 6.70 | 5.20 | 6.05 | 6.40 | 5.99 |
| G80 | 15.25 | 17.87 | 13.42 | 14.85 | 15.56 | 15.39 | 0.14 | 0.17 | 0.15 | 0.13 | 0.16 | 0.15 | 6.89 | 8.00 | 6.40 | 7.00 | 7.45 | 7.15 |
| G81 | 11.83 | 13.02 | 12.35 | 10.60 | 12.91 | 12.14 | 0.11 | 0.13 | 0.10 | 0.10 | 0.11 | 0.11 | 5.21 | 6.00 | 5.25 | 5.05 | 5.85 | 5.47 |
| G82 | 18.70 | 19.70 | 17.31 | 13.74 | 20.32 | 17.96 | 0.27 | 0.30 | 0.26 | 0.26 | 0.29 | 0.28 | 3.85 | 4.90 | 4.66 | 3.45 | 4.45 | 4.26 |
| G83 | 15.53 | 16.53 | 14.29 | 12.56 | 20.45 | 15.87 | 0.25 | 0.24 | 0.23 | 0.31 | 0.28 | 0.26 | 4.04 | 4.80 | 3.76 | 3.70 | 5.05 | 4.27 |
| G84 | 15.62 | 16.86 | 14.97 | 13.45 | 18.36 | 15.85 | 0.20 | 0.23 | 0.19 | 0.19 | 0.22 | 0.20 | 4.35 | 5.85 | 4.10 | 4.90 | 6.10 | 5.06 |
| G85 | 15.26 | 16.93 | 11.74 | 14.09 | 18.35 | 15.27 | 0.23 | 0.27 | 0.22 | 0.22 | 0.26 | 0.24 | 5.05 | 6.00 | 4.50 | 4.95 | 5.20 | 5.14 |
| G86 | 19.15 | 18.57 | 14.94 | 15.67 | 19.25 | 17.51 | 0.17 | 0.19 | 0.16 | 0.17 | 0.19 | 0.18 | 3.96 | 5.05 | 4.90 | 4.85 | 4.50 | 4.65 |
| G87 | 17.07 | 20.55 | 16.08 | 18.51 | 20.55 | 18.55 | 0.28 | 0.27 | 0.26 | 0.28 | 0.26 | 0.27 | 3.98 | 5.30 | 3.85 | 4.70 | 4.85 | 4.54 |
| G88 | 16.79 | 18.81 | 16.42 | 14.84 | 19.84 | 17.34 | 0.21 | 0.29 | 0.20 | 0.21 | 0.24 | 0.23 | 4.79 | 5.90 | 4.57 | 5.60 | 5.20 | 5.21 |
| G89 | 18.02 | 20.73 | 16.29 | 18.45 | 19.66 | 18.63 | 0.17 | 0.16 | 0.19 | 0.18 | 0.17 | 0.17 | 4.56 | 5.90 | 4.35 | 4.90 | 5.65 | 5.07 |
| G90 | 19.81 | 21.60 | 16.64 | 20.31 | 21.78 | 20.03 | 0.15 | 0.19 | 0.15 | 0.16 | 0.15 | 0.16 | 5.38 | 6.80 | 5.20 | 5.95 | 6.50 | 5.97 |
| G91 | 18.51 | 22.16 | 15.76 | 18.56 | 19.85 | 18.97 | 0.17 | 0.20 | 0.16 | 0.18 | 0.20 | 0.18 | 5.53 | 5.40 | 4.35 | 4.95 | 5.10 | 5.07 |
| G92 | 10.27 | 11.47 | 12.15 | 10.81 | 13.28 | 11.59 | 0.27 | 0.28 | 0.28 | 0.24 | 0.26 | 0.27 | 3.22 | 4.20 | 4.00 | 4.05 | 4.70 | 4.03 |
| G93 | 18.21 | 18.90 | 17.21 | 18.33 | 19.42 | 18.41 | 0.21 | 0.22 | 0.23 | 0.22 | 0.21 | 0.22 | 3.93 | 5.70 | 4.60 | 5.30 | 4.50 | 4.81 |
| G94 | 16.36 | 19.62 | 14.71 | 17.35 | 20.75 | 17.76 | 0.20 | 0.21 | 0.18 | 0.18 | 0.21 | 0.20 | 3.75 | 4.00 | 3.20 | 3.35 | 4.90 | 3.84 |
| G95 | 19.18 | 17.93 | 13.43 | 15.28 | 19.71 | 17.11 | 0.15 | 0.20 | 0.14 | 0.15 | 0.15 | 0.16 | 4.57 | 5.40 | 4.10 | 5.10 | 5.60 | 4.95 |
| Mean | 16.65 | 17.90 | 16.22 | 16.27 | 17.30 | 16.87 | 0.20 | 0.22 | 0.20 | 0.20 | 0.21 | 0.21 | 4.72 | 5.56 | 4.74 | 4.82 | 5.18 | 5.00 |

Supplementary Table 2 Continued….

| GEN | IL | | | | | | PL | | | | | | DMC | | | | | |
| --- | --- | --- | --- | --- | --- | --- | --- | --- | --- | --- | --- | --- | --- | --- | --- | --- | --- | --- |
|  | E1 | E2 | E3 | E4 | E5 | AVG | E1 | E2 | E3 | E4 | E5 | AVG | E1 | E2 | E3 | E4 | E5 | AVG |
| G1 | 6.18 | 7.31 | 6.71 | 6.18 | 7.43 | 6.76 | 21.08 | 22.21 | 18.10 | 18.71 | 21.06 | 20.23 | 26.01 | 33.50 | 30.44 | 32.70 | 26.84 | 29.90 |
| G2 | 13.18 | 16.68 | 15.18 | 15.83 | 7.04 | 13.58 | 21.76 | 23.01 | 21.01 | 20.97 | 21.87 | 21.72 | 28.88 | 32.81 | 31.11 | 29.82 | 30.66 | 30.66 |
| G3 | 7.29 | 11.04 | 7.27 | 6.77 | 6.51 | 7.78 | 29.64 | 30.69 | 26.19 | 28.11 | 27.84 | 28.49 | 31.36 | 31.53 | 29.58 | 30.99 | 30.39 | 30.77 |
| G4 | 7.81 | 10.59 | 8.13 | 7.51 | 8.82 | 8.57 | 27.18 | 28.29 | 23.35 | 24.14 | 27.38 | 26.07 | 26.01 | 28.41 | 33.58 | 26.33 | 28.83 | 28.63 |
| G5 | 6.42 | 8.80 | 6.99 | 6.58 | 8.02 | 7.36 | 26.77 | 28.40 | 24.48 | 25.52 | 27.22 | 26.48 | 29.79 | 32.56 | 29.74 | 31.88 | 29.04 | 30.60 |
| G6 | 8.76 | 9.22 | 6.86 | 6.28 | 7.41 | 7.71 | 24.90 | 27.13 | 24.07 | 22.90 | 26.11 | 25.02 | 22.87 | 24.36 | 28.21 | 24.44 | 28.52 | 25.68 |
| G7 | 9.90 | 9.74 | 10.95 | 9.04 | 6.41 | 9.21 | 26.59 | 27.98 | 26.80 | 24.24 | 26.96 | 26.51 | 23.02 | 24.53 | 21.01 | 22.04 | 28.13 | 23.75 |
| G8 | 10.42 | 11.04 | 8.46 | 9.97 | 9.30 | 9.84 | 24.43 | 23.29 | 23.53 | 24.34 | 24.22 | 23.96 | 25.78 | 26.78 | 32.96 | 26.84 | 20.22 | 26.52 |
| G9 | 15.75 | 16.61 | 14.17 | 15.93 | 19.70 | 16.43 | 22.57 | 22.65 | 21.39 | 19.97 | 23.43 | 22.00 | 30.91 | 27.68 | 27.56 | 28.15 | 25.18 | 27.89 |
| G10 | 9.93 | 12.98 | 8.68 | 9.20 | 9.05 | 9.97 | 22.73 | 25.43 | 20.78 | 25.41 | 24.76 | 23.82 | 32.42 | 34.47 | 26.09 | 29.65 | 20.12 | 28.55 |
| G11 | 7.05 | 7.90 | 6.68 | 6.95 | 5.90 | 6.89 | 25.90 | 25.63 | 21.00 | 23.40 | 23.87 | 23.96 | 25.15 | 29.57 | 27.79 | 25.96 | 29.54 | 27.60 |
| G12 | 7.61 | 8.51 | 6.68 | 7.05 | 4.94 | 6.96 | 27.48 | 30.29 | 24.40 | 25.58 | 22.74 | 26.10 | 26.76 | 28.15 | 31.69 | 27.47 | 28.60 | 28.54 |
| G13 | 7.20 | 8.44 | 7.55 | 7.55 | 6.79 | 7.50 | 22.07 | 24.15 | 19.73 | 20.00 | 20.51 | 21.29 | 32.43 | 32.37 | 30.89 | 29.25 | 29.79 | 30.95 |
| G14 | 10.53 | 13.65 | 11.25 | 12.22 | 8.15 | 11.16 | 29.54 | 32.73 | 28.39 | 33.71 | 30.61 | 31.00 | 28.15 | 30.40 | 33.39 | 29.32 | 30.59 | 30.37 |
| G15 | 10.96 | 13.65 | 12.59 | 13.64 | 5.16 | 11.20 | 24.02 | 26.78 | 22.11 | 28.01 | 23.97 | 24.97 | 30.54 | 34.17 | 32.36 | 32.30 | 29.23 | 31.72 |
| G16 | 6.95 | 9.98 | 8.56 | 8.92 | 6.39 | 8.16 | 25.62 | 28.88 | 23.44 | 30.15 | 26.72 | 26.96 | 31.19 | 34.23 | 32.13 | 32.39 | 31.25 | 32.24 |
| G17 | 30.06 | 35.84 | 31.42 | 35.28 | 22.10 | 30.94 | 39.95 | 38.59 | 36.84 | 38.11 | 41.56 | 39.01 | 29.60 | 30.33 | 30.20 | 29.55 | 30.18 | 29.97 |
| G18 | 11.92 | 14.05 | 11.11 | 12.11 | 14.78 | 12.80 | 22.04 | 23.22 | 19.75 | 20.29 | 22.14 | 21.49 | 34.31 | 34.07 | 28.73 | 29.78 | 32.15 | 31.81 |
| G19 | 13.37 | 9.50 | 9.31 | 8.15 | 12.61 | 10.59 | 27.79 | 30.28 | 25.48 | 25.66 | 29.48 | 27.74 | 30.95 | 30.08 | 31.21 | 29.59 | 27.00 | 29.76 |
| G20 | 15.05 | 14.96 | 17.30 | 12.94 | 8.23 | 13.70 | 27.94 | 27.84 | 30.23 | 25.51 | 24.82 | 27.27 | 33.97 | 28.58 | 31.48 | 27.68 | 32.24 | 30.79 |
| G21 | 10.17 | 13.53 | 9.20 | 10.98 | 12.66 | 11.31 | 23.57 | 26.99 | 23.88 | 20.31 | 27.38 | 24.43 | 27.10 | 27.02 | 30.95 | 28.57 | 28.87 | 28.50 |
| G22 | 9.85 | 10.44 | 8.96 | 8.35 | 12.76 | 10.07 | 29.27 | 30.86 | 28.27 | 27.15 | 29.10 | 28.93 | 30.93 | 33.32 | 31.06 | 32.29 | 29.02 | 31.32 |
| G23 | 7.73 | 8.36 | 7.89 | 7.78 | 8.88 | 8.13 | 21.21 | 23.04 | 21.28 | 22.20 | 9.83 | 19.51 | 24.18 | 22.42 | 29.12 | 23.17 | 22.91 | 24.36 |
| G24 | 9.90 | 13.19 | 8.87 | 9.84 | 8.21 | 10.00 | 27.03 | 29.23 | 27.10 | 27.85 | 28.02 | 27.85 | 32.76 | 28.09 | 28.41 | 29.05 | 28.98 | 29.46 |
| G25 | 11.57 | 11.83 | 11.94 | 11.65 | 9.82 | 11.36 | 26.79 | 29.04 | 21.57 | 22.92 | 25.82 | 25.23 | 24.98 | 24.72 | 30.63 | 29.57 | 31.40 | 28.26 |
| G26 | 7.00 | 8.98 | 12.71 | 7.71 | 8.44 | 8.97 | 25.01 | 31.95 | 31.00 | 25.80 | 32.50 | 29.25 | 26.23 | 23.79 | 28.42 | 26.05 | 27.74 | 26.45 |
| G27 | 8.06 | 8.28 | 7.15 | 9.67 | 6.96 | 8.02 | 26.50 | 25.36 | 26.00 | 21.80 | 24.94 | 24.92 | 26.52 | 27.55 | 26.29 | 23.04 | 31.46 | 26.97 |
| G28 | 8.50 | 9.61 | 11.24 | 8.85 | 8.94 | 9.43 | 24.09 | 26.47 | 29.34 | 24.84 | 27.41 | 26.43 | 27.89 | 29.41 | 26.37 | 30.65 | 26.29 | 28.12 |
| G29 | 7.10 | 8.23 | 10.47 | 6.72 | 7.59 | 8.02 | 20.09 | 22.53 | 19.20 | 19.43 | 22.30 | 20.71 | 33.38 | 32.70 | 28.27 | 32.18 | 22.14 | 29.73 |
| G30 | 13.54 | 17.56 | 15.55 | 18.53 | 14.70 | 15.97 | 26.53 | 27.39 | 24.13 | 27.62 | 23.38 | 25.81 | 30.19 | 32.25 | 30.03 | 29.73 | 31.30 | 30.70 |
| G31 | 10.60 | 10.82 | 9.52 | 10.07 | 11.24 | 10.45 | 33.54 | 34.73 | 29.56 | 30.52 | 35.51 | 32.77 | 23.56 | 26.57 | 24.53 | 24.13 | 30.24 | 25.80 |
| G32 | 14.51 | 15.22 | 11.08 | 14.96 | 15.22 | 14.20 | 24.49 | 27.92 | 22.39 | 30.72 | 24.82 | 26.07 | 29.67 | 30.54 | 32.02 | 28.76 | 27.17 | 29.63 |
| G33 | 15.80 | 15.95 | 18.50 | 16.04 | 17.98 | 16.85 | 24.24 | 26.60 | 27.24 | 21.61 | 30.62 | 26.06 | 23.81 | 26.28 | 31.60 | 23.90 | 30.25 | 27.17 |
| G34 | 18.85 | 19.73 | 19.00 | 20.22 | 11.65 | 17.89 | 23.07 | 24.56 | 21.57 | 21.48 | 22.33 | 22.60 | 31.95 | 29.87 | 28.63 | 30.26 | 26.64 | 29.47 |
| G35 | 18.25 | 21.09 | 18.22 | 18.55 | 17.96 | 18.81 | 19.66 | 21.05 | 27.15 | 21.19 | 20.15 | 21.84 | 24.85 | 24.57 | 22.81 | 24.25 | 25.54 | 24.41 |
| G36 | 17.40 | 20.04 | 19.98 | 20.28 | 17.74 | 19.09 | 26.14 | 29.67 | 24.79 | 29.28 | 27.34 | 27.44 | 25.64 | 25.74 | 26.23 | 25.59 | 28.05 | 26.25 |
| G37 | 14.14 | 14.71 | 16.80 | 13.63 | 10.89 | 14.03 | 23.74 | 27.54 | 24.15 | 20.65 | 26.36 | 24.49 | 25.27 | 25.87 | 27.63 | 25.53 | 26.63 | 26.19 |
| G38 | 19.30 | 22.59 | 16.80 | 20.16 | 13.98 | 18.57 | 23.78 | 27.16 | 28.78 | 27.83 | 23.73 | 26.26 | 30.18 | 30.21 | 32.59 | 28.92 | 28.21 | 30.02 |
| G39 | 13.93 | 14.99 | 16.80 | 14.11 | 11.86 | 14.34 | 26.59 | 28.81 | 24.34 | 27.97 | 23.36 | 26.21 | 30.79 | 33.28 | 32.41 | 30.21 | 31.21 | 31.58 |
| G40 | 12.35 | 13.91 | 13.01 | 17.96 | 13.86 | 14.22 | 24.94 | 27.15 | 25.70 | 24.11 | 25.88 | 25.56 | 31.78 | 27.51 | 30.26 | 27.91 | 28.07 | 29.10 |
| G41 | 9.40 | 11.06 | 9.25 | 9.68 | 15.29 | 10.93 | 23.70 | 25.71 | 20.33 | 18.55 | 26.31 | 22.92 | 31.02 | 34.12 | 28.86 | 32.06 | 31.89 | 31.59 |
| G42 | 10.20 | 13.29 | 11.20 | 13.97 | 12.73 | 12.28 | 21.46 | 21.06 | 15.56 | 14.45 | 21.69 | 18.84 | 33.02 | 33.52 | 28.27 | 31.81 | 31.22 | 31.57 |
| G43 | 11.25 | 13.74 | 16.04 | 13.42 | 8.00 | 12.49 | 23.89 | 23.27 | 25.20 | 23.36 | 24.23 | 23.99 | 31.16 | 26.31 | 29.11 | 25.81 | 23.61 | 27.20 |
| G44 | 22.25 | 25.24 | 21.85 | 26.01 | 15.52 | 22.17 | 28.71 | 28.59 | 30.47 | 23.16 | 27.39 | 27.66 | 33.72 | 33.44 | 30.90 | 32.50 | 31.30 | 32.37 |
| G45 | 23.83 | 22.31 | 22.37 | 23.34 | 25.00 | 23.37 | 29.75 | 30.32 | 31.04 | 28.90 | 31.32 | 30.26 | 34.05 | 31.01 | 32.19 | 32.62 | 30.23 | 32.02 |
| G46 | 16.73 | 16.30 | 14.02 | 13.94 | 17.87 | 15.77 | 16.75 | 18.38 | 15.90 | 13.65 | 16.33 | 16.20 | 30.58 | 26.89 | 31.88 | 29.52 | 28.35 | 29.44 |
| G47 | 14.65 | 16.03 | 11.67 | 18.68 | 15.80 | 15.36 | 24.74 | 27.81 | 26.12 | 30.88 | 25.84 | 27.08 | 19.59 | 19.87 | 20.62 | 20.81 | 18.38 | 19.86 |
| G48 | 11.15 | 12.55 | 11.86 | 8.92 | 7.91 | 10.48 | 24.33 | 28.78 | 28.07 | 23.34 | 24.69 | 25.84 | 26.15 | 30.33 | 31.29 | 26.68 | 27.70 | 28.43 |
| G49 | 9.90 | 8.84 | 13.94 | 10.16 | 10.95 | 10.76 | 22.06 | 24.55 | 26.96 | 21.72 | 26.06 | 24.27 | 34.31 | 32.83 | 24.89 | 32.82 | 31.17 | 31.21 |
| G50 | 13.18 | 15.63 | 15.17 | 11.87 | 10.81 | 13.33 | 25.44 | 23.93 | 20.45 | 20.57 | 24.55 | 22.99 | 24.50 | 24.97 | 24.90 | 22.44 | 22.61 | 23.88 |
| G51 | 10.31 | 12.45 | 11.94 | 10.83 | 9.01 | 10.91 | 21.63 | 24.13 | 25.14 | 20.38 | 21.30 | 22.52 | 26.15 | 26.41 | 23.92 | 27.37 | 28.72 | 26.51 |
| G52 | 6.03 | 5.48 | 5.49 | 4.26 | 4.77 | 5.21 | 27.18 | 29.76 | 31.73 | 24.26 | 28.58 | 28.30 | 26.90 | 25.01 | 25.90 | 25.77 | 29.73 | 26.66 |
| G53 | 13.99 | 15.23 | 14.55 | 11.20 | 9.81 | 12.96 | 22.96 | 27.13 | 27.09 | 21.06 | 26.62 | 24.97 | 31.57 | 29.91 | 30.51 | 26.19 | 30.16 | 29.67 |
| G54 | 7.99 | 10.52 | 14.04 | 9.22 | 6.77 | 9.71 | 22.43 | 24.83 | 26.07 | 20.27 | 23.38 | 23.39 | 29.43 | 26.24 | 28.04 | 22.33 | 24.67 | 26.14 |
| G55 | 6.83 | 8.54 | 7.02 | 6.97 | 6.82 | 7.24 | 24.85 | 30.07 | 29.76 | 25.91 | 31.32 | 28.38 | 23.03 | 28.02 | 27.04 | 24.50 | 31.73 | 26.87 |
| G56 | 15.02 | 16.73 | 15.62 | 15.48 | 13.40 | 15.25 | 23.85 | 25.32 | 22.52 | 21.93 | 24.06 | 23.53 | 27.64 | 25.30 | 23.23 | 23.59 | 27.33 | 25.42 |
| G57 | 8.15 | 9.94 | 7.76 | 8.46 | 8.53 | 8.57 | 19.45 | 20.49 | 16.74 | 17.73 | 18.73 | 18.63 | 29.70 | 33.63 | 16.84 | 30.02 | 30.18 | 28.07 |
| G58 | 8.92 | 12.02 | 8.17 | 10.24 | 8.98 | 9.67 | 21.79 | 22.91 | 23.94 | 22.96 | 11.59 | 22.81 | 34.12 | 32.89 | 31.02 | 31.67 | 28.44 | 31.63 |
| G59 | 5.08 | 6.55 | 5.70 | 6.11 | 7.83 | 6.25 | 32.02 | 33.52 | 34.49 | 30.82 | 30.12 | 32.19 | 28.27 | 26.13 | 33.25 | 25.01 | 26.81 | 27.90 |
| G60 | 15.76 | 17.54 | 11.67 | 15.87 | 15.12 | 15.19 | 28.45 | 31.33 | 26.48 | 26.62 | 30.03 | 28.58 | 29.93 | 33.77 | 29.68 | 32.57 | 31.97 | 31.58 |
| G61 | 9.20 | 10.36 | 10.56 | 8.97 | 6.51 | 9.12 | 23.99 | 26.34 | 24.13 | 24.49 | 24.76 | 24.74 | 26.59 | 23.89 | 23.68 | 25.05 | 26.61 | 25.17 |
| G62 | 8.96 | 11.34 | 7.98 | 9.84 | 7.16 | 9.05 | 24.91 | 25.16 | 26.57 | 28.92 | 27.22 | 26.56 | 30.88 | 32.38 | 31.19 | 29.25 | 27.82 | 30.30 |
| G63 | 19.07 | 20.46 | 15.83 | 17.96 | 11.06 | 16.88 | 25.24 | 23.68 | 23.67 | 26.51 | 24.80 | 24.78 | 27.83 | 27.99 | 26.34 | 23.42 | 26.73 | 26.46 |
| G64 | 15.85 | 20.87 | 13.86 | 16.19 | 15.35 | 16.42 | 23.94 | 22.97 | 21.66 | 25.33 | 21.98 | 23.18 | 28.65 | 32.58 | 32.09 | 33.04 | 30.17 | 31.31 |
| G65 | 9.21 | 10.25 | 12.97 | 9.03 | 10.01 | 10.30 | 24.31 | 25.28 | 23.62 | 22.87 | 25.57 | 24.33 | 28.38 | 29.18 | 28.75 | 26.47 | 27.96 | 28.15 |
| G66 | 14.08 | 14.94 | 17.41 | 14.69 | 16.40 | 15.50 | 24.33 | 25.82 | 23.93 | 21.28 | 27.94 | 24.66 | 28.48 | 27.09 | 27.68 | 26.08 | 27.84 | 27.43 |
| G67 | 26.95 | 30.88 | 27.35 | 29.27 | 28.82 | 28.65 | 29.08 | 30.36 | 26.69 | 26.30 | 28.30 | 28.15 | 30.87 | 31.02 | 32.33 | 27.41 | 29.59 | 30.24 |
| G68 | 5.72 | 10.03 | 4.66 | 5.52 | 5.81 | 6.35 | 24.39 | 26.63 | 23.25 | 25.33 | 28.02 | 25.52 | 26.69 | 27.42 | 27.73 | 22.09 | 26.40 | 26.07 |
| G69 | 19.67 | 16.83 | 13.96 | 16.28 | 12.51 | 15.85 | 19.28 | 21.45 | 18.75 | 22.24 | 22.78 | 20.90 | 29.23 | 34.80 | 31.57 | 32.11 | 31.89 | 31.92 |
| G70 | 9.01 | 14.90 | 8.26 | 9.34 | 9.57 | 10.21 | 22.76 | 25.53 | 21.73 | 23.82 | 23.63 | 23.50 | 28.04 | 27.55 | 31.96 | 27.60 | 29.98 | 29.03 |
| G71 | 8.11 | 8.82 | 9.62 | 7.85 | 6.97 | 8.27 | 21.75 | 22.38 | 21.40 | 20.35 | 23.29 | 21.83 | 30.75 | 31.40 | 22.83 | 30.86 | 27.97 | 28.76 |
| G72 | 6.25 | 7.48 | 12.05 | 5.97 | 9.91 | 8.33 | 21.93 | 24.27 | 21.30 | 22.82 | 22.86 | 22.63 | 27.17 | 30.46 | 25.74 | 27.60 | 27.08 | 27.61 |
| G73 | 11.72 | 15.54 | 12.85 | 13.89 | 15.60 | 13.92 | 17.99 | 19.45 | 17.78 | 16.74 | 17.41 | 17.87 | 31.82 | 34.06 | 31.11 | 27.87 | 31.83 | 31.34 |
| G74 | 11.41 | 11.93 | 9.92 | 9.48 | 11.32 | 10.81 | 21.75 | 22.64 | 19.51 | 20.90 | 22.91 | 21.54 | 33.03 | 32.54 | 30.45 | 28.12 | 26.98 | 30.22 |
| G75 | 9.04 | 9.82 | 9.31 | 10.08 | 12.38 | 10.13 | 20.53 | 23.18 | 21.53 | 23.91 | 22.19 | 22.27 | 31.03 | 32.60 | 32.67 | 32.30 | 29.65 | 31.65 |
| G76 | 9.36 | 11.26 | 6.50 | 7.95 | 7.94 | 8.60 | 20.12 | 23.64 | 20.51 | 24.56 | 21.30 | 22.03 | 31.44 | 32.39 | 32.86 | 29.19 | 31.15 | 31.41 |
| G77 | 4.30 | 6.41 | 4.27 | 5.91 | 5.84 | 5.35 | 17.82 | 19.58 | 19.92 | 19.52 | 20.75 | 19.52 | 28.26 | 27.59 | 23.43 | 24.48 | 24.55 | 25.66 |
| G78 | 5.48 | 8.02 | 5.94 | 6.96 | 10.58 | 7.39 | 25.46 | 26.80 | 23.55 | 26.64 | 29.61 | 26.41 | 23.79 | 25.54 | 26.99 | 24.36 | 26.48 | 25.43 |
| G79 | 4.63 | 8.01 | 7.28 | 6.84 | 9.24 | 7.20 | 19.42 | 21.67 | 18.61 | 20.45 | 18.58 | 19.74 | 25.44 | 26.37 | 20.40 | 22.40 | 28.45 | 24.61 |
| G80 | 17.68 | 20.86 | 16.01 | 16.42 | 17.46 | 17.68 | 19.66 | 23.18 | 18.25 | 19.62 | 21.96 | 20.53 | 30.46 | 29.93 | 28.63 | 30.01 | 28.95 | 29.60 |
| G81 | 23.09 | 24.81 | 23.14 | 23.13 | 26.77 | 24.18 | 29.06 | 31.51 | 27.94 | 27.46 | 30.18 | 29.23 | 26.45 | 32.94 | 33.22 | 32.18 | 31.67 | 31.29 |
| G82 | 15.90 | 18.94 | 15.99 | 15.05 | 16.19 | 16.41 | 23.97 | 25.50 | 23.68 | 19.59 | 24.50 | 23.45 | 24.49 | 30.72 | 30.20 | 24.91 | 25.62 | 27.19 |
| G83 | 8.30 | 10.83 | 8.76 | 7.81 | 12.07 | 9.55 | 27.92 | 29.05 | 24.43 | 25.58 | 29.15 | 27.23 | 28.35 | 32.16 | 28.92 | 30.16 | 29.55 | 29.83 |
| G84 | 10.08 | 11.16 | 9.71 | 10.07 | 12.93 | 10.79 | 23.32 | 25.87 | 23.56 | 20.06 | 26.67 | 23.90 | 28.54 | 28.08 | 28.41 | 26.23 | 27.38 | 27.73 |
| G85 | 15.85 | 17.46 | 13.58 | 16.06 | 20.54 | 16.70 | 19.26 | 25.57 | 19.70 | 19.73 | 24.51 | 21.75 | 28.44 | 28.17 | 26.65 | 27.63 | 27.69 | 27.72 |
| G86 | 8.35 | 11.33 | 8.64 | 9.93 | 11.80 | 10.01 | 23.01 | 25.01 | 19.68 | 25.36 | 26.28 | 23.87 | 31.21 | 33.32 | 32.45 | 33.38 | 34.78 | 33.03 |
| G87 | 7.07 | 8.32 | 7.08 | 6.31 | 9.80 | 7.72 | 23.88 | 26.70 | 20.12 | 21.62 | 28.29 | 24.12 | 24.77 | 25.47 | 20.10 | 23.55 | 25.81 | 23.94 |
| G88 | 7.12 | 8.44 | 7.77 | 7.11 | 10.58 | 8.21 | 21.86 | 24.46 | 19.48 | 19.92 | 25.23 | 22.19 | 31.32 | 31.88 | 29.94 | 25.49 | 33.47 | 30.42 |
| G89 | 10.66 | 12.06 | 11.02 | 12.24 | 13.20 | 11.84 | 31.07 | 34.93 | 28.45 | 34.07 | 30.91 | 31.89 | 29.38 | 28.10 | 26.53 | 25.92 | 27.98 | 27.58 |
| G90 | 14.20 | 15.56 | 12.78 | 13.72 | 13.04 | 13.86 | 23.87 | 26.86 | 22.89 | 26.58 | 24.69 | 24.98 | 33.61 | 32.87 | 29.04 | 29.91 | 31.51 | 31.39 |
| G91 | 7.69 | 9.96 | 7.76 | 9.91 | 11.90 | 9.44 | 25.36 | 28.64 | 22.46 | 28.88 | 29.85 | 27.04 | 32.15 | 32.11 | 28.89 | 31.35 | 32.39 | 31.38 |
| G92 | 7.28 | 8.35 | 7.74 | 6.80 | 11.00 | 8.23 | 22.16 | 23.99 | 24.37 | 22.34 | 24.00 | 23.37 | 29.53 | 30.08 | 24.02 | 29.23 | 31.10 | 28.79 |
| G93 | 9.00 | 10.17 | 9.30 | 8.95 | 11.58 | 9.80 | 22.99 | 25.54 | 22.01 | 22.52 | 24.16 | 23.44 | 26.87 | 29.96 | 22.39 | 23.83 | 29.56 | 26.52 |
| G94 | 6.77 | 7.76 | 7.40 | 6.79 | 10.95 | 7.93 | 19.90 | 21.52 | 15.99 | 16.58 | 22.09 | 19.22 | 31.02 | 30.08 | 26.91 | 23.65 | 28.23 | 27.98 |
| G95 | 20.61 | 16.90 | 17.75 | 18.96 | 20.15 | 18.87 | 24.65 | 26.11 | 21.91 | 28.53 | 26.97 | 25.63 | 32.16 | 31.93 | 30.72 | 30.45 | 30.77 | 31.21 |
| Mean | 11.61 | 13.27 | 11.83 | 11.94 | 11.58 | 12.05 | 24.38 | 26.30 | 23.85 | 23.91 | 25.22 | 24.76 | 28.88 | 29.53 | 28.61 | 27.93 | 28.55 | 28.70 |

Supplementary Table 2 Continued….

| GEN | DFYP | | | | | | GFYP | | | | | |
| --- | --- | --- | --- | --- | --- | --- | --- | --- | --- | --- | --- | --- |
|  | E1 | E2 | E3 | E4 | E5 | AVG | E1 | E2 | E3 | E4 | E5 | AVG |
| G1 | 35.62 | 46.24 | 33.10 | 43.00 | 52.96 | 42.58 | 136.95 | 137.92 | 108.53 | 131.68 | 196.70 | 142.36 |
| G2 | 66.42 | 81.15 | 65.02 | 62.71 | 39.22 | 65.51 | 229.54 | 247.59 | 208.95 | 210.09 | 127.93 | 204.82 |
| G3 | 107.84 | 108.85 | 75.98 | 107.52 | 82.33 | 96.50 | 343.66 | 344.95 | 258.26 | 347.51 | 270.61 | 313.00 |
| G4 | 91.89 | 97.53 | 62.14 | 89.19 | 80.57 | 84.46 | 368.02 | 343.10 | 184.59 | 338.22 | 278.50 | 302.48 |
| G5 | 49.57 | 54.94 | 36.21 | 49.93 | 47.37 | 47.60 | 167.13 | 169.25 | 122.35 | 156.67 | 163.01 | 155.68 |
| G6 | 52.96 | 51.50 | 35.38 | 49.73 | 55.04 | 49.32 | 232.58 | 210.74 | 125.56 | 203.39 | 193.17 | 193.09 |
| G7 | 53.66 | 58.87 | 76.99 | 46.47 | 51.64 | 57.53 | 233.14 | 240.15 | 367.08 | 210.54 | 183.24 | 246.83 |
| G8 | 86.04 | 92.65 | 52.29 | 84.72 | 56.08 | 75.45 | 334.01 | 345.90 | 158.19 | 316.64 | 276.65 | 286.28 |
| G9 | 63.83 | 66.31 | 38.69 | 55.02 | 89.59 | 62.69 | 209.20 | 239.89 | 141.14 | 195.65 | 355.29 | 228.23 |
| G10 | 126.37 | 140.82 | 68.08 | 119.12 | 45.36 | 100.35 | 389.85 | 408.87 | 260.64 | 401.42 | 224.86 | 337.13 |
| G11 | 55.78 | 60.22 | 50.00 | 51.57 | 49.43 | 53.40 | 222.74 | 203.13 | 180.10 | 199.05 | 167.61 | 194.53 |
| G12 | 42.82 | 41.64 | 38.41 | 41.91 | 30.27 | 39.01 | 160.05 | 147.77 | 121.28 | 153.03 | 105.37 | 137.50 |
| G13 | 72.82 | 76.98 | 56.65 | 69.31 | 57.61 | 68.87 | 225.18 | 237.48 | 183.51 | 236.47 | 192.98 | 215.12 |
| G14 | 107.08 | 119.41 | 90.56 | 109.63 | 53.08 | 96.95 | 379.49 | 392.56 | 270.60 | 373.81 | 173.00 | 317.89 |
| G15 | 110.25 | 128.87 | 89.00 | 117.00 | 56.55 | 101.73 | 362.25 | 376.78 | 275.26 | 363.96 | 193.37 | 314.33 |
| G16 | 117.19 | 126.44 | 89.43 | 117.41 | 57.83 | 101.96 | 376.06 | 369.75 | 278.00 | 362.14 | 185.15 | 314.22 |
| G17 | 52.39 | 27.15 | 26.56 | 23.51 | 31.48 | 32.22 | 177.79 | 90.26 | 87.69 | 80.60 | 104.46 | 108.16 |
| G18 | 98.43 | 86.97 | 51.20 | 75.10 | 118.05 | 86.35 | 286.51 | 255.70 | 177.48 | 252.11 | 367.48 | 267.86 |
| G19 | 94.91 | 44.21 | 53.05 | 37.32 | 81.97 | 62.29 | 307.53 | 145.51 | 171.32 | 125.08 | 306.64 | 211.21 |
| G20 | 64.80 | 41.50 | 90.41 | 34.80 | 32.24 | 53.15 | 191.90 | 145.20 | 286.89 | 125.92 | 99.74 | 169.93 |
| G21 | 67.66 | 72.62 | 48.96 | 67.71 | 57.97 | 62.99 | 249.70 | 269.53 | 157.85 | 236.74 | 200.78 | 222.92 |
| G22 | 105.51 | 117.95 | 82.46 | 109.74 | 85.99 | 101.33 | 341.23 | 354.37 | 266.24 | 339.27 | 295.19 | 319.26 |
| G23 | 66.56 | 67.05 | 79.10 | 61.99 | 69.66 | 68.87 | 275.40 | 299.08 | 270.86 | 267.39 | 303.69 | 283.28 |
| G24 | 77.36 | 71.75 | 62.18 | 65.27 | 56.74 | 66.66 | 236.18 | 254.64 | 218.25 | 224.78 | 195.84 | 225.94 |
| G25 | 81.39 | 68.93 | 60.97 | 86.29 | 58.49 | 71.51 | 325.38 | 278.81 | 198.36 | 292.48 | 185.62 | 256.13 |
| G26 | 55.00 | 50.11 | 66.72 | 46.17 | 52.20 | 54.04 | 209.63 | 210.44 | 235.06 | 176.71 | 188.75 | 204.12 |
| G27 | 29.60 | 27.21 | 21.99 | 22.99 | 58.90 | 32.14 | 111.88 | 99.45 | 83.32 | 100.15 | 187.17 | 116.39 |
| G28 | 71.90 | 80.55 | 82.34 | 76.99 | 63.39 | 75.53 | 257.89 | 273.46 | 311.49 | 250.67 | 240.61 | 266.82 |
| G29 | 53.16 | 46.93 | 44.41 | 44.88 | 33.90 | 45.46 | 159.27 | 142.97 | 157.52 | 139.11 | 153.07 | 150.39 |
| G30 | 112.27 | 122.75 | 112.74 | 112.24 | 55.25 | 104.85 | 373.09 | 381.02 | 375.26 | 378.02 | 176.45 | 336.77 |
| G31 | 60.51 | 58.12 | 48.47 | 47.15 | 86.29 | 60.11 | 257.98 | 218.95 | 197.50 | 195.30 | 285.20 | 230.98 |
| G32 | 112.85 | 125.41 | 66.95 | 107.95 | 94.45 | 101.52 | 380.19 | 409.54 | 208.62 | 375.09 | 346.79 | 344.05 |
| G33 | 81.35 | 78.01 | 116.29 | 67.07 | 107.09 | 89.96 | 342.06 | 296.55 | 367.63 | 280.76 | 353.21 | 328.04 |
| G34 | 102.01 | 94.00 | 91.86 | 87.70 | 76.39 | 91.09 | 319.32 | 314.16 | 320.45 | 289.78 | 287.79 | 306.30 |
| G35 | 121.23 | 119.45 | 111.13 | 120.09 | 70.31 | 108.94 | 487.36 | 487.45 | 486.63 | 494.87 | 274.58 | 446.18 |
| G36 | 120.79 | 109.24 | 104.54 | 113.41 | 83.01 | 106.20 | 471.15 | 425.62 | 398.28 | 442.80 | 296.01 | 406.77 |
| G37 | 59.44 | 55.24 | 82.87 | 38.29 | 60.78 | 59.73 | 235.97 | 213.44 | 300.12 | 150.31 | 227.65 | 225.50 |
| G38 | 122.22 | 123.91 | 128.31 | 122.79 | 79.59 | 115.36 | 405.49 | 409.70 | 393.46 | 424.99 | 281.49 | 383.02 |
| G39 | 90.15 | 86.60 | 111.28 | 77.33 | 45.65 | 82.60 | 292.16 | 260.20 | 343.70 | 255.70 | 146.41 | 259.63 |
| G40 | 92.94 | 87.26 | 76.95 | 82.97 | 51.89 | 78.40 | 292.73 | 317.60 | 254.58 | 297.15 | 184.25 | 269.26 |
| G41 | 92.29 | 99.96 | 63.40 | 90.45 | 107.56 | 90.73 | 297.52 | 292.70 | 218.68 | 281.42 | 337.38 | 285.54 |
| G42 | 68.58 | 62.43 | 39.29 | 53.10 | 73.25 | 59.33 | 207.77 | 186.05 | 138.50 | 166.58 | 234.99 | 186.77 |
| G43 | 74.35 | 73.71 | 108.57 | 63.20 | 62.39 | 76.44 | 238.90 | 280.00 | 372.61 | 246.86 | 263.53 | 280.38 |
| G44 | 97.49 | 110.84 | 102.25 | 104.41 | 85.47 | 100.09 | 288.89 | 331.21 | 330.73 | 321.61 | 273.63 | 309.21 |
| G45 | 75.53 | 67.44 | 76.83 | 62.16 | 87.98 | 73.99 | 221.45 | 217.23 | 238.22 | 189.92 | 290.92 | 231.55 |
| G46 | 64.87 | 71.34 | 56.42 | 67.36 | 71.36 | 66.27 | 213.46 | 264.69 | 176.45 | 227.57 | 251.12 | 226.66 |
| G47 | 73.87 | 79.71 | 62.40 | 78.16 | 64.65 | 72.26 | 376.46 | 400.71 | 304.39 | 378.27 | 351.60 | 362.28 |
| G48 | 63.92 | 66.81 | 53.71 | 54.65 | 48.48 | 57.51 | 245.18 | 220.21 | 171.37 | 204.83 | 174.28 | 203.17 |
| G49 | 111.75 | 116.63 | 118.05 | 106.49 | 86.90 | 108.37 | 325.48 | 355.00 | 475.11 | 324.88 | 278.51 | 351.79 |
| G50 | 47.98 | 44.94 | 68.87 | 38.76 | 42.50 | 48.61 | 195.67 | 180.05 | 276.23 | 172.14 | 187.25 | 202.27 |
| G51 | 47.92 | 54.95 | 64.66 | 47.66 | 32.04 | 49.95 | 183.41 | 208.13 | 270.89 | 173.52 | 111.18 | 189.43 |
| G52 | 65.83 | 69.72 | 75.37 | 62.71 | 59.35 | 66.60 | 244.94 | 277.62 | 291.28 | 242.65 | 199.11 | 251.12 |
| G53 | 68.87 | 63.67 | 100.67 | 49.33 | 59.67 | 68.94 | 217.91 | 212.75 | 328.90 | 187.81 | 197.67 | 229.01 |
| G54 | 87.63 | 82.99 | 121.04 | 68.85 | 68.66 | 85.83 | 297.20 | 316.41 | 431.34 | 309.95 | 277.51 | 326.48 |
| G55 | 45.00 | 49.13 | 65.02 | 34.89 | 67.97 | 52.80 | 195.55 | 175.18 | 239.61 | 141.75 | 213.84 | 193.19 |
| G56 | 68.98 | 71.99 | 55.93 | 61.68 | 76.37 | 66.99 | 250.17 | 283.90 | 240.91 | 261.16 | 279.63 | 263.15 |
| G57 | 79.48 | 96.41 | 45.68 | 79.07 | 62.17 | 72.56 | 266.71 | 286.65 | 269.81 | 262.89 | 205.74 | 258.36 |
| G58 | 147.51 | 136.77 | 121.01 | 130.37 | 103.26 | 127.78 | 432.54 | 416.08 | 388.76 | 411.17 | 361.98 | 402.11 |
| G59 | 72.15 | 65.85 | 92.54 | 54.48 | 53.93 | 67.79 | 257.17 | 251.91 | 278.10 | 217.91 | 200.56 | 241.13 |
| G60 | 73.63 | 103.93 | 67.04 | 92.45 | 123.46 | 101.00 | 250.00 | 307.90 | 225.60 | 283.34 | 385.99 | 290.57 |
| G61 | 43.57 | 37.68 | 64.08 | 30.94 | 34.49 | 42.15 | 164.94 | 157.35 | 270.35 | 123.20 | 129.34 | 169.04 |
| G62 | 123.69 | 129.68 | 109.84 | 111.69 | 87.16 | 112.41 | 400.49 | 400.08 | 352.29 | 381.71 | 313.68 | 369.65 |
| G63 | 127.36 | 137.98 | 118.07 | 108.58 | 100.03 | 118.41 | 459.35 | 492.62 | 448.33 | 463.36 | 373.91 | 447.51 |
| G64 | 120.62 | 145.92 | 76.06 | 130.01 | 97.59 | 114.44 | 421.38 | 447.64 | 237.49 | 393.18 | 323.44 | 364.63 |
| G65 | 79.25 | 78.58 | 95.37 | 67.52 | 83.47 | 80.84 | 279.31 | 268.81 | 331.51 | 255.34 | 298.08 | 286.61 |
| G66 | 86.06 | 83.98 | 109.30 | 81.54 | 90.80 | 90.34 | 302.40 | 309.55 | 394.51 | 311.84 | 325.39 | 328.74 |
| G67 | 29.09 | 32.66 | 27.30 | 25.85 | 39.76 | 31.03 | 94.26 | 105.05 | 84.68 | 93.88 | 134.60 | 102.50 |
| G68 | 56.30 | 62.08 | 45.69 | 47.10 | 41.18 | 50.47 | 210.77 | 227.42 | 164.93 | 213.69 | 155.64 | 194.49 |
| G69 | 157.12 | 173.72 | 93.28 | 156.34 | 118.77 | 139.84 | 536.22 | 498.37 | 294.96 | 486.94 | 372.29 | 437.76 |
| G70 | 74.11 | 73.97 | 60.38 | 65.79 | 61.45 | 67.14 | 264.44 | 267.96 | 189.46 | 238.30 | 204.99 | 233.03 |
| G71 | 51.70 | 55.67 | 65.59 | 45.97 | 54.06 | 54.60 | 168.30 | 177.97 | 287.21 | 149.46 | 193.56 | 195.30 |
| G72 | 46.52 | 56.78 | 63.18 | 46.51 | 55.74 | 53.75 | 171.10 | 186.52 | 244.77 | 167.96 | 205.13 | 195.09 |
| G73 | 49.61 | 59.06 | 45.22 | 41.48 | 67.05 | 52.48 | 155.74 | 173.06 | 145.52 | 148.84 | 210.46 | 166.72 |
| G74 | 53.29 | 57.26 | 50.60 | 41.49 | 33.53 | 48.73 | 161.28 | 175.56 | 164.87 | 148.87 | 124.30 | 154.98 |
| G75 | 115.41 | 122.10 | 89.72 | 110.12 | 60.52 | 99.57 | 371.66 | 374.35 | 274.36 | 342.67 | 203.68 | 313.34 |
| G76 | 108.69 | 113.15 | 59.28 | 101.52 | 65.33 | 89.59 | 345.57 | 348.35 | 180.33 | 347.39 | 209.50 | 286.23 |
| G77 | 42.60 | 50.09 | 32.82 | 30.93 | 60.77 | 43.64 | 150.54 | 180.84 | 139.60 | 126.29 | 248.11 | 169.08 |
| G78 | 45.96 | 51.88 | 39.83 | 32.31 | 54.29 | 44.95 | 198.42 | 202.46 | 147.62 | 132.31 | 204.68 | 177.10 |
| G79 | 70.65 | 78.03 | 47.47 | 55.56 | 73.20 | 64.98 | 277.68 | 295.36 | 232.48 | 247.98 | 257.18 | 262.14 |
| G80 | 98.23 | 108.96 | 86.03 | 100.77 | 87.47 | 96.29 | 323.36 | 364.11 | 300.38 | 335.71 | 302.19 | 325.15 |
| G81 | 60.19 | 69.67 | 61.83 | 57.79 | 61.86 | 62.27 | 226.67 | 210.87 | 186.23 | 180.80 | 195.17 | 199.95 |
| G82 | 66.40 | 85.53 | 82.40 | 61.22 | 61.07 | 71.73 | 271.57 | 277.67 | 272.33 | 245.13 | 238.00 | 260.94 |
| G83 | 63.25 | 71.04 | 58.26 | 55.43 | 69.31 | 63.46 | 223.71 | 220.25 | 201.43 | 184.18 | 235.10 | 212.93 |
| G84 | 90.40 | 91.11 | 84.18 | 79.53 | 88.42 | 86.73 | 316.79 | 323.97 | 296.04 | 302.93 | 323.32 | 312.61 |
| G85 | 61.36 | 66.28 | 50.85 | 57.58 | 64.62 | 60.14 | 215.68 | 235.25 | 190.85 | 208.58 | 233.82 | 216.83 |
| G86 | 119.62 | 133.05 | 99.35 | 130.19 | 141.51 | 128.84 | 383.46 | 398.65 | 305.66 | 389.70 | 407.23 | 376.94 |
| G87 | 54.77 | 55.94 | 42.15 | 45.43 | 59.46 | 51.55 | 222.11 | 219.44 | 209.87 | 193.37 | 230.49 | 215.06 |
| G88 | 77.10 | 70.01 | 66.06 | 54.48 | 77.90 | 69.11 | 247.02 | 219.94 | 220.93 | 213.77 | 232.92 | 226.92 |
| G89 | 119.61 | 113.59 | 97.61 | 97.61 | 112.62 | 108.71 | 407.06 | 404.06 | 368.33 | 376.25 | 402.80 | 391.70 |
| G90 | 129.99 | 129.00 | 109.03 | 105.55 | 115.64 | 117.84 | 386.87 | 392.14 | 376.58 | 352.53 | 367.37 | 375.10 |
| G91 | 112.58 | 118.33 | 88.35 | 105.78 | 118.48 | 108.70 | 350.15 | 369.08 | 309.63 | 337.12 | 365.73 | 346.34 |
| G92 | 33.07 | 27.55 | 29.06 | 24.90 | 51.71 | 33.26 | 113.30 | 91.64 | 120.73 | 85.02 | 166.07 | 115.35 |
| G93 | 71.58 | 81.43 | 54.67 | 60.84 | 89.46 | 71.60 | 266.74 | 271.73 | 243.75 | 255.03 | 303.63 | 268.18 |
| G94 | 49.38 | 50.13 | 36.42 | 32.56 | 48.90 | 43.48 | 160.68 | 166.31 | 135.95 | 137.61 | 172.77 | 154.67 |
| G95 | 119.29 | 119.69 | 111.06 | 115.18 | 123.22 | 117.99 | 371.46 | 375.07 | 360.90 | 378.22 | 400.22 | 377.17 |
| Mean | 79.69 | 82.06 | 71.46 | 72.21 | 69.52 | 75.37 | 275.62 | 276.45 | 251.21 | 256.33 | 243.34 | 260.59 |

[days to 50% flowering (FDF, in days), plant height at 50 % flowering (PH, in cm), number of leaves per plant (NLP), leaf length (LFL, in cm), leaf width (LFW, in cm), leaf area index (LAI), leaf to stem ratio (LSR), stem girth (SGT, in mm), number of nodes per plant (NNP), inter-nodal distance (IL, in cm), panicle length (PL, in cm), dry matter content (DMC, in %), dry fodder yield per plant (DFYP, in g) and green fodder yield per plant (GFYP, in g)]

Supplementary Table 3 Mean performances of 95 forage Sorghum genotypes for 14 adaptive traits across the five environments

| GEN | FDF | PH | NLP | LFL | LFW | LAI | SGT | LSR | NNP | IL | PL | DMC | DFYP | GFYP |
| --- | --- | --- | --- | --- | --- | --- | --- | --- | --- | --- | --- | --- | --- | --- |
| G1 | 65.70 | 118.59 | 9.13 | 57.69 | 5.46 | 2.537 | 13.14 | 0.252 | 4.56 | 6.76 | 20.23 | 29.90 | 42.18 | 142.36 |
| G2 | 71.80 | 116.67 | 8.44 | 64.07 | 7.92 | 3.540 | 14.38 | 0.212 | 3.86 | 13.58 | 21.72 | 30.66 | 62.91 | 204.82 |
| G3 | 69.20 | 132.13 | 9.77 | 68.31 | 7.96 | 4.523 | 22.14 | 0.199 | 4.65 | 7.78 | 28.49 | 30.77 | 96.50 | 313.00 |
| G4 | 77.60 | 122.78 | 10.07 | 59.60 | 6.67 | 3.408 | 20.17 | 0.195 | 3.72 | 8.57 | 26.07 | 28.63 | 84.26 | 302.48 |
| G5 | 74.70 | 113.76 | 8.35 | 63.05 | 6.76 | 3.015 | 18.83 | 0.283 | 3.97 | 7.36 | 26.48 | 30.60 | 47.60 | 155.68 |
| G6 | 75.40 | 122.45 | 9.71 | 59.04 | 7.64 | 3.692 | 16.90 | 0.256 | 4.83 | 7.71 | 25.02 | 25.68 | 48.92 | 193.09 |
| G7 | 75.90 | 127.74 | 10.97 | 59.41 | 6.57 | 3.582 | 16.42 | 0.270 | 4.31 | 9.21 | 26.51 | 23.75 | 57.53 | 246.83 |
| G8 | 72.40 | 138.68 | 10.35 | 70.88 | 7.80 | 4.801 | 17.30 | 0.198 | 4.74 | 9.84 | 23.96 | 26.52 | 74.35 | 286.28 |
| G9 | 70.90 | 164.72 | 10.54 | 65.94 | 7.12 | 4.172 | 17.49 | 0.244 | 5.30 | 16.43 | 22.00 | 27.89 | 62.69 | 228.23 |
| G10 | 72.40 | 125.49 | 9.74 | 54.17 | 7.32 | 3.312 | 16.90 | 0.174 | 4.55 | 9.97 | 23.72 | 28.55 | 99.95 | 337.13 |
| G11 | 73.20 | 123.06 | 10.79 | 55.11 | 6.13 | 3.094 | 17.88 | 0.275 | 4.52 | 6.89 | 23.96 | 27.60 | 53.40 | 194.53 |
| G12 | 65.90 | 113.06 | 8.93 | 55.98 | 7.46 | 3.115 | 18.20 | 0.227 | 3.87 | 6.96 | 26.10 | 28.54 | 39.01 | 137.50 |
| G13 | 76.10 | 121.83 | 10.04 | 57.88 | 6.62 | 3.179 | 16.84 | 0.225 | 4.93 | 7.50 | 21.29 | 30.95 | 66.67 | 215.12 |
| G14 | 74.50 | 143.02 | 10.23 | 69.54 | 7.99 | 4.755 | 19.35 | 0.203 | 4.77 | 11.16 | 31.00 | 30.37 | 95.95 | 317.89 |
| G15 | 69.80 | 157.75 | 10.90 | 67.52 | 8.10 | 5.105 | 19.21 | 0.149 | 5.92 | 11.20 | 24.97 | 31.72 | 100.33 | 314.33 |
| G16 | 77.90 | 136.23 | 10.14 | 64.11 | 7.77 | 4.241 | 17.84 | 0.185 | 5.35 | 8.16 | 26.96 | 32.24 | 101.66 | 314.22 |
| G17 | 57.20 | 248.05 | 8.07 | 78.29 | 3.87 | 2.015 | 9.04 | 0.246 | 5.28 | 30.94 | 39.01 | 29.97 | 32.22 | 108.16 |
| G18 | 64.70 | 165.24 | 8.15 | 66.91 | 7.02 | 3.231 | 16.15 | 0.250 | 5.78 | 12.80 | 21.49 | 31.81 | 85.95 | 267.86 |
| G19 | 67.80 | 135.74 | 7.88 | 63.70 | 6.55 | 2.783 | 20.90 | 0.170 | 5.25 | 10.59 | 27.74 | 29.76 | 62.29 | 211.21 |
| G20 | 73.20 | 136.25 | 7.12 | 59.25 | 5.53 | 1.949 | 13.84 | 0.159 | 4.68 | 13.70 | 27.27 | 30.79 | 52.75 | 169.93 |
| G21 | 80.70 | 129.65 | 11.84 | 62.56 | 8.46 | 5.229 | 19.55 | 0.234 | 3.82 | 11.31 | 24.43 | 28.50 | 62.99 | 222.92 |
| G22 | 70.80 | 158.80 | 9.14 | 64.15 | 7.86 | 3.852 | 21.05 | 0.138 | 5.44 | 10.07 | 28.93 | 31.32 | 100.33 | 319.26 |
| G23 | 67.50 | 126.94 | 10.37 | 66.77 | 7.15 | 4.115 | 22.16 | 0.213 | 5.48 | 8.13 | 21.51 | 24.36 | 68.87 | 283.28 |
| G24 | 75.00 | 116.49 | 9.98 | 57.20 | 7.64 | 3.678 | 16.26 | 0.249 | 4.46 | 10.00 | 27.85 | 29.46 | 66.66 | 225.94 |

| G25 | 76.60 | 133.73 | 10.10 | 63.48 | 7.08 | 3.778 | 14.68 | 0.222 | 4.51 | 11.36 | 25.23 | 28.26 | 71.21 | 256.13 |
| --- | --- | --- | --- | --- | --- | --- | --- | --- | --- | --- | --- | --- | --- | --- |
| G26 | 83.10 | 105.08 | 9.34 | 72.03 | 6.93 | 3.913 | 23.44 | 0.288 | 3.52 | 8.97 | 29.25 | 26.45 | 54.04 | 204.12 |
| G27 | 75.30 | 106.50 | 8.90 | 57.96 | 6.10 | 2.673 | 12.02 | 0.228 | 3.98 | 8.02 | 24.92 | 26.97 | 32.14 | 116.39 |
| G28 | 74.50 | 125.61 | 10.62 | 62.73 | 6.72 | 3.735 | 18.66 | 0.219 | 5.15 | 9.43 | 26.43 | 28.12 | 75.03 | 266.82 |
| G29 | 72.20 | 100.66 | 10.18 | 56.52 | 5.84 | 2.847 | 18.72 | 0.205 | 3.56 | 8.02 | 20.71 | 29.73 | 44.66 | 150.39 |
| G30 | 73.90 | 154.62 | 10.53 | 61.22 | 7.74 | 4.169 | 15.73 | 0.159 | 5.25 | 15.97 | 25.81 | 30.70 | 103.05 | 336.77 |
| G31 | 73.30 | 146.14 | 9.13 | 70.01 | 7.52 | 4.053 | 15.23 | 0.254 | 4.44 | 10.45 | 32.77 | 25.80 | 60.11 | 230.98 |
| G32 | 69.70 | 167.77 | 9.64 | 76.57 | 8.21 | 5.071 | 16.13 | 0.195 | 6.67 | 14.20 | 26.07 | 29.63 | 101.52 | 344.05 |
| G33 | 72.80 | 170.06 | 10.44 | 64.47 | 7.27 | 4.149 | 13.72 | 0.192 | 6.65 | 16.85 | 26.06 | 27.17 | 89.96 | 328.04 |
| G34 | 70.40 | 176.38 | 10.66 | 65.68 | 7.26 | 4.288 | 17.84 | 0.158 | 7.16 | 17.89 | 22.60 | 29.47 | 90.39 | 306.30 |
| G35 | 74.40 | 165.05 | 9.15 | 63.28 | 6.86 | 3.306 | 20.20 | 0.135 | 5.55 | 18.81 | 21.84 | 24.41 | 108.44 | 446.18 |
| G36 | 73.00 | 155.01 | 9.40 | 74.26 | 6.93 | 4.090 | 18.92 | 0.200 | 6.41 | 19.09 | 27.44 | 26.25 | 106.20 | 406.77 |
| G37 | 78.80 | 143.67 | 9.64 | 59.37 | 7.73 | 3.707 | 16.52 | 0.218 | 3.94 | 14.03 | 24.49 | 26.19 | 59.33 | 225.50 |
| G38 | 68.60 | 178.02 | 9.59 | 63.86 | 6.95 | 3.656 | 15.57 | 0.180 | 5.97 | 18.57 | 26.26 | 30.02 | 115.36 | 383.02 |
| G39 | 66.50 | 154.12 | 11.24 | 54.13 | 6.81 | 3.508 | 19.71 | 0.178 | 5.88 | 14.34 | 26.21 | 31.58 | 82.20 | 259.63 |
| G40 | 74.70 | 137.23 | 9.01 | 58.95 | 8.06 | 3.615 | 16.82 | 0.140 | 4.46 | 14.22 | 25.56 | 29.10 | 78.40 | 269.26 |
| G41 | 69.60 | 132.60 | 10.02 | 56.77 | 7.35 | 3.542 | 14.32 | 0.173 | 4.95 | 10.93 | 22.92 | 31.59 | 90.73 | 285.54 |
| G42 | 68.80 | 129.41 | 9.06 | 48.52 | 6.35 | 2.328 | 12.93 | 0.158 | 4.83 | 12.28 | 18.84 | 31.57 | 59.33 | 186.77 |
| G43 | 70.60 | 144.28 | 9.88 | 64.41 | 7.25 | 3.906 | 14.60 | 0.199 | 5.36 | 12.49 | 23.99 | 27.20 | 76.44 | 280.38 |
| G44 | 56.90 | 206.42 | 8.73 | 62.17 | 6.05 | 2.731 | 16.87 | 0.105 | 7.50 | 22.17 | 27.66 | 32.37 | 100.09 | 309.21 |
| G45 | 64.10 | 186.85 | 8.39 | 66.02 | 6.47 | 3.078 | 13.54 | 0.121 | 6.01 | 23.37 | 30.26 | 32.02 | 73.99 | 231.55 |
| G46 | 87.10 | 165.98 | 10.63 | 64.60 | 6.46 | 3.731 | 14.10 | 0.236 | 5.83 | 15.77 | 16.20 | 29.44 | 66.27 | 226.66 |
| G47 | 74.90 | 173.77 | 9.30 | 69.65 | 6.43 | 3.498 | 15.71 | 0.207 | 5.20 | 15.36 | 27.08 | 19.86 | 71.76 | 362.28 |
| G48 | 72.30 | 156.87 | 8.90 | 67.44 | 7.04 | 3.574 | 17.05 | 0.191 | 4.51 | 10.48 | 25.84 | 28.43 | 57.51 | 203.17 |
| G49 | 73.20 | 132.86 | 9.77 | 64.22 | 7.03 | 3.714 | 17.52 | 0.186 | 4.64 | 10.76 | 24.27 | 31.21 | 107.97 | 351.79 |
| G50 | 77.40 | 144.84 | 8.89 | 57.57 | 6.49 | 2.763 | 14.57 | 0.204 | 4.97 | 13.33 | 22.99 | 23.88 | 48.61 | 202.27 |

| G51 | 64.90 | 133.94 | 9.39 | 64.84 | 6.80 | 3.484 | 12.47 | 0.196 | 4.52 | 10.91 | 22.52 | 26.51 | 49.45 | 189.43 |
| --- | --- | --- | --- | --- | --- | --- | --- | --- | --- | --- | --- | --- | --- | --- |
| G52 | 79.00 | 117.86 | 9.99 | 61.10 | 6.75 | 3.468 | 16.07 | 0.271 | 3.77 | 5.21 | 28.30 | 26.66 | 66.60 | 251.12 |
| G53 | 73.70 | 146.08 | 10.65 | 66.13 | 6.67 | 3.886 | 18.82 | 0.246 | 4.46 | 12.96 | 24.97 | 29.67 | 68.44 | 229.01 |
| G54 | 72.30 | 135.12 | 11.15 | 63.45 | 7.06 | 4.070 | 19.70 | 0.276 | 4.85 | 9.71 | 23.39 | 26.14 | 85.83 | 326.48 |
| G55 | 80.20 | 110.38 | 9.52 | 59.74 | 7.39 | 3.479 | 19.32 | 0.263 | 3.77 | 7.24 | 28.38 | 26.87 | 52.40 | 193.19 |
| G56 | 91.50 | 145.97 | 10.20 | 65.24 | 7.15 | 3.980 | 17.94 | 0.279 | 4.08 | 15.25 | 23.53 | 25.42 | 66.99 | 263.15 |
| G57 | 69.20 | 130.59 | 9.47 | 57.72 | 6.58 | 3.012 | 17.82 | 0.137 | 5.02 | 8.57 | 18.63 | 28.07 | 72.56 | 258.36 |
| G58 | 72.30 | 139.90 | 10.38 | 66.43 | 7.23 | 4.099 | 20.57 | 0.143 | 4.82 | 9.67 | 22.58 | 31.63 | 127.78 | 402.11 |
| G59 | 72.10 | 114.28 | 10.35 | 59.57 | 8.13 | 4.175 | 20.80 | 0.237 | 3.55 | 6.25 | 32.19 | 27.90 | 67.79 | 241.13 |
| G60 | 70.50 | 154.93 | 9.57 | 63.21 | 7.82 | 3.915 | 15.50 | 0.138 | 5.59 | 15.19 | 28.58 | 31.58 | 92.10 | 290.57 |
| G61 | 66.70 | 115.34 | 9.00 | 61.78 | 7.79 | 3.703 | 13.49 | 0.271 | 4.58 | 9.12 | 24.74 | 25.17 | 42.15 | 169.04 |
| G62 | 68.40 | 128.88 | 9.78 | 65.20 | 8.07 | 4.313 | 20.94 | 0.235 | 4.82 | 9.05 | 26.46 | 30.30 | 112.41 | 369.65 |
| G63 | 70.20 | 175.72 | 10.52 | 66.02 | 6.62 | 3.885 | 15.75 | 0.181 | 8.42 | 16.88 | 24.78 | 26.46 | 118.41 | 447.51 |
| G64 | 69.60 | 180.95 | 11.39 | 78.65 | 8.36 | 6.253 | 16.81 | 0.138 | 5.97 | 16.42 | 23.18 | 31.31 | 114.04 | 364.63 |
| G65 | 70.40 | 131.52 | 10.66 | 58.63 | 8.30 | 4.317 | 17.48 | 0.204 | 5.63 | 10.30 | 24.33 | 28.15 | 80.84 | 286.61 |
| G66 | 75.10 | 182.16 | 10.57 | 71.44 | 7.93 | 5.006 | 15.40 | 0.285 | 6.07 | 15.50 | 24.66 | 27.43 | 90.34 | 328.74 |
| G67 | 49.50 | 196.77 | 7.05 | 57.09 | 3.11 | 1.026 | 8.42 | 0.119 | 5.50 | 28.65 | 28.15 | 30.24 | 30.93 | 102.50 |
| G68 | 72.70 | 110.37 | 10.48 | 68.65 | 7.34 | 4.338 | 16.18 | 0.269 | 4.45 | 6.35 | 25.52 | 26.07 | 50.47 | 194.49 |
| G69 | 73.40 | 210.85 | 10.32 | 77.77 | 6.59 | 4.401 | 15.07 | 0.108 | 5.83 | 15.85 | 20.90 | 31.92 | 139.84 | 437.76 |
| G70 | 70.50 | 123.20 | 9.76 | 54.99 | 6.49 | 2.921 | 15.99 | 0.228 | 4.39 | 10.21 | 23.50 | 29.03 | 67.14 | 233.03 |
| G71 | 74.00 | 123.51 | 11.23 | 56.63 | 7.06 | 3.737 | 18.92 | 0.234 | 4.78 | 8.27 | 21.83 | 28.76 | 54.60 | 195.30 |
| G72 | 80.50 | 109.98 | 10.45 | 60.99 | 7.39 | 3.918 | 17.50 | 0.231 | 3.94 | 8.33 | 22.63 | 27.61 | 53.75 | 195.09 |
| G73 | 79.50 | 126.36 | 9.69 | 57.25 | 6.09 | 2.803 | 12.79 | 0.214 | 4.28 | 13.92 | 17.87 | 31.34 | 52.48 | 166.72 |
| G74 | 72.30 | 123.62 | 10.20 | 58.59 | 6.45 | 3.221 | 14.61 | 0.281 | 5.19 | 10.81 | 21.44 | 30.22 | 47.23 | 154.98 |
| G75 | 70.20 | 136.81 | 10.43 | 68.42 | 8.46 | 5.051 | 19.66 | 0.151 | 5.77 | 10.13 | 22.27 | 31.65 | 99.57 | 313.34 |
| G76 | 71.70 | 125.20 | 9.93 | 66.77 | 6.47 | 3.654 | 18.00 | 0.108 | 5.59 | 8.60 | 22.03 | 31.41 | 89.59 | 286.23 |

| G77 | 68.30 | 108.42 | 10.14 | 50.81 | 4.51 | 1.975 | 12.99 | 0.249 | 4.90 | 5.35 | 19.32 | 25.66 | 43.44 | 169.08 |
| --- | --- | --- | --- | --- | --- | --- | --- | --- | --- | --- | --- | --- | --- | --- |
| G78 | 68.80 | 110.14 | 9.92 | 53.10 | 7.03 | 3.112 | 17.40 | 0.236 | 4.47 | 7.39 | 26.41 | 25.43 | 44.85 | 177.10 |
| G79 | 67.60 | 131.04 | 10.05 | 67.83 | 7.20 | 4.115 | 20.61 | 0.212 | 5.99 | 7.20 | 19.74 | 24.61 | 64.98 | 262.14 |
| G80 | 73.50 | 167.92 | 10.31 | 61.90 | 7.01 | 3.755 | 15.39 | 0.152 | 7.15 | 17.68 | 20.53 | 29.60 | 96.29 | 325.15 |
| G81 | 67.70 | 182.85 | 8.19 | 62.66 | 6.04 | 2.600 | 12.14 | 0.110 | 5.47 | 24.18 | 29.23 | 31.29 | 62.27 | 199.95 |
| G82 | 88.10 | 120.69 | 9.80 | 60.54 | 7.48 | 3.712 | 17.96 | 0.276 | 4.26 | 16.41 | 23.45 | 27.19 | 71.33 | 260.94 |
| G83 | 77.90 | 105.68 | 11.13 | 54.91 | 5.88 | 3.036 | 15.87 | 0.264 | 4.27 | 9.55 | 27.23 | 29.83 | 63.46 | 212.93 |
| G84 | 72.20 | 125.66 | 10.83 | 70.62 | 7.98 | 5.108 | 15.85 | 0.204 | 5.06 | 10.79 | 23.90 | 27.73 | 86.73 | 312.61 |
| G85 | 71.40 | 175.95 | 10.91 | 60.92 | 6.72 | 3.744 | 15.27 | 0.240 | 5.14 | 16.70 | 21.75 | 27.72 | 60.14 | 216.83 |
| G86 | 74.20 | 114.92 | 10.16 | 51.92 | 7.32 | 3.230 | 17.51 | 0.178 | 4.65 | 10.01 | 23.87 | 33.03 | 124.74 | 376.94 |
| G87 | 73.20 | 116.70 | 11.67 | 51.28 | 6.28 | 3.171 | 18.55 | 0.271 | 4.54 | 7.72 | 24.12 | 23.94 | 51.55 | 215.06 |
| G88 | 76.80 | 113.16 | 10.37 | 53.67 | 6.35 | 2.947 | 17.34 | 0.230 | 5.21 | 8.21 | 22.19 | 30.42 | 69.11 | 226.92 |
| G89 | 73.50 | 129.51 | 10.30 | 64.87 | 7.86 | 4.410 | 18.63 | 0.173 | 5.07 | 11.84 | 31.89 | 27.58 | 108.21 | 391.70 |
| G90 | 74.80 | 164.66 | 11.49 | 64.93 | 8.45 | 5.278 | 20.03 | 0.159 | 5.97 | 13.86 | 24.98 | 31.39 | 117.84 | 375.10 |
| G91 | 78.90 | 126.42 | 10.78 | 62.55 | 7.92 | 4.478 | 18.97 | 0.182 | 5.07 | 9.44 | 27.04 | 31.38 | 108.70 | 346.34 |
| G92 | 77.50 | 97.07 | 8.24 | 57.01 | 6.00 | 2.372 | 11.59 | 0.266 | 4.03 | 8.23 | 23.37 | 28.79 | 33.26 | 115.35 |
| G93 | 76.50 | 119.52 | 10.42 | 55.32 | 6.14 | 3.012 | 18.41 | 0.218 | 4.81 | 9.80 | 23.44 | 26.52 | 71.60 | 268.18 |
| G94 | 70.10 | 93.63 | 10.40 | 52.27 | 5.67 | 2.604 | 17.76 | 0.196 | 3.84 | 7.93 | 19.22 | 27.98 | 43.48 | 154.67 |
| G95 | 74.50 | 163.05 | 9.94 | 66.82 | 7.74 | 4.315 | 17.11 | 0.159 | 4.95 | 18.87 | 25.63 | 31.21 | 117.69 | 377.17 |
| Mean | 72.62 | 139.85 | 9.88 | 62.50 | 6.99 | 3.660 | 16.87 | 0.210 | 5.00 | 12.05 | 24.77 | 28.70 | 74.99 | 260.59 |
| SEm | 1.032 | 4.097 | 0.206 | 1.046 | 0.196 | 0.198 | 0.613 | 0.007 | 0.122 | 0.369 | 0.588 | 0.476 | 2.617 | 9.403 |
| CD (5%) | 2.898 | 11.505 | 0.579 | 2.938 | 0.552 | 0.555 | 1.721 | 0.02 | 0.343 | 1.036 | 1.651 | 1.336 | 7.347 | 26.403 |
| CD (1%) | 3.838 | 15.234 | 0.766 | 3.891 | 0.73 | 0.736 | 2.279 | 0.026 | 0.455 | 1.372 | 2.186 | 1.769 | 9.729 | 34.961 |
| CV | 3.254 | 7.203 | 6.082 | 4.533 | 6.509 | 14.054 | 9.267 | 8.838 | 7.332 | 8.409 | 6.356 | 5.202 | 11.004 | 9.414 |

Supplementary Table.4 Multi trait based stability index score of different models in 95 forage sorghum genotypes

| Genotype | MGIDI (Rank) | MTMPS (Rank) | MTSI (Rank) |
| --- | --- | --- | --- |
| G1 | 6.81(90) | 9.467(93) | 10.018(90) |
| G2 | 5.485(60) | 6.683(20) | 8.009(30) |
| G3 | 4.665(22) | 6.189(9) | 8.298(41) |
| G4 | 5.562(62) | 7.408(45) | 7.953(25) |
| G5 | 5.859(70) | 7.068(32) | 7.781(23) |
| G6 | 5.565(63) | 7.592(55) | 7.996(29) |
| G7 | 5.636(64) | 8.202(72) | 9.493(81) |
| G8 | 4.484(16) | 6.645(16) | 7.708(21) |
| G9 | 4.893(30) | 7.206(39) | 8.689(58) |
| G10 | 5.806(67) | 8.762(87) | 9.623(85) |
| G11 | 6.262(80) | 8.228(76) | 8.401(44) |
| G12 | 6.139(77) | 7.689(59) | 8.065(33) |
| G13 | 6.275(81) | 7.487(51) | 7.61(19) |
| G14 | 3.992(4) | 6.065(6) | 8.224(37) |
| G15 | 4.44(14) | 7.592(56) | 10.436(93) |
| G16 | 4.909(33) | 6.661(17) | 8.391(43) |
| G17 | 5.429(54) | 6.685(21) | 7.955(26) |
| G18 | 4.887(29) | 6.509(12) | 8.071(34) |
| G19 | 5.307(48) | 7.002(31) | 9.327(76) |
| G20 | 6.063(73) | 8.715(86) | 11.036(95) |
| G21 | 5.275(45) | 7.582(52) | 8.635(53) |
| G22 | 4.651(19) | 6.18(7) | 7.114(6) |
| G23 | 5.17(41) | 6.591(13) | 7.4(10) |
| G24 | 5.412(53) | 7.088(34) | 7.595(18) |
| G25 | 5.09(36) | 7.588(54) | 8.273(39) |
| G26 | 5.28(46) | 7.184(37) | 8.662(55) |
| G27 | 6.408(84) | 8.766(88) | 9.825(88) |
| G28 | 5.129(38) | 8.097(67) | 8.836(64) |
| G29 | 6.882(91) | 8.998(90) | 9.105(71) |
| G30 | 4.657(21) | 8.36(80) | 9.821(87) |
| G31 | 4.295(10) | 6.663(18) | 8.011(31) |
| G32 | 3.191(1) | 6.861(26) | 9.248(74) |
| G33 | 4.406(12) | 7.648(58) | 8.525(50) |
| G34 | 4.697(24) | 5.851(3) | 6.842(5) |
| G35 | 4.654(20) | 8.098(68) | 8.724(61) |
| G36 | 3.322(2) | 6.314(10) | 7.428(12) |
| G37 | 5.192(42) | 8.107(70) | 9.465(79) |
| G38 | 4.192(8) | 7.586(53) | 8.944(66) |
| G39 | 5.473(59) | 8.365(81) | 9.298(75) |
| G40 | 5.102(37) | 6.682(19) | 7.418(11) |
| G41 | 5.832(69) | 8.582(83) | 9.845(89) |

| G42 | 7.292(93) | 9.921(95) | 10.573(94) |
| --- | --- | --- | --- |
| G43 | 4.856(28) | 7.416(46) | 9.239(73) |
| G44 | 4.905(31) | 7.862(61) | 9.467(80) |
| G45 | 4.387(11) | 6.97(30) | 8.02(32) |
| G46 | 6.337(82) | 8.179(71) | 8.457(47) |
| G47 | 4.484(17) | 7.433(47) | 8.269(38) |
| G48 | 4.754(26) | 7.265(40) | 8.513(49) |
| G49 | 5.134(39) | 7.742(60) | 8.691(59) |
| G50 | 5.823(68) | 8.605(84) | 9.342(77) |
| G51 | 5.459(56) | 9.291(91) | 10.241(92) |
| G52 | 5.521(61) | 8.048(66) | 9(69) |
| G53 | 4.908(32) | 7.879(62) | 9.226(72) |
| G54 | 5.218(43) | 7.963(64) | 9.381(78) |
| G55 | 5.672(66) | 7.443(48) | 8.086(35) |
| G56 | 5.314(49) | 7.2(38) | 7.571(17) |
| G57 | 6.395(83) | 8.105(69) | 8.13(36) |
| G58 | 5.284(47) | 6.871(28) | 7.146(8) |
| G59 | 5.361(51) | 6.863(27) | 7.966(27) |
| G60 | 4.437(13) | 6.716(22) | 8.616(52) |
| G61 | 5.467(57) | 8.228(75) | 9.612(84) |
| G62 | 4.697(23) | 6.634(15) | 7.562(16) |
| G63 | 4.739(25) | 7.07(33) | 8.405(45) |
| G64 | 3.489(3) | 6.005(5) | 8.474(48) |
| G65 | 5.145(40) | 6.78(25) | 7.126(7) |
| G66 | 4.083(7) | 7.109(35) | 7.981(28) |
| G67 | 7.074(92) | 8.383(82) | 8.369(42) |
| G68 | 5.264(44) | 6.923(29) | 7.473(14) |
| G69 | 4.252(9) | 6.189(8) | 8.675(57) |
| G70 | 6.074(75) | 7.48(49) | 7.466(13) |
| G71 | 6.167(78) | 8.307(79) | 8.756(62) |
| G72 | 5.916(71) | 7.359(43) | 8.664(56) |
| G73 | 6.773(89) | 8.227(74) | 8.827(63) |
| G74 | 6.198(79) | 7.642(57) | 7.893(24) |
| G75 | 4.854(27) | 6.778(24) | 8.636(54) |
| G76 | 5.657(65) | 8.267(78) | 9.581(83) |
| G77 | 7.694(95) | 9.453(92) | 9.547(82) |
| G78 | 6.035(72) | 7.927(63) | 8.998(68) |
| G79 | 5.319(50) | 7.162(36) | 8.414(46) |
| G80 | 5.472(58) | 6.619(14) | 6.378(3) |
| G81 | 4.946(34) | 5.984(4) | 6.015(1) |
| G82 | 5.404(52) | 7.392(44) | 7.245(9) |
| G83 | 6.13(76) | 8.042(65) | 9.1(70) |
| G84 | 4.515(18) | 6.49(11) | 7.545(15) |
| G85 | 5.433(55) | 7.486(50) | 8.288(40) |
| G86 | 6.417(85) | 8.211(73) | 8.586(51) |
| G87 | 6.532(87) | 8.647(85) | 8.969(67) |

| G88 | 6.526(86) | 8.26(77) | 8.93(65) |
| --- | --- | --- | --- |
| G89 | 4.013(5) | 5.78(2) | 6.779(4) |
| G90 | 4.456(15) | 5.675(1) | 6.038(2) |
| G91 | 5.002(35) | 7.306(42) | 8.697(60) |
| G92 | 6.732(88) | 8.802(89) | 9.684(86) |
| G93 | 6.069(74) | 7.297(41) | 7.647(20) |
| G94 | 7.333(94) | 9.659(94) | 10.217(91) |
| G95 | 4.072(6) | 6.753(23) | 7.778(22) |

Supplementary Table.5 Factorial loadings after varimax rotation obtained in the factor analysis through MGIDI analysis in in 95 forage sorghum genotypes

| GEN | FA1 | FA2 | FA3 | FA4 | GEN | FA1 | FA2 | FA3 | FA4 |
| --- | --- | --- | --- | --- | --- | --- | --- | --- | --- |
| G1 | -1.532 | -0.012 | 1.635 | 1.171 | G51 | -2.342 | -0.865 | 2.369 | 0.578 |
| G2 | -2.815 | 0.043 | 2.938 | 1.525 | G52 | -3.281 | 0.335 | 3.120 | 0.052 |
| G3 | -4.505 | 0.232 | 3.911 | 1.992 | G53 | -3.446 | -0.549 | 2.679 | 0.573 |
| G4 | -3.615 | 0.461 | 2.696 | 1.458 | G54 | -4.013 | -0.662 | 1.866 | -0.015 |
| G5 | -2.682 | 0.844 | 3.844 | 1.052 | G55 | -3.353 | 0.794 | 3.562 | 0.237 |
| G6 | -3.149 | -0.048 | 2.556 | -0.034 | G56 | -3.777 | -0.816 | 2.295 | -1.138 |
| G7 | -3.187 | -0.476 | 2.241 | -0.762 | G57 | -2.763 | -0.274 | 1.075 | 2.013 |
| G8 | -4.099 | -0.860 | 2.671 | 0.283 | G58 | -4.478 | -0.353 | 2.002 | 2.691 |
| G9 | -3.325 | -1.493 | 2.009 | 0.064 | G59 | -4.067 | 1.025 | 4.289 | 0.998 |
| G10 | -3.389 | -0.018 | 1.849 | 2.044 | G60 | -3.521 | -0.932 | 3.203 | 2.342 |
| G11 | -2.750 | 0.162 | 1.746 | 0.327 | G61 | -2.621 | -0.297 | 3.018 | -0.137 |
| G12 | -2.523 | 0.937 | 3.418 | 1.434 | G62 | -4.493 | -0.002 | 3.242 | 1.877 |
| G13 | -2.903 | 0.143 | 1.493 | 1.436 | G63 | -3.855 | -3.130 | 1.047 | 0.832 |
| G14 | -4.555 | -0.362 | 4.076 | 1.335 | G64 | -5.149 | -2.279 | 2.339 | 1.326 |
| G15 | -4.571 | -0.954 | 2.398 | 2.046 | G65 | -3.919 | -0.519 | 1.950 | 1.140 |
| G16 | -4.223 | -0.150 | 2.754 | 2.024 | G66 | -4.243 | -2.260 | 2.423 | -0.603 |
| G17 | -0.438 | -3.694 | 6.299 | -0.009 | G67 | 0.884 | -2.627 | 3.326 | 2.481 |
| G18 | -2.778 | -1.152 | 2.632 | 1.785 | G68 | -3.514 | -0.170 | 2.919 | -0.468 |
| G19 | -2.686 | 0.034 | 3.869 | 2.258 | G69 | -4.237 | -2.731 | 1.628 | 2.081 |
| G20 | -1.398 | -0.203 | 3.511 | 2.260 | G70 | -2.560 | 0.039 | 1.993 | 1.351 |
| G21 | -4.531 | 0.083 | 2.391 | 0.044 | G71 | -3.301 | 0.105 | 1.390 | 0.713 |
| G22 | -4.055 | -0.262 | 3.652 | 2.746 | G72 | -3.432 | 0.341 | 2.097 | 0.254 |
| G23 | -3.881 | -0.658 | 1.913 | 0.294 | G73 | -2.030 | -0.328 | 0.926 | 1.145 |
| G24 | -3.289 | 0.288 | 3.007 | 0.979 | G74 | -2.385 | -0.374 | 1.554 | 0.518 |
| G25 | -3.262 | -0.566 | 2.490 | 0.534 | G75 | -4.619 | -0.585 | 2.178 | 2.173 |
| G26 | -3.940 | 0.671 | 4.510 | -0.414 | G76 | -3.447 | -0.446 | 1.763 | 2.567 |
| G27 | -1.730 | 0.217 | 2.609 | 0.381 | G77 | -1.278 | -0.231 | 0.295 | 0.400 |
| G28 | -3.568 | -0.338 | 2.417 | 0.795 | G78 | -2.634 | 0.397 | 2.551 | 0.564 |
| G29 | -2.380 | 0.854 | 1.699 | 1.457 | G79 | -3.682 | -0.915 | 1.583 | 0.235 |
| G30 | -3.875 | -1.068 | 2.235 | 1.848 | G80 | -3.359 | -2.193 | 0.708 | 1.515 |
| G31 | -3.392 | -0.680 | 4.757 | -0.259 | G81 | -1.664 | -1.873 | 3.588 | 2.274 |
| G32 | -4.401 | -2.050 | 3.136 | 0.984 | G82 | -3.595 | -0.313 | 2.266 | -0.248 |
| G33 | -3.526 | -2.283 | 1.943 | 0.477 | G83 | -2.789 | 0.330 | 2.086 | 0.704 |

| G34 | -3.751 | -2.231 | 1.414 | 1.309 | G84 | -4.327 | -0.954 | 2.352 | 0.459 |
| --- | --- | --- | --- | --- | --- | --- | --- | --- | --- |
| G35 | -3.724 | -1.822 | 1.691 | 1.226 | G85 | -2.859 | -1.640 | 1.478 | 0.021 |
| G36 | -4.096 | -2.193 | 3.103 | 0.534 | G86 | -3.719 | 0.438 | 1.600 | 3.179 |
| G37 | -3.210 | -0.399 | 2.655 | 0.206 | G87 | -2.938 | -0.004 | 1.214 | -0.383 |
| G38 | -3.465 | -1.936 | 2.468 | 1.839 | G88 | -2.837 | 0.201 | 1.198 | 1.433 |
| G39 | -3.302 | -0.643 | 1.840 | 2.238 | G89 | -4.524 | -0.538 | 3.632 | 1.402 |
| G40 | -3.343 | -0.199 | 2.928 | 1.974 | G90 | -5.087 | -1.167 | 1.998 | 1.857 |
| G41 | -3.127 | -0.314 | 1.737 | 2.315 | G91 | -4.588 | -0.043 | 2.537 | 1.902 |
| G42 | -1.630 | -0.089 | 0.870 | 2.599 | G92 | -1.503 | 0.455 | 2.496 | 0.556 |
| G43 | -3.252 | -1.195 | 2.200 | 0.703 | G93 | -3.000 | -0.157 | 1.467 | 0.735 |
| G44 | -2.384 | -2.527 | 2.738 | 3.341 | G94 | -2.103 | 0.708 | 0.955 | 1.334 |
| G45 | -2.204 | -2.028 | 3.899 | 2.447 | G95 | -4.168 | -1.301 | 2.769 | 1.974 |
| G46 | -3.041 | -1.821 | 0.169 | -0.213 |  |  |  |  |  |
| G47 | -3.223 | -2.309 | 2.870 | -1.119 |  |  |  |  |  |
| G48 | -2.978 | -0.542 | 3.440 | 0.970 |  |  |  |  |  |
| G49 | -3.768 | -0.310 | 2.426 | 2.124 |  |  |  |  |  |
| G50 | -2.205 | -0.984 | 1.935 | -0.038 |  |  |  |  |  |
